# Systematic Evidence Map for the Per- and Polyfluoroalkyl Substances (PFAS) Universe

**DOI:** 10.1021/EHP.6c00127

**Published:** 2026-04-19

**Authors:** Avanti V. Shirke, Elizabeth G. Radke, Ryan Jones, Barrett D. Allen, Cynthia J. Lin, Amanda Ross, Nicole Vetter, Courtney Lemeris, Pamela Hartman, Sorina Eftim, Arun Varghese, Robyn Blain, Heidi Hubbard, Antony J. Williams, Kristina A. Thayer, Laura M. Carlson

**Affiliations:** † Center for Public Health and Environmental Assessment, Chemical and Pollutant Assessment Division (CPAD), 314974 U.S. EPA, Washington, District of Columbia 20460, United States; ‡ Center for Public Health and Environmental Assessment, Health and Environmental Effects Assessment Division (HEEAD), 314974 U.S. EPA, Durham, North Carolina 27711, United States; § 486920 ICF, Fairfax, Virginia 22031, United States; ∥ Center for Computational Toxicology and Exposure (CCTE), U.S. EPA, Durham, North Carolina 27713, United States; ⊥ Formerly Center for Public Health and Environmental Assessment, Chemical and Pollutant Assessment Division (CPAD), U.S. EPA, Durham, North Carolina 27713, United States

## Abstract

**BACKGROUND:** Per- and polyfluoroalkyl substances (PFAS) are a research priority for the U.S. Environmental Protection Agency (EPA). Because PFAS include thousands of structurally diverse chemicals, there is a pressing need for identifying what data are available to assess the human health hazard of these compounds. **OBJECTIVES:** We used systematic evidence map (SEM) methods to summarize the available epidemiological and mammalian bioassay evidence for ∼14,735 chemicals identified as PFAS by EPA’s Center for Computational Toxicology and Exposure (CCTE). This work is a continuation of our previous 2022 and 2024 SEMs that inventoried evidence on a separate set of ∼500 PFAS. The Comprehensive PFAS Dashboard includes evidence identified from our past SEMs and completed EPA assessments. **METHODS:** We conducted literature searches from peer-reviewed and gray literature sources to identify, screen, and inventory mammalian bioassay and epidemiological literature. A combination of manual review and machine learning software were utilized. A diverse array of potentially relevant supplemental content was also tracked, including mechanistic data, exposure-only studies, and studies informing chemical toxicokinetics and clearance. For each study meeting predefined population, exposure, comparator, and outcome (PECO) criteria, experimental design details and health end points evaluated were summarized in interactive web-based literature inventory visuals. Epidemiology studies and animal bioassay studies with ≥21-day exposure duration or reproductive/developmental study design proceeded to undergo a study evaluation for risk of bias and sensitivity, as well as detailed extraction of health end point data. Underlying data are publicly available and can be downloaded. **RESULTS:** Scientific database searches retrieved 152,205 references. After full-text screening, there were 347 mammalian bioassay and 44 epidemiological studies that met PECO criteria. The mammalian bioassay and epidemiological evidence assessed 99 and 30 individual PFAS, respectively (*n* = 18 PFAS with both). The epidemiological evidence assessed 15 health systems and the mammalian bioassay evidence assessed 16 health systems. **DISCUSSION:** Results from our 2022 and 2024 SEMs and completed EPA assessments are compiled into Comprehensive PFAS Dashboard. This dashboard is a resource for better understanding the currently available PFAS human health hazard data. It can be used as a tool for researchers and regulators interested in PFAS data gaps and research needs. Across all the data sources compiled into the Comprehensive PFAS Dashboard, only 1.4% (214/14,735) of PFAS had any mammalian bioassay or epidemiological data available. The vast majority of PFAS lack publicly available information about the potential human health effects of exposure to these chemicals.

## Introduction

Per- and polyfluoroalkyl substances (PFAS) are persistent organohalogen contaminants to which there is widespread human exposure globally.^1^ Many organizations and researchers have proposed definitions for what constitutes a PFAS.^2–7^ The specific structural features considered in the definition influence the proposed size of the PFAS chemical class, which includes thousands of unique chemicals. For this research project, we used the PFASSTRUCTv5 list from the EPA Comptox Chemicals Dashboard (CCD), which consists of compounds identified as PFAS.^8^ In brief, Gaines et al.^9^ describes the challenges of defining PFAS, and this list uses four fluorinated substructures and a 30% fluorine percentage.^3,9^ Specific criteria are further described in the PFASSTRUCTv5 list details on the CCD and in the accompanying supplemental publications.^3,8,9^

Human exposure to PFAS is ubiquitous and there is tremendous interest in understanding health effects related to PFAS exposure.^1,10,11^ In 2024, the EPA promulgated its first-ever national legally enforceable drinking water standard (National Primary Drinking Water Regulation [NPDWR]) for six PFAS to protect Americans from pollution in drinking water. The critical health effects that supported these regulations included immune, developmental, metabolic, hepatic, thyroid, and cancer. After consideration of public comments on the proposed EPA (2023[2644]) maximum contaminant levels (MCLs), the EPA finalized MCLs for perfluorooctanoic acid (PFOA) and perfluorooctanesulfonic acid (PFOS) at 4.0 ng/L in 2024.^12^ In the same NPDWR, the EPA finalized MCLs for three additional PFAS: perfluorononanoic acid (PFNA), perfluorohexanesulfonic acid (PFHxS), and perfluoro-2-methyl-3-oxahexanoic acid (GenX chemicals) at 10 ng/L each, and an MCL for mixtures of any two or more of the following PFAS: PFNA, PFHxS, PFBS, and GenX chemicals (Hazard Index of 1).^12,13^ PFOS and PFOA were also recently designated as hazardous substances under the Comprehensive Environmental Response, Compensation, and Liability Act (CERCLA) Superfund legislation^14^ and the agency updated its recommendations on destroying and disposing of PFAS.^15^ In addition to these EPA actions, other health agencies including the International Agency for Research on Cancer (IARC) have evaluated specific PFAS for carcinogenicity, classifying PFOA as group 1 carcinogenic to humans and PFOS as possibly carcinogenic to humans (group 2B),^16,17^ and the Agency for Toxic Substances and Disease Registry has developed a toxicological profile for select perfluoroalkyls.^18^ These actions signal the importance for understanding the human health effects of PFAS exposure.

Toward the goal of better understanding the human health effects of PFAS exposure, several groups have developed systematic evidence maps (SEMs) that collate human and animal data available on lists of PFAS, including a group of 29 PFAS (PFAS-Tox Database)^19–21^ and the EPA’s Office of Research and Development (ORD) SEMs for ∼500 PFAS.^22–25^ This manuscript represents the final installment of the series of ORD PFAS evidence maps that searched for human health hazard information on all 14,735 chemicals identified as PFAS in the CCD “PFASSTRUCTV5-August 2022” list^8,9^ (Excel Table AS1) that have not been previously investigated by EPA (see Table S1). Of note, this effort does not update the literature searches of previous SEMs, and users should refer to each parent project for details about literature search strategies, scoping criteria, and data extraction methods.

These SEMs are large data repositories, with interactive visualizations and downloadable data sets that are a resource for PFAS researchers for comparison of the available data for different PFAS across this diverse chemical class. In parallel, ORD CCTE researchers have been testing various PFAS in high-throughput toxicity screens,^26–32^ including developmental neurotoxicity (DNT)^33,34^ to help facilitate additional analyses for grouping PFAS.^35^ The goal of the evidence maps is to utilize systematic review methods to “catalog the available hazard information and critically evaluate studies, but not to synthesize the data or reach hazard conclusions on the causal relationships between PFAS exposures and health effects.”^25^ By collating the evidence in interactive and filterable visuals, the hope is that researchers and regulators can use these data sets to inform PFAS analysis questions on risk assessments, PFAS categorization, or novel research questions. These evidence maps represent an important early step in identifying research needs and data gaps for understanding PFAS toxicity.

## Methods

### Workflow Overview

We followed the same systematic review methods to compile this systematic evidence map as the methods we used from our earlier 150+ PFAS SEMs^22–24^ and the Expanded PFAS SEM^25^ because this project is a continuation of that work. The methods are reproduced below and adjusted only as appropriate to meet the needs of this evidence map (e.g., chemical names, literature search dates). Literature screening, study evaluation, and data extraction methods are described in greater detail in the Supplemental file under section “Systematic Evidence Map Methods.” These methods follow the ORD Staff Handbook for Developing IRIS Assessments^36^ (referred to as the “IRIS Handbook”) and a published evidence map template^37^ that outline the systematic review methods used to develop the evidence map.

The semiautomated literature search and prioritization methods used in this SEM are novel, as they were developed to reduce the level of manual effort associated with identifying and processing searches for thousands of PFAS simultaneously. Because of their novelty and deviation from the methods used in our previous work, we describe these specific methods in greater detail in the “Literature Search” and “Literature Prioritization” sections below.

In brief, literature searches for peer-reviewed and gray literature were conducted using chemical synonyms, as identified in the CCD. Literature search results were prioritized for relevance to human health and screened using populations, exposures, comparators, and outcomes (PECO) criteria (presented in Table 1). In addition to PECO-relevant studies, studies that did not meet PECO criteria but contained “potentially relevant” information (e.g., mechanistic or pharmacokinetic data, non-PECO-relevant route of exposure) were tracked and tagged as “potentially relevant supplemental material” during the literature screening process. “Potentially relevant supplemental material” was tagged by category, as outlined in Table 2.

**Table 1:** Populations, Exposures, Comparators, and Outcomes (PECO) Criteria for PFAS Universe Screening (Reprinted from Shirke et al.^25^)

PECO element	Evidence
Populations	**Human:** Any population and lifestage (occupational or general population, including children and other sensitive populations).
	**Animal:** Nonhuman mammalian animal species (whole organism) of any lifestage (including preconception, *in utero*, lactation, peripubertal, and adult stages). Transgenic mammalian model systems will be tagged as “potentially relevant supplemental material.” Screener note: Mechanistic information including in vitro assays will be tagged as “potentially relevant supplemental material.”
Exposures	**Relevant forms**: Please refer to the Master List of included PFAS (Excel Table AS1). Many common names and synonyms should appear as keyword green highlighting. For title and abstract screening, include studies without an abstract (i.e., title only) in which the title mentions PFAS (or other words such as perfluorinated) but does not mention specific chemicals.
	**Human:** Any exposure to PFAS via the oral and inhalation routes. Studies will also be included if biomarkers of PFAS exposure are evaluated (e.g., measured PFAS in tissues or bodily fluids) but the exposure route is unclear or reflects multiple routes. Other exposure routes, including dermal will be tracked during title and abstract screening and tagged as “potentially relevant supplemental material.”
	**Animal**: Any exposure to PFAS (including mixtures containing PFAS) via the oral and inhalation routes. Studies involving exposures to mixtures will be included only if they include an arm with exposure to a PFAS alone. Other exposure routes, including dermal or injection, will be tracked during title and abstract screening and tagged as “potentially relevant supplemental material.”
Comparators	**Human:** A comparison or referent population exposed to lower levels (or no exposure/exposure below detection limits) of PFAS, or exposure to PFAS for shorter periods of time. However, worker surveillance studies are considered to meet PECO criteria even if no referent group is presented. Case reports describing findings in 1–3 people in nonoccupational or occupational settings will be tracked as “potentially relevant supplemental material.”
	**Animal**: A concurrent control group exposed to vehicle only treatment and/or untreated control (control could be a baseline measurement). Acute toxicity studies without a control group are considered to meet PECO criteria if the outcome is mortality and the baseline of alive can be used as the comparator.
Outcomes	**Human and Animal**: All health outcomes (both cancer and noncancer).

**Table 2:** Major Categories of “Potentially Relevant” Supplemental Material (Reprinted from Shirke et al. 2024).^25^,^a^

Category	Description
In vitro, ex vivo, or in silico “mechanistic” studies	In vitro, ex vivo, or in silico studies reporting measurements related to a health outcome that inform the biological or chemical events associated with phenotypic effects, in both mammalian and nonmammalian model systems.
*Absorption, distribution, metabolism, and excretion* (ADME)	ADME studies are primarily controlled experiments in which defined exposures usually occur by intravenous, oral, inhalation, or dermal routes, and the concentration of particles, a chemical, or its metabolites in blood or serum, other body tissues, or excreta are then measured. These data are used to estimate the amount absorbed (A), distributed to different organs (D), metabolized (M), and/or excreted/eliminated (E) through urine, breathe, feces, etc.
	• ADME data can also be collected from human subjects who have had environmental or workplace exposures that are not quantified or fully defined. However, to be useful such data must involve either repeated measurements over a time period when exposure is known (e.g., is zero because previous exposure ended) or time- and subject-matched tissue or excreta concentrations (e.g., plasma and urine, or maternal and cord blood).
	• ADME data, especially metabolism and tissue partition coefficient information, can be generated using in vitro model systems. Although in vitro data may not be as definitive as in vivo data, these studies should also be tracked as ADME. For large evidence bases it may be appropriate to separately track the in vitro ADME studies.
	Note: Studies describing environmental fate and transport or metabolism in bacteria are not tagged as ADME.
Classical pharmacokinetic (PK) model studies, or physiologically based pharmacokinetic (PBPK) model studies	Classical Pharmacokinetic (PK or Dosimetry Model Studies: Classical PK or dosimetry modeling usually divides the body into just one or two compartments, which are not specified by physiology, wherein movement of a chemical into, between, and out of the compartments is quantified empirically by fitting model parameters to ADME data.
	Physiologically based Pharmacokinetic (PBPK or Mechanistic Dosimetry Model Studies: PBPK models represent the body as various compartments (e.g., liver, lung, slowly perfused tissue, richly perfused tissue) in order to quantify the movement of chemicals or particles into and out of the body (compartments) by defined routes of exposure, metabolism and elimination, and thereby estimate concentrations in blood or target tissues.
Nonmammalian model systems	Studies in nonmammalian model systems, e.g., *Xenopus,* fish, birds, *C. elegans*.
Transgenic mammalian model systems	Transgenic studies in mammalian model systems.
Nonoral or noninhalation routes of administration	Studies in which humans or animals (whole organism) were exposed via a nonoral or noninhalation route (e.g., injection, dermal exposure).
Exposure characteristics (no health outcome assessment)	Exposure characteristic studies which include data that are unrelated to health outcomes, but which provide information on exposure sources or measurement properties of the environmental agent (e.g., demonstrate a biomarker of exposure).
Mixture studies	Mixture studies that are not considered PECO relevant because they do not contain an exposure or treatment group assessing only the chemical of interest. This category is generally used for experimental studies and generally does not apply to epidemiological studies in which the exposure source may be unclear but is used for epidemiological studies that only report associations based on ∑PFAS.
Case reports	Case reports describing health outcomes after exposure will be tracked as potentially relevant supplementary material when the number of subjects is ≤ 3.
Records with no original data	Records that do not contain original data, such as other agency assessments, informative scientific literature reviews, editorials or commentaries.
Meta-analysis	Epidemiological analyses that assess and synthesize quantitative data from previous human research studies that are PECO relevant.
Conference abstracts	Records that do not contain sufficient documentation to support study evaluation and data extraction.
European Chemicals Agency (ECHA) read-across	Data from ECHA on a nonrelevant chemical that makes inferences about a relevant PFAS.
Presumed duplicate	Duplicate studies (e.g., published vs unpublished reports) identified during data extraction and study quality evaluation.

a “Potentially relevant” supplemental material are studies that do not meet the PECO criteria but may still contain information on interest that was tracked during screening. Note: The definitions in the table follow standard template language^25,37^ used in systematic evidence maps developed by the EPA and have only been adjusted, where appropriate, for the specific needs of this SEM.

Following literature screening, studies meeting PECO criteria underwent a literature inventory-level manual data extraction in which basic study information was summarized (and described further in the Supplemental file, under section “Distiller Literature Inventory”). The literature inventory was used to prioritize mammalian bioassay studies with specific exposure designs. Specifically, studies assessing an exposure duration of ≥21 days or reproductive/developmental design were moved forward for study evaluation to explore potential risks of bias and insensitivity that may affect interpretation of study results. All epidemiological studies proceeded to study evaluation and data extraction.

Complex projects summarizing large manually curated databases will inevitably contain some data entry errors, even when quality control measures are taken. As errors are identified and corrected, they will be recorded in a changelog and added to the Health Assessment Workspace Collaborative (HAWC) project page for this publication.^38^ HAWC is a free and open-source web-based software application that facilitates the management of literature assessments for environmental pollutants. The study counts and figures presented in this paper represent a snapshot in time of a repository that may be updated as future analyses are conducted. For the most up-to-date and accurate information, users should consult the web-based inventory and project pages (https://hawc.epa.gov/assessment/100500347/).^38^

### Literature Search

EPA information specialists developed simple syntax literature search strings for all ∼14,735 PFAS in the universe list using the chemical name, synonyms, and trade names, as identified in the CCD “PFASSTRUCTv5” list^8,9,39^ (Excel Table AS1). After removing chemicals that had been previously assessed by past ORD SEMs^22–25^ or assessments (Table S1), 14,229 chemicals remained. EPA information specialists then used a script to automate and submit the search strings in rapid succession, returning a count of identified results. Searches were conducted in September 2022. The automated script used search terms for each chemical as indexed in the CCD to perform a search in Web of Science and PubMed. Using these search terms unaltered occasionally returns a false positive, so a team of librarians examined the terms for each chemical that returned at least one result in the first phase, making whatever adjustments were needed to ensure no false positives remained. Because of the large number of chemicals retrieving zero results (*n* = 12,275 chemicals), and the level of effort associated with manually annotating each null search result, chemicals that returns no results in the first phase are not displayed in the Excel supplemental files. EPA information specialists manually reviewed the results to move forward any chemicals with at least one reference identified for the next round of more tailored literature searches.

In the second stage of the literature search, EPA information specialists manually resubmitted each search for PFAS that returned nonzero counts (*n* = 1,954 chemicals), this time making syntactical adjustments to the search terms as needed (Excel Table AS2). The second stage searches were conducted between October and December 2022 (Excel Table AS2). For example, some databases would automatically break phrases into individual search terms unless the search query was manually altered to preclude this behavior, which would result in a false positive for the first phase that would be eliminated in the second phase. This two-tiered semiautomated approach allowed EPA information specialists to focus their manual efforts only on chemicals that had at least some results. All search results from the second stage are presented in Excel Table AS2. Instances for which false positives from the first stage were identified for a particular chemical during the second stage are noted as having zero results. A subset of chemicals (*n* = 1,625) from the second stage of the literature search were prioritized as the final list of chemicals carried forward in this SEM (Excel Table AS3). Chemicals were deprioritized if they were chemical forms already covered by existing assessments or if they were associated with medical applications like anesthesia (enflurane, isoflurane, halothane, desflurane).

#### Other Sources Consulted

Although the literature search strategies were intentionally broad, some studies may not have been captured if they were not indexed in the databases noted above. Because of the size of this chemical list, and given that searching other sources is a labor-intensive process, we needed to adopt a pragmatic approach. Based on our previous PFAS evidence maps,^22,23,25^ literature searches of the European Chemicals Agency (ECHA) database^40^ were most likely to return PECO-relevant references that were not captured by our primary literature searches in PubMed and Web of Science. Therefore, for this SEM, we limited the other gray literature sources consulted and focused solely on ECHA, which is a limitation of the current approach.

- ECHA registration dossiers (Excel Table AS4)
- Reference lists from all PECO-relevant mammalian bioassay and epidemiological studies identified in the database searches (Excel Table AS5)
- Expert-identified references that were not included in previous literature searches but were identified during the data extraction process of other PECO-relevant studies (Excel Table AS6)

##### Prioritizing Chemicals for ECHA Searches

A data pull batch search of the “PFASSTRUCTv5” list (Excel Table AS1) and the EPA Toxicity Value Database (ToxValDBv9.4) from CCD^41,42^ was performed in September 2023 and filtered by studies that had ECHA records/values available. There were ∼161 chemicals from the universe list that had ECHA entries. These were then deduplicated against the PFAS 150+ and Expanded PFAS SEM chemical lists,^22,25^ which left a total of 80 considered chemicals. The toxicity data sourced from the ECHA Web site and listed in the CCD represent a snapshot in time. Thus, it is possible that additional records have been added since the time the query was initially conducted. Therefore, for chemicals that had at least one study identified during literature inventory, individual ECHA searches were performed for those chemicals that were not already on the above-noted list (*n* = 80 chemicals). In total, 138 chemicals were searched in ECHA, 68 of which had toxicological information available, resulting in 815 individual references for review. Further detailed methods and documentation are available in the “ECHA Retrieval Instructions” section in the Supplemental file.

### Literature Prioritization

The comprehensive literature search was designed to avoid omitting relevant results, however, only a fraction of the literature captured was directly relevant to the PECO criteria. To reduce screening of irrelevant results, a combined approach using SWIFT-Review,^43^ supervised machine learning, and keyword analysis was applied to prioritize literature (visual workflow overview provided in Figure 1). The use of automated prioritization methods to limit the screening of nonrelevant studies is currently a mainstream practice in systematic reviews.^44^

**Figure 1:**
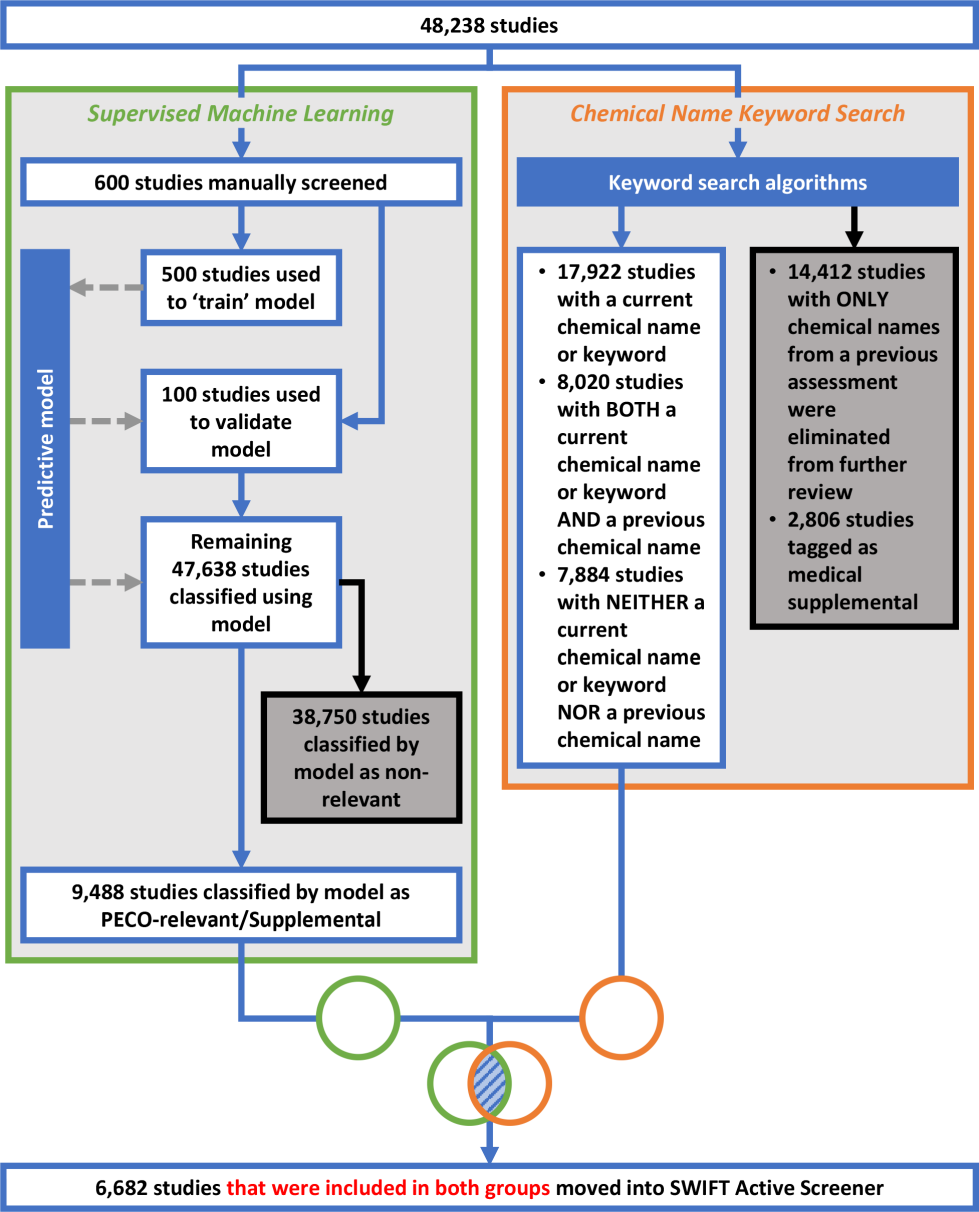
Literature prioritization overview visual flowchart of the supervised machine learning and chemical name keyword searches used to prioritize references from the large number of references identified for the PFAS universe SEM. Note: PECO = Population, Exposure, Comparator, Outcome.

Search results were stored in EPA’s Health and Environmental Research Online (HERO) database.^45^ After deduplication in HERO using unique identifiers (e.g., PMID, WoSID, or DOI) and citations, the references went through an additional round of deduplication using ICF’s DeDuper tool (described in detail in the Supplemental file, under section “DeDuper”), which uses a two-phase approach to identify duplicates by 1) locating duplicates using automated logic and 2) employing machine learning built from Python’s Dedupe package to predict likely duplicates that are then verified manually.^46^ Following deduplication, SWIFT-Review software^43^ was used to identify which of the unique references were most relevant for human health risk assessment. SWIFT-Review was used to filter the unique references based on the software’s preset “evidence stream” filters.^47^ References are tagged to a specific evidence stream if the search terms from that evidence stream appear in the title, abstract, keyword, and/or Medical Subject Headings (*MeSH*) fields of that reference. For this SEM, the following SWIFT-Review evidence stream filters were applied: human, animal models for human health, and in vitro studies. Studies not retrieved using the search strategies were not considered further.

Automated screening tools such as SWIFT-Active Screener^48^ use a process called active machine learning that has the advantage of combining the process of screening and algorithm training. Rather than introduce the full set of studies returned by SWIFT-Review (*n* = 48,238) for screening in SWIFT-Active Screener, two preprioritization steps were used to limit the number of studies upfront.

First, all references returned by SWIFT-Review were run through an ICF-developed predictive model^49^ using the machine learning-based bioBERT algorithm^50,51^ to classify studies as being candidates for further screening. Supervised machine learning algorithms require a training data set to build a predictive model and a validation data set to measure the performance of the predictive model. To build the training and validation data sets, 600 references were randomly selected from our unclassified search results (Excel Table BS1) for manual screening and labeled as PECO relevant, potentially relevant supplementary material, or not relevant. Of these labeled studies, 500 were reserved for the training data set (Excel Table BS2), while the remaining 100 (Excel Table BS3) were used to validate the model and determine the probability cutoff to ensure that 95% of the relevant studies would be captured. By combining the results of a binary predictive model for PECO relevance and a separate binary predictive model for potentially relevant supplementary material, the model classified the remaining studies as either PECO relevant, potentially relevant supplementary material, or not relevant.

Second, all references returned by SWIFT-Review received keyword analysis of title and abstracts. Keyword sets using chemical names and synonyms were created to identify references for chemicals previously reviewed in the PFAS 150 SEM and Expanded PFAS SEM or recently published/ongoing assessments (Table S1), references for chemicals relevant to the current assessment, and references that focused on medical use of chemicals. PFAS with medical use were deprioritized because the focus of this SEM is on PFAS arising from environmental exposure. Moreover, pharmaceuticals and other medical devise usages undergo their own evaluation and registration process by the Food and Drug Administration. Furthermore, the medical use references added substantially to the screening level of effort,^22^ and were deprioritized in some of the earlier experimental testing work for PFAS due to limited commercial availability, and failing analytical quality control.^52^ Therefore, they were deprioritized and considered beyond the scope of the current evidence map and were set aside and tagged in HERO as “Medical supplemental” (*n* = 2,806). Interested readers can download a list of references from the project page and are available in Excel Table AS7. The remaining references moved forward only if they met the criteria of a current chemical name, a current chemical name and a previous chemical name, or had neither a current nor a previous chemical name. References with only chemical names from a previous assessment or SEM were eliminated from further review.

The preprioritized references identified as PECO-relevant/potentially relevant supplementary material during supervised machine learning and met the keyword criteria were moved into SWIFT-Active Screener. Studies were manually screened by two independent reviewers. A 95% recall threshold was set, implying that 95% of the relevant studies would be captured by the screening process. Active screening tools iteratively build and refine predictive models as screeners label studies; the tools recommend that the screening process end when the recall threshold has been met. The screening process was designed to prioritize records that appeared to meet PECO criteria or included potentially relevant supplementary material content based on title/abstract (TIAB) content (i.e., both types of records were screened as “include” for active-learning purposes). Studies were screened in SWIFT-Active Screener until the 95% threshold was met. This threshold is comparable to human error rates^43,53,54^ and is used as a metric to evaluate machine learning performance. Any studies in “partially screened” status at the time of reaching the 95% threshold were fully screened.

As a final step, to ensure that relevant study recall targets were met and to pick up as many remaining relevant studies as possible from unscreened studies, the SWIFT-Active screened references were used to build a final supervised machine learning predictive model using the bioBERT algorithm.^49^ Deprioritized studies from the first supervised machine learning and any unscreened studies from SWIFT-Active screening were run through machine learning. Any deprioritized or unscreened study with a probability of relevance >0.5, or with a greater chance of being relevant than nonrelevant, was moved to TIAB screening in DistillerSR. A final supervised machine learning predictive model in the Document Classification and Topic Extraction Resource (DoCTER)^55^ was applied to all unscreened studies that met the keyword-based exclusion rule detailed during the prioritization step.

Following screening in SWIFT-Active Screener, studies identified as PECO-relevant or potentially relevant supplementary material were imported into DistillerSR for more specific title-abstract screening (i.e., to separate studies meeting PECO criteria from those that were identified as supplemental content, tag specific categories of supplemental content as outlined in Table 2, tag chemical name, etc.). Each reference was manually reviewed by two independent reviewers. These methods are described in detail in the Supplemental file under section “Literature Screening.” Studies meeting PECO criteria followed the same methods as were used in our previous SEMs^22–25^ for subsequent full-text screening, study evaluation, and data extraction. These methods are described in greater detail in the Supplemental file under section “Systematic Evidence Map Methods.” Records identified through the ECHA retrieval process were imported directly into DistillerSR for full-text screening.

### Literature Inventory

Studies that met PECO criteria following full-text review were inventoried using custom forms (see the Supplemental file under section “Distiller Literature Inventory SOP for PFAS”). As described in previous publications,^22–25^ and in greater detail in the Supplemental file (under subsection “Distiller Literature Inventory Universe Evidence Map”), for mammalian bioassay studies the inventory collects study summary information: PFAS assessed, study type [acute (<24 h), short-term (1–30 days), subchronic (30–90 days), chronic (>90 days), developmental, peripubertal, multigenerational], route of exposure, species, sex, and health system(s) assessed. Table S2 contains categorization details for grouping end points by health effect category.

For epidemiology studies, the following study summary information was captured in a literature inventory: PFAS assessed, sex, population, study design (Table S3), exposure measurement (e.g., blood, feces), and health system(s) assessed. For epidemiology studies, the literature inventory was a high-level summary that did not confirm results relevant for full data extraction. As a result, more health outcomes may have been summarized at this level compared with the subsequent data extraction step. Summaries were extracted into DistillerSR by one team member, and the extracted data were checked for quality by at least one other team member. These data are available in the interactive Tableau dashboard^56^ and for download as an Excel file.

### Study Evaluation and Data Extraction

As noted earlier, a literature inventory was used to prioritize mammalian bioassay studies with exposure to PFAS for repeat dose studies of 21 days and longer duration, or with study designs focused on exposure windows targeting reproduction and development. Studies meeting these exposure timing and duration parameters were moved forward for study evaluation and end point level data extraction. Mammalian bioassay studies not meeting these criteria did not move forward and were summarized at the literature inventory level only.

Study evaluation was conducted using the EPA’s version of HAWC.^57^ Study evaluation is further described in the Supplemental file (under section “Study Evaluation and Data Extraction”). Briefly, for each study evaluation domain, at least two reviewers reached a consensus rating of “Good,” “Adequate,” “Deficient,” “Not Reported,” or “Critically Deficient.” After a consensus was reached, the reviewers considered the identified strengths and limitations to reach an overall study confidence rating of “High,” “Medium,” “Low,” or “Uninformative.” For epidemiology studies, the top two rating levels (“Good” or “Adequate” and “High” or “Medium” confidence) were combined because they have originally been extracted for a different EPA assessment or SEM project. Because evaluation decisions may have been different across these projects due to evaluation criteria, reviewer variability, and project goals specific to the parent project, merging the top two ratings allows for improved consistency across studies within this project. Key study evaluation considerations included potential sources of bias (factors affecting the magnitude or direction of an effect in a systematic way) and insensitivity (factors limiting detection of a true effect). Core and prompting questions used to guide the judgment for each domain are described in more detail in the IRIS Handbook^36^ and in the standard template language used for SEMs developed by EPA ORD^37^ and have only been adjusted, where appropriate, for the specific needs of this SEM.

As described in the Supplemental file (under section “Literature Evaluation and Data Extraction”), full results data extraction was conducted for prioritized mammalian bioassay studies by two members of the evaluation team. The team used the EPA’s version of HAWC to extract data, including basic study information, experiment details, animal group specifics, dosing regimen, end points evaluated, and results (qualitative or quantitative) by end point. Authors were not contacted for information not reported in a study. Data extraction was performed by one member of the evaluation team (primary extractor) and checked by a second member for completeness and accuracy (secondary extractor). Data extraction results were used to create HAWC visualizations (e.g., exposure–response arrays) by health system and effect for each of the PFAS. Although outside the scope of this SEM, once in HAWC, the extracted data can be used for evidence synthesis and benchmark dose modeling on an end point-by-end point basis at the discretion of the user.

For epidemiological studies, PECO-relevant studies after full-text review were extracted using a structured form in DistillerSR, as described in Radke et al.^23^ Information extracted included: citation information, study design characteristics, whether correlations across PFAS were presented, covariates used in statistical modeling, and quantitative results. Data extraction results are presented in the interactive Tableau dashboard^56^ and for download as an Excel file.

## Results

The study counts and figures presented in this manuscript represent a snapshot in time of a repository that may evolve as this project is updated or revisions are made. For the most current information, visit the web-based inventory and project pages in HAWC and HERO.^38,58^ The EPA anticipates releasing an update of the Comprehensive PFAS Dashboard to include additional PFAS as ongoing assessments are finalized. However, the EPA does not currently have plans to routinely update the SEMs moving forward. As priorities are identified and additional resources become available, the EPA may conduct updates, most likely targeted to certain PFAS when specific assessments are undertaken, building from the existing evidence maps.

### Literature Screening

The 2022 database searches yielded 152,205 records following duplicate removal (Figure 2). After application of the SWIFT-Review evidence stream filters for human, animal, and in vitro evidence, the total number of studies for consideration was reduced to 48,238. Following application of the artificial intelligence (AI) model for the literature prioritization step, the total number of references for screening in SWIFT-Active was reduced to 6,682. These references were then screened using predictive relevance in SWIFT-Active, and the total number of references considered potentially PECO-relevant or potentially relevant supplementary material was reduced to 3,012.

**Figure 2:**
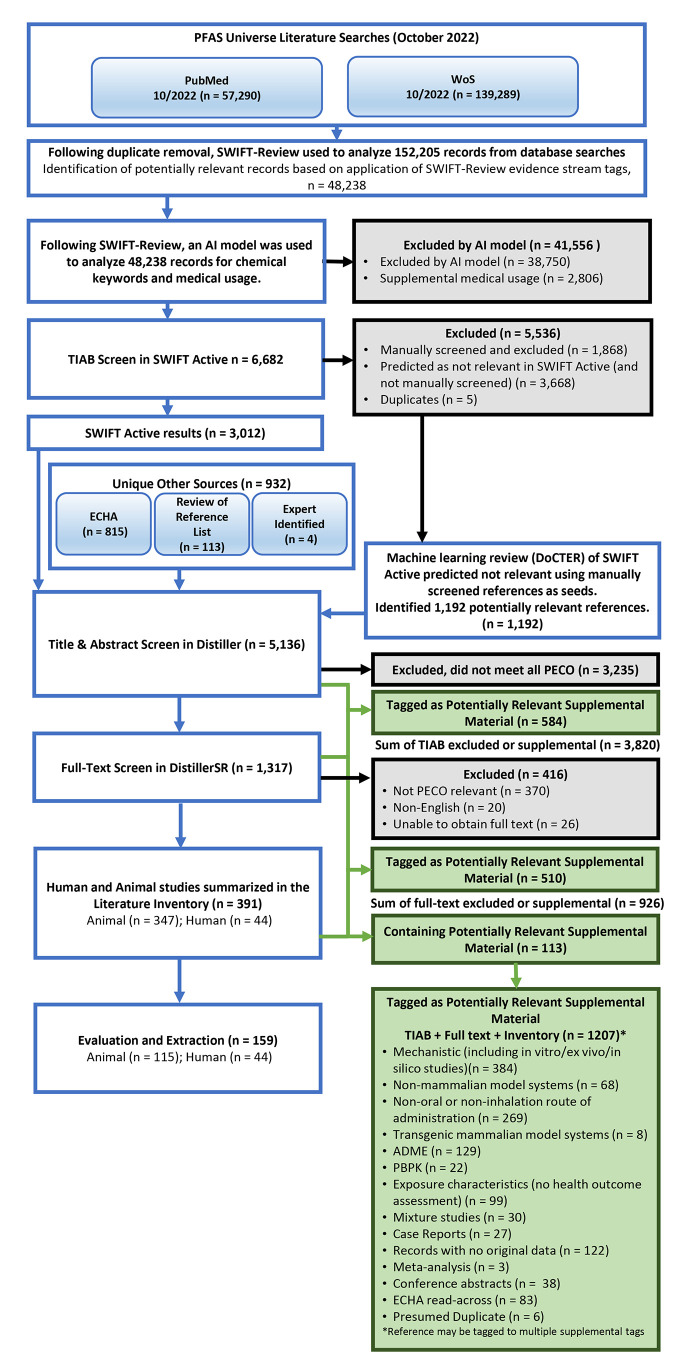
PFAS universe study flow diagram literature searches and results are pooled across years. Note: References identified from other sources joined screening at Distiller SR title-abstract review. Some references may have multiple potentially relevant supplementary material or exclusion tags.

An additional 932 unique studies were identified from the gray literature searched, including 113 that came from reviewing the reference lists of studies considered PECO relevant after full-text review. A final supervised machine learning predictive model in the DoCTER^55^ was applied to all unscreened studies that met the keyword-based exclusion rule detailed during the prioritization step. Studies manually screened as included were used as seeds in the predictive model and resulted in an additional 1,192 references identified for screening. The 1,192 references were imported into DistillerSR for a total of 5,136 studies screened at TIAB level. During TIAB screening in DistillerSR, 1,323 were included for full-text review, 584 were tagged as potentially relevant supplementary material, and 3,235 were excluded as not relevant to PECO.

During full-text review in DistillerSR, 391 studies were identified as PECO relevant, including 347 mammalian bioassay studies and 44 epidemiology studies, and 510 studies were tagged as potentially relevant supplementary material. There were 416 studies excluded for not being PECO relevant, lacking full-text availability, being duplicates, or being written in a non-English language. These studies were all summarized in the interactive literature inventories. Of the 391 PECO-relevant studies, 159 studies proceeded to study evaluation and extraction if they met the criteria. These 159 studies represented 74 PFAS. During literature inventory evaluation, an additional 113 studies were identified to contain potentially relevant supplementary material.

The literature screening/tagging is displayed visually in Figure 2 and Figure 3. Figure 3 is an interactive literature tag tree, which allows users to download lists of references by interacting with the visual by clicking on nodes. In addition, chemical-specific literature tag trees (filter by visualization type “literature tagtree”) are also available in HAWC (for 182 PFAS). A full export of the literature tagging export is available in HAWC and in Excel Table AS8.

**Figure 3:**
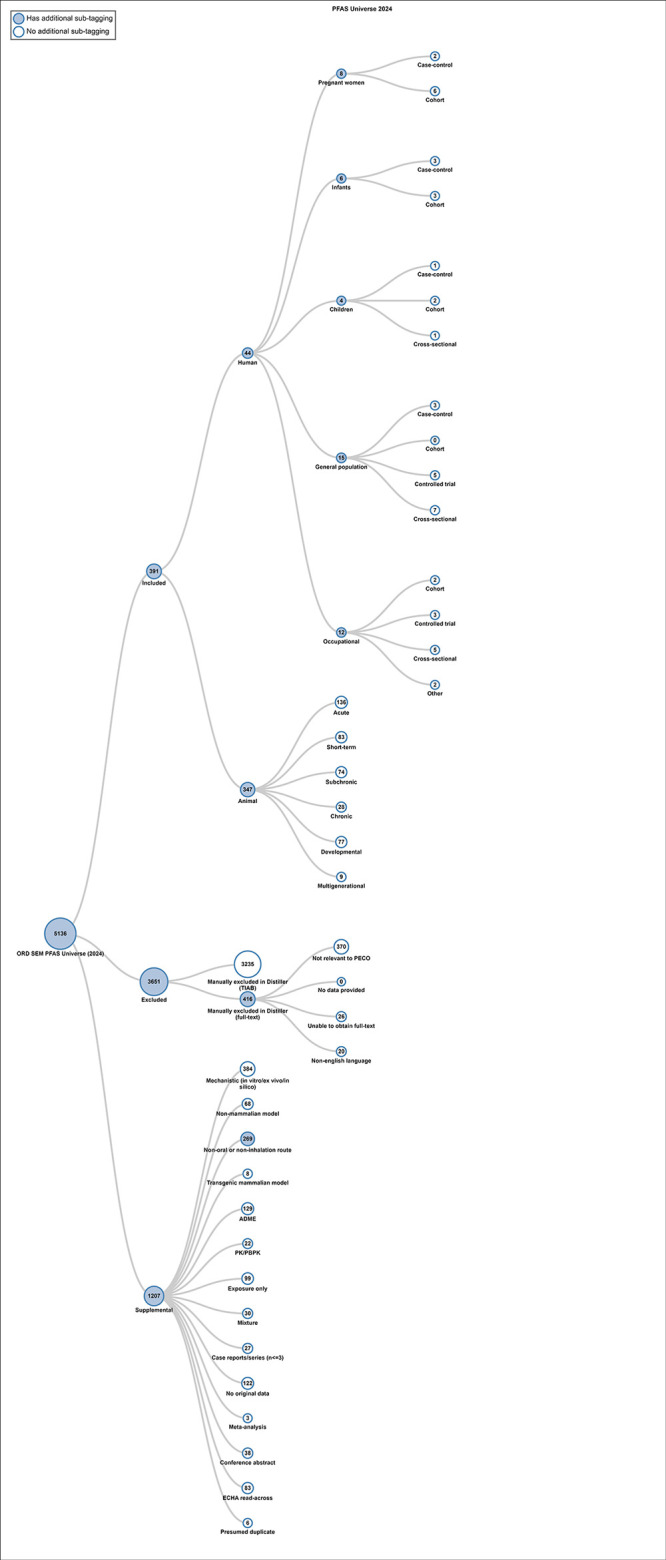
Literature inventory tree. Screenshot from interactive image. This is a thumbnail image of the interactive visual https://hawc.epa.gov/summary/visual/assessment/100500347/pfas-universe-2024/); accessed November 24, 2024.

### Details of Identified Mammalian Bioassay and Epidemiological Studies

#### Mammalian Bioassay Studies

##### Literature Inventory

A total of 347 mammalian bioassay studies were identified as meeting PECO criteria. Figure 4 provides an overview of the animal model systems, study designs, and health effects systems identified during literature inventory. Further details on these studies, including exposure route and chemicals studied, can be found in an interactive graphic in HAWC.^56^ PECO-relevant mammalian bioassay studies evaluated exposure to unique PFAS administered via oral (diet, water, or gavage) and inhalation routes of exposure. A range of species was tested, including rats, mice, guinea pigs, hamsters, rabbits, nonhuman primates, dogs, cattle, cats, and pigs. Most studies were conducted in rats and mice. The majority of studies identified were of acute, short-term, or subchronic duration, with substantially fewer studies of chronic duration or with developmental designs. Of these, bisphenol AF (*n* = 21), 1,1,1,2 tetrafluoroethane (*n* = 18), perfluoroisobutene (*n* = 17), tetrafluoroethylene (*n* = 16), and difluoromethane (*n* = 15) were the most frequently studied chemicals. For example, 72 PFAS had acute toxicity data available, but only 16 chemicals had chronic bioassay data available, and 39 chemicals had reproductive/developmental bioassay data available.

**Figure 4:**
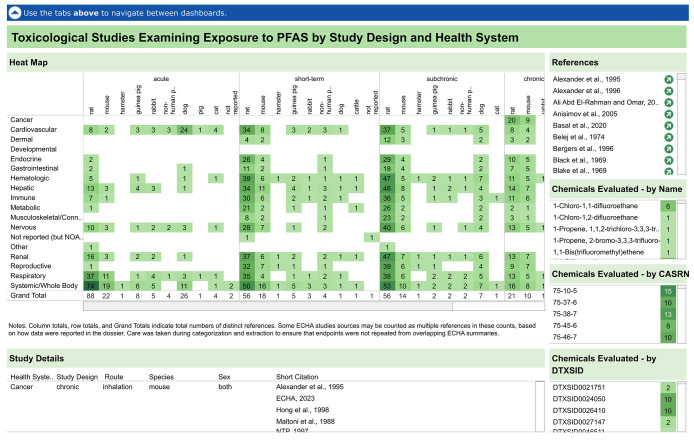
Survey of mammalian bioassay studies that met population, exposure, comparator, outcome (PECO) criteria by study design, species, and health systems. This is an image of the interactive visual (https://hawc.epa.gov/summary/visual/assessment/100500347/PFAS-Universe-Tableau-Dashboard/); accessed on March 6, 2025. It is filterable by health system, study design, PFAS name, Chemical Abstracts Service registry number (CASRN), and DSSTox substance identifier (DTXSID).^38,73^ The numbers in the heat map inset indicate the distinct number of studies that investigated a health system within a particular study design. If a study evaluated multiple health outcomes or presented several experiments, it is shown here multiple times, though totals reflect distinct numbers of studies. The study design panel includes information on animal model, exposure duration, route of administration, and dose level(s) tested.

The interactive dashboard (Animal Evidence tab) includes the design summary for each study (route, species, sex, health system). Drawing conclusions across health systems is outside the scope of this publication.

##### Study Evaluation

Of the 347 PECO-relevant mammalian studies, only studies containing a health outcome combined with an exposure duration of ≥21 days (or using a reproductive or developmental design) underwent study evaluation and subsequent data extraction because these study designs were considered most suitable for identifying a subchronic or chronic point of departure and subsequent toxicity value derivation. The 115 studies that matched these criteria assessed 60 individual PFAS. The detailed HAWC extractions for study evaluations are available for download in EPA HAWC and presented in Excel Table AS9. Figure 5 contains the study evaluation report for all non-ECHA mammalian bioassay studies, and Figure 6 contains the study evaluation report for ECHA mammalian bioassay studies. Specific rationales for each domain, as well as the overall confidence ratings, are available in an interactive graphic in HAWC. As noted in previous SEMs, many of the mammalian bioassay studies were identified from ECHA summaries (27/115). Many ECHA summaries received low confidence ratings due to limited study details and primary information for results (e.g., qualitative results reporting). Information on blinding and randomized allocation of animals to experimental groups was rarely reported across the mammalian toxicology studies, as reflected by “not reported” ratings and low scores across these domains. The hashing in visuals reflects outcome-specific judgments that varied within a study. Specific details regarding the outcome-specific judgments can be viewed by clicking on the cells within in the interactive web version of the HAWC visualization.^59,60^

**Figure 5:**
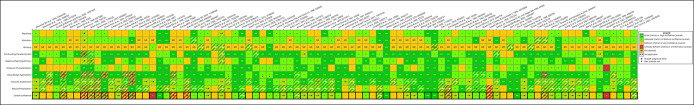
Study evaluation for non-ECHA mammalian bioassay studies. This is an image of the interactive visual (https://hawc.epa.gov/summary/visual/assessment/100500347/nonECHA_SQE/); accessed September 20, 2024. The study evaluation approach follows standard methods that are used in systematic evidence maps developed by the EPA and have only been adjusted, where appropriate, for the specific needs of this SEM. A full download of detailed evaluation summaries is available through HAWC, or in Excel Table AS9. Note: ECHA = European Chemicals Agency; HAWC = Health Assessment Workspace Collaborative; SEM = Systematic Evidence Map.

**Figure 6:**
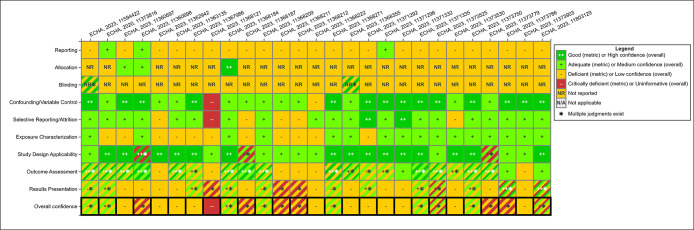
Study evaluation for ECHA mammalian bioassay reports. This is an image of the interactive visual (https://hawc.epa.gov/summary/visual/assessment/100500347/ECHA_SQE/ ) accessed September 20, 2024. The study evaluation approach follows standard methods that are used in systematic evidence maps developed by the EPA and have only been adjusted, where appropriate, for the specific needs of this SEM. A full download of detailed evaluation summaries is available through HAWC, or in Excel Table AS9. Note: ECHA = European Chemicals Agency; HAWC = Health Assessment Workspace Collaborative; SEM = Systematic Evidence Map.

##### Summary of Extracted Data

Data extraction was conducted for the 115 PECO-relevant studies with exposure durations ≥21 days or using reproductive or developmental design. The most commonly assessed health systems include whole body (98 studies; e.g., body weight, mortality); reproductive (84 studies; e.g., male and female reproductive organ weight, fertility); hepatic (75 studies; e.g., liver weight, liver enzymes, nonneoplastic lesions); metabolic (64 studies; e.g., lipids and lipoproteins, triglyceride lipase activity); renal/urinary (63 studies; e.g., kidney weight, kidney histopathology); endocrine (59 studies; e.g., weight and histopathology of the thyroid, adrenal gland, and pituitary gland); respiratory (59 studies; e.g., lung weight, nonneoplastic lesions); nervous system (58 studies; e.g., brain weight, behavioral measurements); immune (54 studies; e.g., immunologic factor, lymphocytes, weight and histopathology of the lymph node, thymus, spleen); hematologic (52 studies; e.g., red blood cell counts, serum biochemical measures like hemoglobin); cardiovascular (49 studies; e.g., heart weight, blood pressure); and developmental (46 studies; e.g., pup weight, skull and skeletal development) systems. Table S2 contains the categorization for end point groupings by health system.

Many of the mammalian bioassay studies available were identified from searches of the gray literature, which had more limited methods and results reporting, limiting their utility in assessment development. Figure 7, Figure 8, and Figure 9 are example visualizations of the data available for some of the PFAS mammalian bioassay studies. These visualizations highlight example chemical surveys of the available data, organized by health system or experimental design. More than 95 visuals presenting exposure–response arrays (data pivot visuals) on 60 PFAS across 16 health effect systems are available on the HAWC project page (visualizations, filter “data pivot”). We were unable to fully discuss them here because of space constraints. Table S4 also describes the inventory of HAWC visuals available by PFAS and health system. Figure 7 displays oral toxicity data for the chemical pyrifluquinazon. Figure 8 shows inhalation data for chemical 2,3,3,3-tetrafluoropropene. Figure 9 shows oral toxicity data for *N*-[2,5-dichloro-4-(1,1,2,3,3,3-hexafluoropropoxy)-phenyl-aminocarbonyl]-2,6-difluorobenzamide, which included reproductive and developmental toxicity data. For some chemicals, data are split across multiple HAWC visuals (for example, bisphenol AF, which has seven unique visuals describing the mammalian bioassay data). Excel Table AS10 contains a full summary of the mammalian bioassay data for all visualizations.

**Figure 7:**
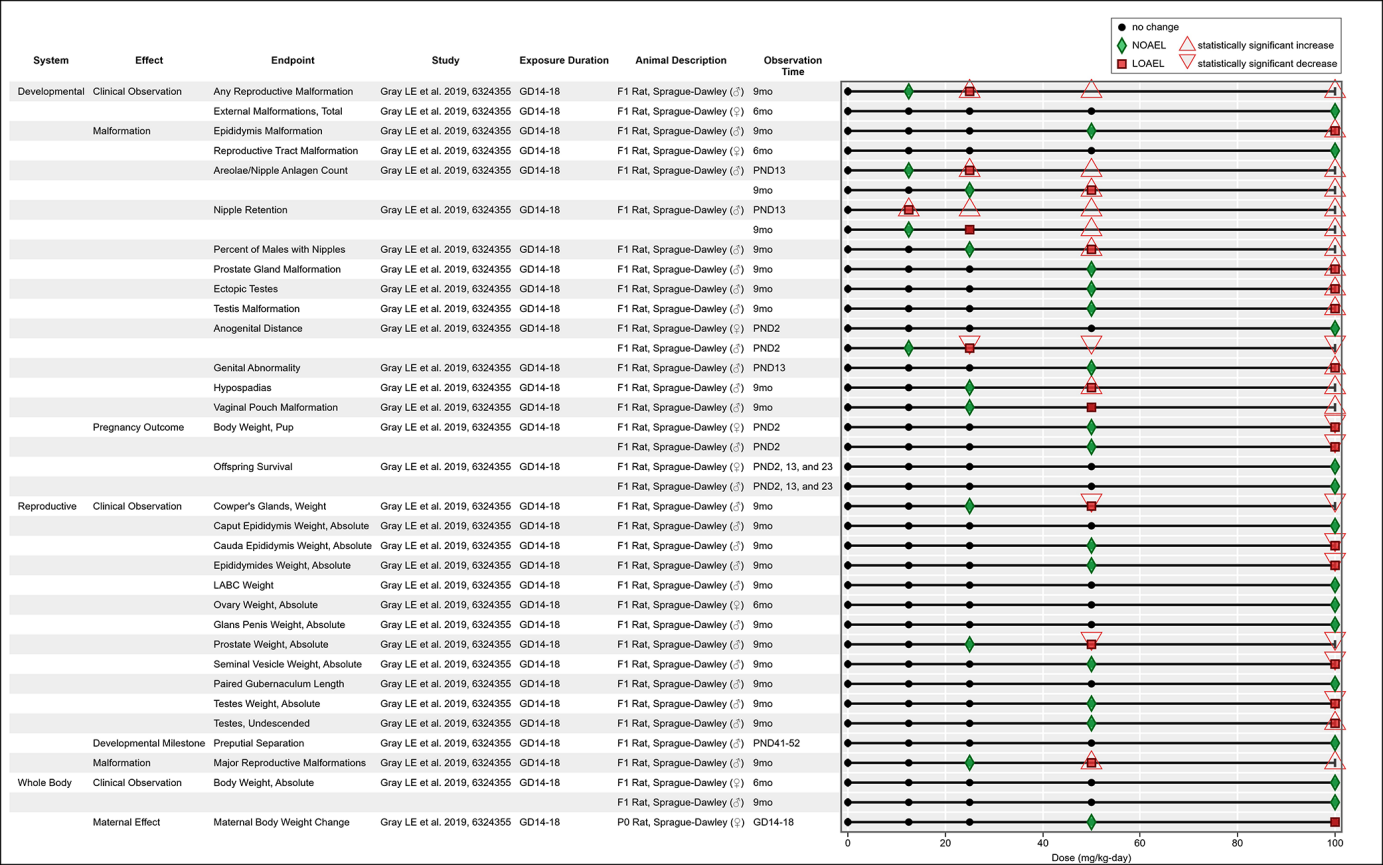
Survey of oral toxicology data for pyrifluquinazon example. This is an image of the interactive visual (https://hawc.epa.gov/summary/data-pivot/assessment/100500347/74-Figure-74-Pyrifluquinazon_Oral/); accessed September 19, 2024. Data are also available in Excel Table AS10 (filter by chemical name). The seven-digit number in “study” column is the Health and Environmental Research Online identification. Note: m = month, GD = gestation day, PND = postnatal day, LOEL = lowest-observed-effect level, NOEL = no-observed-effect level.

**Figure 8:**
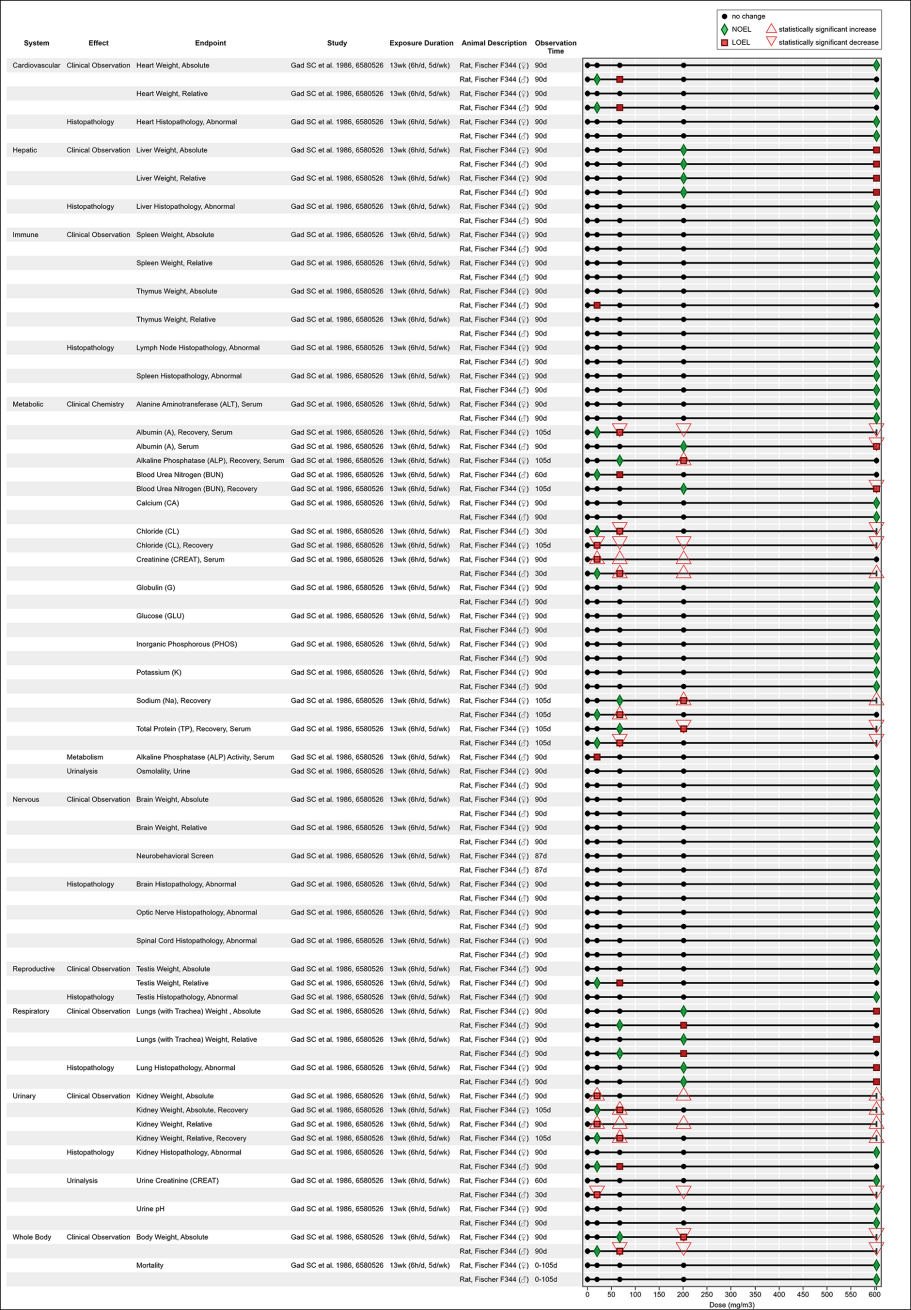
Survey of inhalation toxicology data for 1,1-bis­(trifluoromethyl)­ethene. This is an image of the interactive visual (https://hawc.epa.gov/summary/data-pivot/assessment/100500347/11-Figure-11-11-Bistrifluoromethylethene/) accessed 19 September, 2024. Data are also available in Excel Table AS10 (filter by chemical name). The seven-digit number in “study” column is the Health and Environmental Research Online identification. Note: d = days; m = month, GD = gestation day, PND = postnatal day, LOEL = lowest-observed-effect level, NOEL = no-observed-effect level.

**Figure 9:**
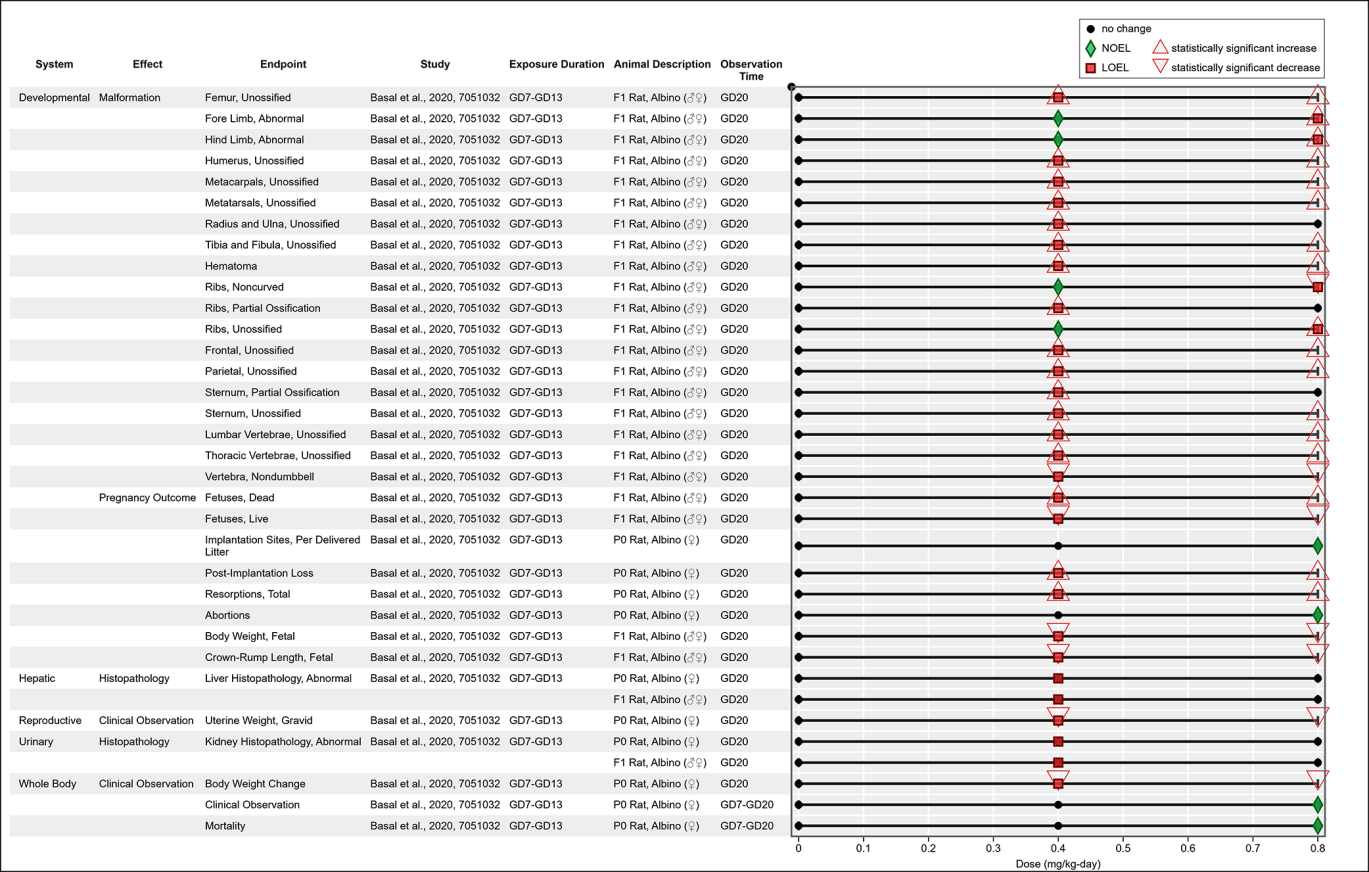
Survey of oral toxicology findings for *N*-[2,5-dichloro-4-(1,1,2,3,3,3-hexafluoropropoxy)-phenyl-aminocarbonyl]-2,6-difluorobenzamide. This is an image of the interactive visual (https://hawc.epa.gov/summary/data-pivot/assessment/100500347/65-Figure-65-N-4-111333-Hexafluoro-2-hydroxy--1dc6/) accessed on March 5, 2025. Data are also available in Excel Table AS10 (filter by chemical name). The eight-digit number in “study” column is the Health and Environmental Research Online identification. Note: d = days; m = month, GD = gestation day, PND = postnatal day, LOEL = lowest-observed-effect level, NOEL = no-observed-effect level.

#### Epidemiology Studies

##### Literature Inventory

A total of 44 epidemiology studies were identified as meeting PECO criteria. A summary of the study design, population, and health effects systems identified during literature inventory is provided in Figure 10. An interactive graphic in HAWC (in the Human Evidence tab) provides additional details including exposure measurements and chemicals studied.^56^ PECO-relevant epidemiological studies included information on 30 different PFAS, although the majority of these had only one human study available (Table 3). The most frequently studied PFAS with five or more studies were perfluoroundecanoate (*n* = 12), bisphenol AF (*n* = 6), and perfluoroheptanoate (*n* = 5).

**Figure 10:**
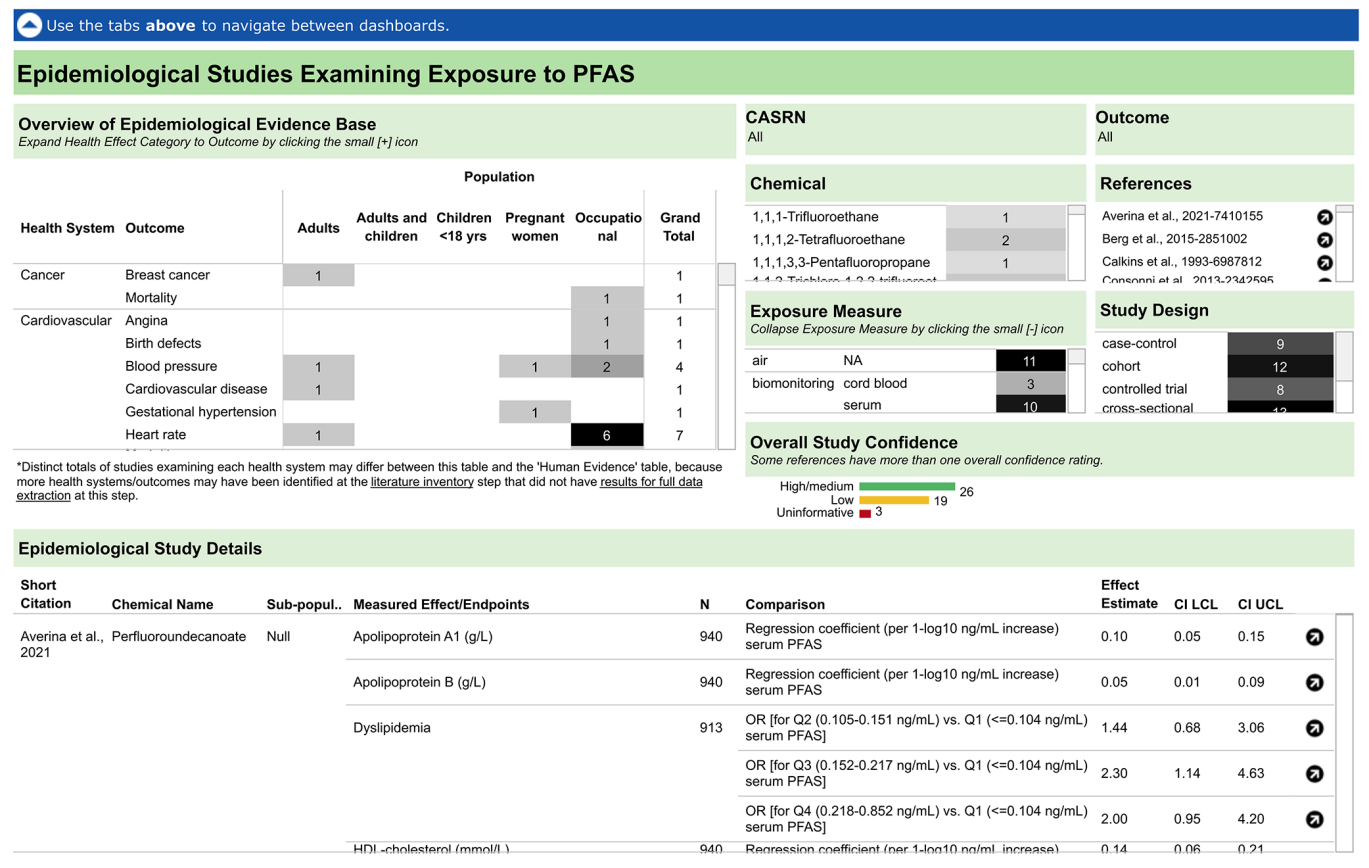
Overview of human studies that met population, exposure, comparator, outcome (PECO) criteria summarized by study design, population, exposure measure, health systems assessed, and study confidence information. This is a thumbnail image of the interactive visual accessed on March 6, 2025. The numbers indicate the distinct number of studies that investigated a health system within a particular study design and population, not the number of studies that observed an association with PFAS exposure. If a study evaluated multiple health outcomes or populations, it is shown here multiple times, although totals reflect distinct numbers of studies. Interactive sorting allows searching by name, Chemical Abstracts Service registry number (CASRN), and DSSTox substance identifier (DTXSID), exposure measurement information, and sex.

**Table 3:** PFAS Identified in This Systematic Evidence Map That Had at Least One Study Summarized in the Literature Inventory^a^,^c^

Chemical name	CASRN	Human evidence	Animal evidence^b^	Prioritized animal evidence^a^
(1*E*)-1-Chloro-3,3,3-trifluoro-1-propene	102687-65-0	—	6	2
(1*RS*,2*RS*)-2-(4-Hydroxyphenyl)-2-methyl-1-(9-(4,4,5,5,5-pentafluoropentyl)sulfinylnonyl)-1,2,3,4-tetrahydronaphth-6-ol	101908-22-9	—	1	—
(1*Z*)-1-Chloro-2,3,3,3-tetrafluoroprop-1-ene	111512-60-8	—	1	1
(2,2,2-Trifluoroethoxy)ethene	406-90-6	—	2	—
(*E*)-1,3,3,3-Tetrafluoro-1-propene	29118-24-9	—	13	1
(*Z*)-1,1,1,4,4,4-Hexafluoro-2-butene	692-49-9	—	4	2
1,1,1,2,3,3-Hexafluoropropane	431-63-0	—	1	1
1,1,1,2-Tetrafluoroethane	811-97-2	2	18	6
1,1,1,3,3,3-Hexafluoro-2-chloropropane	431-87-8	—	1	—
1,1,1,3,3,3-Hexafluoropropane	690-39-1	—	1	1
1,1,1,3,3-Pentafluoropropane	460-73-1	1	10	2
1,1,1,4,4,4-Hexafluorobut-2-ene	66711-86-2	—	5	—
1,1,1-Trichloro-2,2,2-trifluoroethane	354-58-5	—	1	—
1,1,1-Trifluoroethane	420-46-2	1	6	2
1,1,2,2-Tetrafluoroethane	359-35-3	—	1	1
1,1,2-Trichloro-1,2,2-trifluoroethane	76-13-1	2	6	4
1,1-Bis(trifluoromethyl)ethene	382-10-5	—	1	1
1,1-Difluoroethane	75-37-6	—	10	1
1,2-Dichloro-1,1,2,2-tetrafluoroethane	76-14-2	—	3	1
1,2-Difluoroethane	624-72-6	—	1	—
1,3-Dichloro-1,1,2,2,3-pentafluoropropane	507-55-1	—	2	2
1-Chloro-1,1-difluoroethane	75-68-3	—	6	1
1-Chloro-1,2-difluoroethane	338-64-7	—	1	—
1-Propene, 1,1,2-trichloro-3,3,3-trifluoro-	431-52-7	—	1	1
1-Propene, 2-bromo-3,3,3-trifluoro-	1514-82-5	—	1	—
2-(*N*-Methylperfluorobutanesulfonamido)ethyl methacrylate	67584-59-2	—	2	—
2-(*N*-Methylperfluorobutylsulfonamido)ethyl acrylate	67584-55-8	—	3	2
2-(Perfluorodecyl)ethanol	865-86-1	1	—	—
2,2,2-Trifluoroethanol	75-89-8	—	2	—
2,2-Bis(trifluoromethyl)-1,3-dioxolane	1765-26-0	—	1	—
2,2-Dichloro-1,1,1-trifluoroethane	306-83-2	3	14	9
2,3,3,3-Tetrafluoro-2-(trifluoromethyl)propanenitrile	42532-60-5	—	2	—
2,3,3,3-Tetrafluoropropene	754-12-1	—	9	1
2,4,6-Trimethyl-2,4,6-tris(3,3,3-trifluoropropyl)cyclotrisiloxane	2374-14-3	—	2	1
2-{[(Bicyclo[2.2.1]hept-5-en-2-yl)oxy]methyl}-1,1,1,3,3,3-hexafluoropropan-2-ol	305815-63-8	—	1	1
2-Bromo-1,1,1,2-tetrafluoroethane	124-72-1	—	1	—
2-Bromo-1,1,1-trifluoroethane	421-06-7	—	1	—
2H-Perfluoropropane	431-89-0	1	5	—
2-Propyl-4-pentafluoroethyl-1-((2’-(1H-tetrazol-5-yl)biphenyl-4-yl)methyl)imidazole-5-carboxylic acid	124750-95-4	—	1	—
3,3-Dichloro-1,1,1,2,2-pentafluoropropane	422-56-0	—	2	2
3,4,5-Trifluorophenol	99627-05-1	—	1	1
3-Trifluoromethylpyridine	3796-23-4	—	2	1
4-(Perfluoro(2,2-bis(1-methylethyl)(1-methyl)ethen-1-yloxy))benzenesulfonic acid sodium salt	70829-87-7	—	3	3
4,5-Difluoro-2,2-bis(trifluoromethyl)-1,3-dioxole	37697-64-6	—	1	—
4:2 Fluorotelomer sulfonate	414911-30-1	1	—	—
5-(1,2,2,2-Tetrafluoro)ethoxy-perfluoro-3-oxa-4-methylpentanesulfonic acid	749836-20-2	2	2	2
6:2 Fluorotelomer sulfonate sodium salt	27619-94-9	1	—	
6:2 Fluorotelomer thiohydroxy ammonium chloride	88992-45-4	—	1	—
Ammonium 2-(2-(2-(aminosulphonyl)-1,1,2,2-tetrafluoroethoxy)-1,1,2,3,3,3-hexafluoropropoxy)-2,3,3,3-tetrafluoropropionate	4089-61-6	—	1	—
Bisphenol AF	1478-61-1	6	21	17
Bromodifluoromethane	1511-62-2	—	1	—
Bromotrifluoromethane	75-63-8	2	7	—
Carbon tetrafluoride	75-73-0	—	1	—
Carbonic difluoride	353-50-4	—	1	—
Chlorodifluoromethane	75-45-6	4	8	5
Chloropentafluorobenzene	344-07-0	—	3	2
Chloropentafluoroethane	76-15-3	—	1	—
Chlorotrifluoroethylene	79-38-9	—	10	1
Dibromodifluoromethane	75-61-6	—	1	—
Dichlorodifluoromethane	75-71-8	2	12	4
Difluoromethane	75-10-5	—	15	4
Ethene, 1,1-dichloro-2,2-difluoro-	79-35-6	—	2	—
Ethene, trifluoro-	359-11-5	—	1	—
Flubendiamide	272451-65-7	—	1	1
Hexafluoroacetone	684-16-2	—	1	1
Hexafluorobenzene	392-56-3	—	2	—
Hexafluoropropene	116-15-4	—	10	—
Methane, bromochlorodifluoro-	353-59-3	1	2	1
Methyl 2,2,3-trifluoro-3-oxopropanoate	69116-71-8	—	1	—
*N*-(4-(1,1,1,3,3,3-Hexafluoro-2-hydroxy-propan-2-yl)phenyl)-N-(2,2,2-trifluoroethyl)benzenesulfonamide	293754-55-9	—	8	3
*N*,*N*-Bis(2-hydroxyethyl)perfluorobutanesulfonamide	34455-00-0	—	2	—
*N*-[2,5-dichloro-4-(1,1,2,3,3,3-hexafluoropropoxy)-phenyl-aminocarbonyl]-2,6-difluorobenzamide	103055-07-8	—	5	4
*N*-Nitroso(2,2,2-trifluoroethyl) ethylamine	82018-90-4	—	1	1
*N*-Nitrosobis(4,4,4-trifluoro-*N*-butyl)amine	83335-32-4	—	1	1
Octafluorocyclobutane	115-25-3	—	4	—
Pentafluoroethane	354-33-6	—	11	1
Pentafluoroiodoethane	354-64-3	—	1	—
Perflunafene	306-94-5	—	1	—
Perfluoro-2-methoxyacetic acid	674-13-5	1	1	1
Perfluoro-3,5,7,9,11-pentaoxadodecanoic acid	39492-91-6	2	1	1
Perfluoro-3,5,7,9-butaoxadecanoic acid	39492-90-5	2	2	2
Perfluoro-3,5,7-trioxaoctanoic acid	39492-89-2	—	1	1
Perfluoro-3,5-dioxahexanoic acid	39492-88-1	—	1	1
Perfluorododecanoate	171978-95-3	3	—	—
Perfluoroethane	76-16-4	—	6	2
Perfluoroethyl vinyl ether	10493-43-3	—	2	1
Perfluoroheptanoate	120885-29-2	5	—	—
Perfluorohexane	355-42-0	—	1	—
Perfluorohexyl phosphonic acid	40143-76-8	1	—	—
Perfluoroisobutene	382-21-8	—	17	—
Perfluorooctadecanoic acid	16517-11-6	—	1	1
Perfluoropentanoate	45167-47-3	1	—	—
Perfluorotetradecanoate	365971-87-5	4	—	—
Perfluorotridecanoate	862374-87-6	3	—	—
Perfluoroundecanoate	196859-54-8	12	—	—
Perflutren	76-19-7	—	1	—
Potassium 11-chloroeicosafluoro-3-oxaundecane-1-sulfonate	83329-89-9	1	—	—
Potassium 9-chlorohexadecafluoro-3-oxanonane-1-sulfonate	73606-19-6	2	2	2
Pyrifluquinazon	337458-27-2	—	1	1
*S*-(1,1,2,2-Tetrafluoroethyl)cysteine	94840-66-1	—	1	—
Sodium perfluoroheptane sulfonate	21934-50-9	3	—	—
Sodium trifluoroacetate	2923-18-4	—	3	1
Tetrafluoroethylene	116-14-3	1	16	1
Tetrafluoro-*m*-phenylenediamine	1198-63-6	—	1	1
Tiflamizole	62894-89-7	—	1	—
Trifluoro(trifluoromethyl)oxirane	428-59-1	—	7	3
Trifluoroacetyl chloride	354-32-5	—	2	1
Trifluoroiodomethane	2314-97-8	—	7	4
Trifluoromethane	75-46-7	1	10	2
Trifluoromethanesulfonic anhydride	358-23-6	—	1	—
Vinylidene fluoride	75-38-7	—	13	4

a Study counts are listed by animal and human evidence.

b An interactive visual summary of the literature inventory of these chemicals can be found in Tableau^56^ and the ORD SEM PFAS Universe HAWC project.^38^

c Prioritized animal evidence refers to animal studies containing ≥21 days exposure or assessing reproductive/developmental end points.

d —: indicates that no studies were identified. CASRN: Chemical Abstracts Service registry number. HAWC: Health Assessment Workplace Collaborative. ORD: EPA Office of Research and Development. PFAS: Per- and polyfluoroalkyl substances. SEM: Systematic Evidence Map.

##### Study Evaluation

An overview of the overall study confidence by health system is provided in Figure 11 and by study in the interactive graphic in HAWC.^56^ The assessments are outcome specific, which sometimes can result in more than one rating for studies assessing multiple outcomes with different ratings. Over half of the studies (*n* = 26) were considered *medium/high* confidence for at least one outcome. Fifteen studies were *low* confidence and three were *uninformative* for all outcomes. Unlike the previous SEMs,^22,25^ over a quarter of the studies identified in this SEM were of occupational groups (*n* = 12) and the majority (*n* = 10) of them had *low* or *uninformative* confidence for at least one outcome. Details on study evaluation determinations and rationales are available in the interactive dashboard (Human Study Confidence Summary tab) which is filterable by chemical, health system and outcome, study evaluation domain, and rating.^56^ In addition, the study quality evaluation summary for epidemiology studies is available in Excel Table AS11.

**Figure 11:**
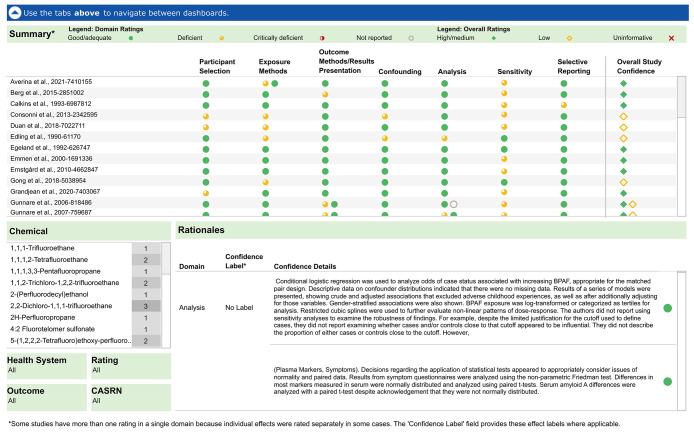
Summary of study evaluations for epidemiological studies by health effect category. This is a thumbnail image of the interactive visual accessed in the interactive dashboard (“Study Confidence Summary” tab, each domain/overall confidence rating and rationale is available, filterable by PFAS, health effect category, outcome, and rating.) Note: The study evaluation approach follows standard methods that are used in systematic evidence maps developed by the EPA^37,77^ and have only been adjusted, where appropriate, for the specific needs of this SEM. A full download of detailed evaluation summaries is available in Excel Table AS11.

Most studies received good/adequate ratings in many domains. The sensitivity domain was considered deficient in 36 (86%) of studies for at least one PFAS and in 32 (73%) for all PFAS. The main concern was limited exposure contrasts, which likely hampered the ability of the studies to detect true effects. The exposure levels are presented in the interactive dashboard.^56^ However, null findings due to low study sensitivity cannot be interpreted as absence of adverse health effects.

##### Summary of Extracted Data

The most studied epidemiology health systems in this PFAS universe SEM were cardiovascular (*n* = 15 studies), metabolic (*n* = 11), nervous (*n* = 9), and respiratory (*n* = 8). Other health systems with at least five studies included hematologic, hepatic, immune, renal, and developmental. Study designs included cross-sectional (*n* = 13 studies), cohort (*n* = 12), and case-control (*n* = 9). Also, unlike in previous SEMs,^22,25^ there were more controlled trials (*n* = 8) as well as other study designs, such as an occupational case-series^61^ and short-term within-subject comparisons.^62^ Most of the controlled trials administered industrial exposures (e.g., fire suppressants, refrigerants) to volunteer subjects. In 27 studies, PFAS exposures were measured using biomarkers. Blood was the most common biomatrix (*n* = 21), of which five studies measured maternal blood and three studies measured cord blood. Other biomatrices included urine (*n* = 4), breastmilk (*n* = 2), and semen (*n* = 1). PFAS exposures were also measured in air (*n* = 11), based on occupation (*n* = 3), and directly administered through inhalation (*n* = 3). In one study, PFAS exposure was related to an accidental discharge of bromotrifluoromethane.^63^ In addition to the three studies that determined exposures based on occupation, over half (*n* = 7) of the studies measuring PFAS exposures in air were studies of occupational groups. All controlled trials (*n* = 8) measured PFAS exposures in air or through direct administration by inhalation. Most studies (*n* = 24) were of the general population, including 14 studies in adults, seven in children (≤18 years old), and three in adults and children. Other study populations included occupational groups (*n* = 12) and pregnant women (*n* = 8).

In 15 studies of cardiovascular effects, the most common outcomes considered were changes in heart rate (*n* = 7), serum lipid levels (*n* = 6), and blood pressure (*n* = 4); 18 PFAS were examined with cardiovascular outcomes. PFAS exposures were based on biomarkers (*n* = 5), air (*n* = 4), occupation (*n* = 3), direct administration through inhalation (*n* = 2), and presence during an accidental discharge (*n* = 1). In 11 studies of metabolic effects, the primary outcomes included insulin resistance (*n* = 6) and gestational diabetes (*n* = 4); 16 PFAS were examined with metabolic outcomes. In nine studies of nervous system effects, motor (*n* = 5), cognition (*n* = 4), dizziness (*n* = 3), and pain (*n* = 2) were examined in more than one study; eight PFAS were considered with nervous system outcomes. In eight studies of respiratory outcomes, only lung function (*n* = 3) and dyspnea (*n* = 2) were considered in more than one study; seven PFAS were examined with respiratory outcomes. Immune system outcomes in seven studies primarily included inflammatory markers (e.g., C-reactive protein, serum amyloid A protein, fibrinogen) (*n* = 4), infectious diseases (*n* = 2), and autoimmune diseases (*n* = 1). Immune suppression as measured by reduced vaccine response was not evaluated in any study; eight PFAS were examined with immune system outcomes.

The interactive dashboard (the Epi Overview tab) includes the quantitative results for each study.^56^ In addition, the full download of data is available in Excel Table AS11. Drawing conclusions across health systems is outside the scope of this publication. As noted in our previous SEMs, here we evaluate only the critical effects that supported the 2024 EPA Final PFAS National Primary Drinking Water Regulations Rulemaking.^12,13^ The utility of these studies for toxicity value derivation depends on several factors, including the study confidence, consistency of the results across available studies, the type of quantitative results presented, and the likelihood of confounding by coexposures. Determining whether the evidence is sufficient for a given PFAS/outcome combination was not a goal of this evidence mapping project.

### Updates to the Comprehensive PFAS Dashboard

Data from this SEM were incorporated into the Comprehensive PFAS Dashboard described in Shirke et al.^25^ In brief, the Comprehensive PFAS Dashboard seeks to pool data across diverse EPA research and assessment efforts (“parent project”) to visualize data more readily across the PFAS chemical landscape. In addition, the Comprehensive PFAS Dashboard was updated to reflect literature inventory-level data extractions from the draft IRIS Toxicological Review on PFNA,^64^ the finalized IRIS Toxicological Review on PFDA,^65^ and the finalized human health toxicity assessments for PFOA^66^ and PFOS.^67^ Please note that the data extractions included in the Comprehensive PFAS Dashboard vary depending on the specific scope of the chemical’s parent project. Users should refer to each parent project for details about literature search strategies, scoping criteria, and data extraction methods. The Comprehensive Dashboard includes data for 14,760 PFAS, of which 25 chemicals are unknown or of variable composition, complex reaction products, or biological material (UVCB) compounds and thus are not represented in the “PFASSTRUCTv5” list.

At the time of publication of this manuscript, there are 714 mammalian bioassays and 657 epidemiology studies summarized in the Comprehensive PFAS Dashboard (which includes multiple EPA assessment and evidence map projects).^68^ In the Comprehensive PFAS Dashboard, the most frequently studied health effects systems in mammalian bioassays were systemic/whole body (*n* = 541), reproductive (*n* = 314), and hepatic (*n* = 313). Mammalian bioassays with exposure durations most useful for derivation of a subchronic or chronic reference dose (RfD) make up 48% (346/714) of the summarized literature (developmental/reproductive/multigenerational (*n* = 196), subchronic (*n* = 107), chronic (*n* = 43)). For epidemiology studies, the most frequently studied health effects systems were endocrine (*n* = 142), cardiovascular (*n* = 137), reproductive (*n* = 129), developmental (*n* = 117), and metabolic (*n* = 113). Most epidemiology studies used cohort (*n* = 331) or cross-sectional (*n* = 316) study designs.^68^

## Discussion

Many communities in the United States are experiencing contamination from PFAS. To gain a better understanding of potential human health risks from PFAS exposures, information is needed about the health effect data that currently exist from human and animal studies. The ORD SEMs represent an important first step in identifying and cataloging available health information about the thousands of PFAS across this chemical class. The use of publicly available, web-based, and interactive visualizations allows users to rapidly identify what types of health effect information are available for a given PFAS, and to better understand where there are data gaps across the group of compounds. For example, there is no chronic toxicity data for most PFAS.

The objective of building these evidence maps was to use systematic review methods to identify and collate the evidence base for all PFAS that had human health hazard data available. Because study evaluation and data extraction were performed for some PFAS, the evidence maps can also serve as a starting point for future systematic reviews. A full inventory of available visualizations by PFAS is available in Table S4. While no evidence synthesis was performed, SEMs are useful tools for assessment scoping and problem formulation activities. The existing ORD PFAS SEMs have been used to support the derivation of ORD human health toxicity values for several PFAS, including perfluoropropanoic acid^69^ and 6:2 fluorotelemer sulfonic acid.^70^ In addition, health effects information from our evidence maps were used by the EPA’s Office of Chemical Safety and Pollution Prevention to inform a proposed rulemaking to add over 15 PFAS categories representing more than 100 PFAS to the Toxics Release Inventory. This proposed rule, once finalized, will allow EPA to better understand how PFAS are being used and managed and provide more robust exposure information for use in future risk assessments. Furthermore, the SEMs have supported internal EPA analyses as well as state and other federal agency requests about data availability and research prioritization.

Completing this research effort in a timely and cost-effective manner was only possible because of the novel methods utilized for literature searching and screening. By using automated scripts to query the large number of PFAS iteratively, it allowed us to collate the literature searches for thousands of chemicals in one automated run. The process of identifying and documenting literature search results represents an important and labor-intensive step in most systematic reviews, and innovations to automate this process can reduce manual labor and save time. The typical time taken to perform a literature search varies from hours to weeks, depending on the complexity of the search involved. Because this survey involved nearly 15,000 chemicals, using an automated query script to remove chemicals without relevant articles before human manual review was the only feasible approach, allowing a team with limited resources to achieve what would otherwise be unachievable.

In addition to the search methods, this SEM also leveraged innovative algorithm advancements to aid in literature prioritization. Natural language processing (NLP) algorithms that classify documents into categories (such as relevant/not relevant) use an annotated training data set (a small subset of documents that have previously been categorized by subject matter experts) to build a predictive model. By reusing and refining sophisticated pretrained matrix representations of text, the BERT language model^50^ revolutionized the otherwise computationally intensive and time-consuming feature extraction process and reported best-in-class performance on document classification tasks compared with other feature extraction algorithms. This algorithm worked by generating context-aware feature representations based on processing vast volumes of text, which were then open-sourced to users who could efficiently fine-tune the feature representations to reflect the specific document corpus being classified in any context. The bioBERT large language model^51^ is one of a set of topic-focused language models based on BERT that further improved feature extraction on specific topics; the bioBERT large language model was designed for use in the context of scientific and biological texts by increasing the proportion of biological text used to generate the pretrained language model. The bioBERT model was reported as showing superior performance to the BERT model in document corpuses and text classification tasks relating to the biological sciences. For these reasons, we concluded that the bioBERT model would be most suitable for our purpose. Using these models allowed us to prioritize the references identified for screening and increase the likelihood of identifying the most relevant literature within our labor and cost constraints.

Table 3 contains a summary of identified mammalian and epidemiology studies listed by chemical that were identified for this SEM and summarized in literature inventory. These data are summarized across multiple interactive literature inventory dashboards and over 150 data pivot visualizations, available through HAWC.^71–73^ The visualizations can be viewed by PFAS, or by health system.

A total of 30 PFAS included in this SEM were identified in the epidemiology literature with the majority (*n* = 27 PFAS) having fewer than five studies. If we look across the ORD SEMs,^22–25^ only 65 PFAS had any human data available. Very few epidemiology studies have done comprehensive PFAS biomarker evaluations. For example, the Centers for Disease Control and Agency for Toxic Substances and Disease Registry have published exposure assessment reports across 10 sites nationwide which evaluated seven PFAS in blood, 14 PFAS in urine, 18 in water, and 33 in dust.^74^ The National Health and Nutritional Examination Survey (NHANES) surveys for roughly 20 PFAS (https://www.cdc.gov/exposurereport/data_tables.html). The state of Wisconsin has conducted biomonitoring in serum and measured 38 PFAS,^75^ which represents one of the largest concurrent PFAS evaluations for human exposure. Analytical challenges (lack of access to internal standards, diverse chemistries that complicate extraction/clean up steps) make it difficult to simultaneously analyze samples for broad suites of organofluorine compounds. Additional research is needed to fully characterize human exposures to PFAS by evaluating larger numbers of PFAS in biological media.

Only 111 PFAS of the thousands searched for this PFAS universe SEM had at least one publicly available mammalian or epidemiology study identified. Looking across the ORD SEMs,^22–25^ we have identified that only 214 PFAS have any available animal or epidemiology studies, highlighting that there are still unknowns regarding the human health effects of many PFAS. It is possible that other PFAS toxicity studies exist that are not publicly available due to confidential business information or other restrictions on release of data sets generated for commercial registrations. Because only ∼1% of searched PFAS had any mammalian bioassay or epidemiological evidence, more research and approaches (i.e., grouping, read-across) are needed to better characterize the potential toxicity of PFAS. The PFAS class exhibits a diversity of structural features, including fluorination status, functional groups, molecular size, and reactivity. Therefore, predictive tools are needed to fill data gaps, as there are so many unique structures without human health data available that cannot all be tested individually in a timely fashion.

Most of the PFAS assessed in this SEM were data poor, with thousands yielding no literature search results related to human health hazard information, emphasizing the need for robust toxicological and epidemiological information that can inform human health risk assessments of PFAS. This SEM, along with published results from our previous SEMs, provides researchers and regulators with a snapshot of the current PFAS human health evidence landscape, as well as a foundation for future systematic reviews. In addition, we hope this evidence map will inform future research and targeted testing to fill data gaps across the diverse PFAS space. While the focus of this SEM was on oral and inhalation exposures, Excel Table AS12 contains a summary of the identified studies using alternate exposure routes (i.e., injection, dermal, etc.).

Looking toward the future, one aspiration for this work is that it will facilitate conversations across the regulatory community about data reuse. Many U.S. and international health agencies have ongoing PFAS risk assessment work, and we expect that the scoping efforts (e.g., the literature identification and evaluation/extractions) conducted as part of the evidence map development might prove useful to other researchers and regulators. To this end, our previous PFAS SEM work^22^ was used as a draft case example for data reuse in preparation for an Organization for Economic Co-operation and Development (OECD) guidance document on use of research data in regulatory assessments (expected release in 2025).^76^ Keeping the databases current is a major challenge given the lag between the publication of new articles and the lengthy process of searching, screening, tagging, evaluating, and extracting relevant studies. Newer generation systematic review tools may help automate and reduce the level of manual labor required to conduct literature reviews on large databases, resulting in more up-to-date PFAS data repositories in the future.

## Supplementary Material





## Notes

Steps for how to run the PFAS study pre-prioritization code, systematic evidence map methods, and tables of PFAS assessment by U.S. EPA, categorization under health outcomes, and inventory of HAWC visuals (DOCX)
(ZIP)
