# Supplementary material for: Systematic Evidence Map for the Per- and Polyfluoroalkyl Substances (PFAS) Universe
Source: Environ Health Perspect. 2026 Apr 19;134(3):252–73. doi: 10.1021/EHP.6c00127 (PMC13347649; doi:10.1021/EHP.6c00127)
Supplement: Supplementary file 1 [file hp6c00127_si_001.docx]

# Supplemental File for “Systematic Evidence Map for the Per- and Polyfluoroalkyl Substances (PFAS) Universe”

**How to run the PFAS study pre-prioritization code**

**Step 1: Download the training, validation, and unclassified datasets, and master keyword lists dataset to a suitable directory**

- PFAS_15K_Training.csv
- PFAS_15K_Validation.csv
  - Note: the validation dataset has only 7/100 PECO relevant studies which will necessitate an adjustment downstream.
- PFAS_15K_Unclassified.csv
- PFAS 15K Master List _Revised 2023-05-16.xlsx

**Step 2: Run iPython notebook “PFAS_ML_Model.ipynb”**

- Note: this notebook was developed for use in a Google Cloud / Colab Pro environment with a GPU processor. Users wishing to work in an alternative environment may need to make a few changes to the notebook to reflect their method of accessing the GPU and required packages, although the core logic should be retained.
- In the final code block, update the directories for the input data directory, output data directory, and model save directory.
- If you have modified the training and validation files, update other required fields like the index of the text and the field name of the target columns.
- Download and store the output predictions files:
  - PFAS_15K_Unclassified_Relevance_wPredictions.csv
  - PFAS_15K_Unclassified_MedSup_wPredictions.csv
  - PFAS_15K_Validation_Relevance_wPredictions.csv

**Step 3: Combine AI predictions output from the PECO relevance and Medical/Supplementary output files**

- In Excel, or using a script, perform a left join such that the AI predictions for PECO relevance (PFAS_15K_Unclassified_Relevance_wPredictions.csv) are joined to the AI predictions for Medical/Supplementary information (PFAS_15K_Unclassified_MedSup_wPredictions.csv) using HERO_ID as a primary key.
- Rename the two Prob fields from the PECO relevance predictions and Medical/Supplementary predictions, respectively, as:
  - Prob_Relevance
  - Prob_MedSup
- Rename the two Pred_Calibrated fields from the PECO relevance predictions and Medical/Supplementary predictions, respectively, as:
  - Pred_Calibrated_Relevance
  - Pred_Calibrated_MedSup
- Save the combined predictions file as, for example, “PFAS_15K_Unclassified_Relevance_MedSup_Predictions.csv”

**Step 4: Adjust probability cutoff for PECO relevance prediction using the R script “PFAS_post-AI_prob_threshold_cubic_spline.R”**

- - Note: As noted above, there were only 7/100 PECO-relevant studies in the validation dataset. As a result, the automated method of determining the probability threshold at which the 95% recall target is achieved is too conservative. To make it more precise, we used cubic spline interpolation of the seven predicted probabilities of the relevant studies in the validation dataset using an R script.
  - To run the R script “PFAS_post-AI_prob_threshold_cubic_spline.R”,
    - sort the “PFAS_15K_Validation_Relevance_wPredictions.csv” output file generated in Step1 in descending order on the Prob field
    - filter to show only “Relevance” field = 1 (seven records)
    - hardcode the predicted probabilities of the seven records into the R script line 6. For example:
      - y <- c(0.95414984, 0.93122756, 0.08022824, 0.03894059, 0.01685493, 0.010813103, 0.00797064) # this represents the model predicted probabilities of PECO relevance of these seven studies in descending order
    - run the script
    - note the interpolated probability threshold for 95% recall (it turns out to be 0.011)
  - In the combined predictions file created in Step 3 (PFAS_15K_Unclassified_Relevance_MedSup_Predictions.csv), recalibrate the Pred_Calibrated_Relevance field such that its value is 1 above the threshold and 0 below. Save.
  - There is no need to similarly recalibrate the threshold for the MedSup predictions because the validation dataset had sufficient (27/100) examples of these data.

**Step 5: Run the keyword prioritization script “PFAS_Keyword_Prioriziation.py”**

- This Python script depends on the following input files:
  - “PFAS 15K Master List _Revised 2023-05-16.xlsx”
    - This workbook contains master lists of chemical names and synonyms for the current and previous assessments
  - “PFAS_15K_Unclassified_Relevance_MedSup_Predictions.csv”
    - This is the combined AI output created and adjusted in the above steps.
- Modify the directory paths in lines 82 and 154 of the script to reflect the location of the above two files.
- Run the script
- Store the output files
  - PFAS_15K_Unclassified_Relevance_MedSup_Predictions_All_w_Keyword_Tags.csv
  - PFAS_15K_Unclassified_Relevance_MedSup_Predictions_PreviousOnlyRemoved_w_Keyword_Tags.csv
- Optional step:
  - Convert the above output csv files into Excel .xlsx files and filter to show only PECO relevant studies in descending predicted probability of relevance as required.

# Systematic Evidence Map Methods

The methods below are reproduced from our earlier 150+ PFAS SEMs[^1-3^](#_ENREF_1) and the Expanded PFAS SEM[^4^](#_ENREF_4) because this project is a continuation of that work. The methods have been reproduced below and only adjusted as appropriate for the needs of this evidence map (e.g., chemical names, literature search dates).

## Literature Screening

Studies that met population, exposure, comparator, and outcome (PECO) criteria or supplemental material content from TIAB screening were then imported into DistillerSR for more specific title/abstract (TIAB) tagging (i.e., to separate studies meeting PECO criteria vs. supplemental content and to tag the specific category of supplemental content and, if necessary, the chemical). Supplemental content tags are described in Table 2 of the main manuscript. For studies meeting PECO criteria at the DistillerSR TIAB level, full-text articles were retrieved through the U.S. Environmental Protection Agency (EPA) HERO database. References that were not able to be retrieved within 45 days were not considered further. There was no author outreach for individual studies. Records identified through the European Chemicals Agency (ECHA) retrieval process were imported directly into DistillerSR for full-text screening.

Both TIAB and full-text screening were conducted by two independent reviewers. At all levels (SWIFT-Active Screener TIAB, DistillerSR TIAB, and DistillerSR full-text review), any conflicts in screening were resolved by discussion between the two independent reviewers; a third reviewer was consulted if any conflicts remained thereafter. Conflicts between screeners in applying the potentially relevant supplemental tags were resolved by discussion at both the TIAB and full-text levels, erring on the side of over tagging at the TIAB level. At the TIAB level, articles without an abstract were screened based on title (title should indicate clear relevance) and number of pages (articles two pages in length or less were assumed to be records with no original data). For additional information, please see Table 2 for supplemental categorization information. All studies identified as supplemental material at the TIAB and full-text levels were tagged to their respective chemical(s) using the preferred chemical names. All studies identified as PECO relevant were tagged to the preferred chemical name after the full-text screening stage. Of note, supplemental references that did not list a specific PFAS in the TIAB (i.e., included terms like “PFAS”) were tagged to “chemical not specified.” For these supplemental references, if any PFAS were specified in the abstract, references were tagged only to those chemicals, even though it was possible that additional PFAS chemicals were reported in the full text. All chemical tagging was reviewed by an expert in chemistry (with a Ph.D. or similar credential). When chemical identity was unclear, the study authors were contacted to resolve the chemical species. A full report of the literature tagging is available in HAWC[^5^](#_ENREF_5) and in Excel Table AS8. Note that many studies have complex designs, so a single study can be tagged and extracted to represent all health outcome data reported (e.g., studies may include information on pregnant women and maternal–child data).

## Distiller Literature Inventory

Studies that met PECO criteria after full-text review were summarized using custom forms “Distiller literature inventory standard operating procedure (SOP) for PFAS Evidence Map (abbreviated)”] in DistillerSR. For mammalian bioassay studies, the following study summary information was captured in a literature inventory: PFAS assessed, study type [acute (<24 hours), short term (1–30 days), subchronic (30–90 days), chronic (>90 days), developmental, peripubertal, multigenerational], route of exposure, species, sex, and health system(s) assessed (described in Table S2). For epidemiological studies, the following study summary information was captured in a literature inventory: PFAS assessed, sex, population, study design (Table S3), exposure measurement (e.g., blood, feces), and health system(s) assessed. For epidemiology studies, literature inventory was a high-level summary that did not confirm results relevant for full data extraction. As a result, more health outcomes may have been summarized at this level compared with the subsequent data extraction step. Summaries were then extracted into DistillerSR by one team member, and the extracted data were quality checked by at least one other team member. The data from these summary literature inventories were exported from DistillerSR to an Excel format and were then modified and transformed using Excel’s “Get and Transform” features for import into Tableau visualization software (version 2024.2.1; Tableau Software LLC). These data transformations include pivoting multiple columns of data to single columns, appending data from multiple literature inventories, and merging detailed reference information and chemical ID information into the dataset.

## Study Evaluation and Data Extraction

### Mammalian Bioassay Studies

Study evaluation was conducted by two reviewers using the EPA’s version of Health Assessment Workspace Collaborative (HAWC).[^6^](#_ENREF_6) Reviews were made by toxicologists with multiple years of experience in developing chemical human health assessments. For each study evaluation domain, at least two reviewers reached a consensus rating of “Good,” “Adequate,” “Deficient,” “Not Reported,” or “Critically Deficient,” as defined in HAWC. Key study evaluation considerations included potential sources of bias (factors affecting the magnitude or direction of an effect in a systematic way) and insensitivity (factors limiting detection of a true effect). The evaluated domains for toxicology studies included: Reporting Quality, Allocation, Blinding, Confounding/Variable Control, Selective Reporting/Attrition, Chemical Administration & Characterization, Study Design Applicability (exposure timing, frequency, and duration), Outcome Assessment, and Results Presentation. Core and prompting questions used to guide the judgment for each domain (e.g., reporting quality, confounding, outcome assessment) are described in more detail in the Integrated Risk Information System (IRIS) Handbook,[^7^](#_ENREF_7) and in the standard template language that is used for systematic evidence maps developed by EPA ORD[^8^](#_ENREF_8) and have only been adjusted, where appropriate, for the specific needs of this systematic evidence map (SEM). After a consensus rating was reached, the reviewers considered the identified strengths and limitations to reach an overall study confidence rating of “High,” “Medium,” “Low,” or “Uninformative” for each health outcome. The ratings, which reflect a consensus judgment between reviewers, are defined in the IRIS Handbook.[^6^](#_ENREF_6) The definitions below follow standard template language that is used in systematic evidence maps developed by the EPA[^8^](#_ENREF_8)^;^ [^9^](#_ENREF_9) and have only been adjusted, where appropriate, for the specific needs of this SEM.

- High: A well-conducted study with no notable deficiencies or concerns identified for the outcome(s) of interest; the potential for bias is unlikely or minimal, and the study used sensitive methodology. “High” confidence studies generally reflect judgments of “Good” across all or most evaluation domains.
- Medium: A study where some deficiencies or concerns were noted for the outcome(s) of interest, but the limitations are unlikely to be of a notable degree. Generally, “medium” confidence studies will include “Adequate” or “Good” judgments across most domains, with the impact of any identified limitation not being judged as severe.
- Low: A study where one or more deficiencies or concerns were noted for the outcome(s) of interest, and the potential for bias or inadequate sensitivity could have a significant impact on the study results or their interpretation. Typically, “Low” confidence studies would have a “Deficient” evaluation for one or more domains, although some “medium” confidence studies may have a “Deficient” rating in domain(s) considered to have less influence on the magnitude or direction of the results. Generally, in an assessment context (or a full systematic review), “Low” confidence results are given less weight in comparison with high or medium confidence results during evidence synthesis and integration and are generally not used as the primary sources of information for hazard identification or derivation of toxicity values unless they are the only studies available. Studies rated as “Low” confidence only because of sensitivity concerns about biases toward the null would require additional consideration during evidence synthesis.
- Uninformative: A study where serious flaw(s) make the results unusable for informing hazard identification for the outcome(s) of interest. Studies judged “Critically deficient” in any evaluation domain will almost always be classified as “Uninformative” (see explanation above). Studies with multiple “Deficient” judgments across domains may also be considered “Uninformative.” As mentioned above, although outside the scope of this SEM, in an assessment or full systematic review, uninformative studies would not be considered during the synthesis and integration of evidence for hazard identification or for dose response but might be used to highlight possible research gaps. Thus, data from studies deemed “Uninformative” are not depicted in the results displays included in this SEM.

Rationales for each study evaluation classification, including a description of how domain ratings impacted the overall study confidence rating are available in Excel Table AS9 and are documented and retrievable in HAWC.[^5^](#_ENREF_5)

Data extraction was conducted for mammalian bioassay studies by two members of the evaluation team using the EPA’s version of HAWC,[^5^](#_ENREF_5) a free and open source web-based software application that facilitates the management of literature assessments for environmental pollutants. Data extracted included basic study information (e.g., full citation, funding, author-reported conflicts of interest); experiment details (e.g., study type, chemical name, chemical source, and purity); animal group specifics (species, strain, sex, age at exposure and assessment, husbandry); dosing regimen; end points evaluated; and results (qualitative or quantitative) by end point. Authors were contacted if clarifications were needed and a record of this outreach is indicated for the respective studies in HAWC.[^5^](#_ENREF_5)

### Epidemiology Studies

The same approach was used for evaluation of all epidemiology studies. These methods have been summarized from [Radke et al.^3^](#_ENREF_3) For epidemiological studies, the top two rating levels (“Good” or “Adequate” and “High” or “Medium” confidence) were combined for the final evaluation, so there were ultimately three rating levels instead of four. This action was taken because the majority of studies had been evaluated for previous systematic review or evidence mapping projects and the evaluations were compiled from multiple projects. The evaluations were updated to reflect appropriate ratings for the PFAS included in this SEM, but because evaluation decisions may have differed slightly across projects due to between-reviewer variability, project goals, and evolving evaluation criteria, the merging of the top two levels is intended to improve consistency across studies within this project. The evaluated domains for epidemiology studies were participant selection, exposure measurement, outcome ascertainment, confounding, analysis, selective reporting, and sensitivity; detailed considerations for each domain are available in the Systematic Review Protocol for PFAS IRIS Assessments.[^10^](#_ENREF_10)

The epidemiological studies that were determined to meet PECO criteria after full-text review underwent data extraction for all included PFAS using a structured form in DistillerSR as described in [Radke et al.^3^](#_ENREF_3) The form captured citation information, study design characteristics (population, study design, country, year of data, exposure measurement type, exposure levels, outcome measures, sample size), whether the paper presented correlations across PFAS (though the actual correlations were not extracted), covariates included in statistical modeling, and quantitative results (effect estimate, confidence interval). Data extraction was performed by a trained member of the team (primary extractor) and checked by a second member for completeness and accuracy (QC extractor). Authors were not contacted for information that was not reported in a study unless clarifications were needed for apparent typos. The data from these extractions were exported from DistillerSR to an Excel format and then transformed for import into Tableau visualization software. The data transformations included pivoting multiple columns of data to single columns and merging detailed reference information and chemical ID information into the data set. The available evidence was visualized in Tableau, and the full extraction file is available in Excel Table AS11.

## ECHA Retrieval Instructions

A search of the ECHA registered substances database was conducted using the CASRN of each chemical. Further detailed documentation is detailed below. The registration dossier associated with the CASRN was retrieved by clicking the chemical name, navigating to a chemical’s key dataset and selecting the Registration, Evaluation, Authorisation, and Restriction of Chemicals (REACH) registered substance factsheets. The General Information tab and all sub-pages under the Toxicological Information tab were downloaded in PDF format, and data were extracted from each dossier page and used to populate an Excel tracking sheet. Extracted fields included data from the General Information page regarding the registration type and publication dates, and on a typical ECHA dossier page the primary fields reported in the administrative data, data source, and effect levels sections.

Rows of extracted data received an initial review for potential relevance. ECHA dossiers without information under the ToxCategory column were excluded from review as these refer to data extracted from the General Information tab. Toxicological and Endpoint Summary pages, study protocols, and dossiers with data waiving were also excluded from review. When a reference that was considered relevant reported data from a named study or lab report, a citation for the full study was either retrieved or generated in HERO and verified that it was not already identified from the peer-reviewed literature search prior to moving forward to screening in DistillerSR. If citation information was not available and a full-text document could not be retrieved, a citation was created in HERO using the information provided in the ECHA dossier. The generated PDF for the dossier was used as the full text for screening.

**Retrieve References:**

1. Enter CASRN of your assigned chemical, Search for chemicals. <https://echa.europa.eu/information-on-chemicals>
2. Click on the Chemical name.
   1. In the tracking file, copy the chemical name into Column E: Preferred_name.
   2. If there are multiple rows for a chemical, select the option with an ‘Active’ registration status and a ‘Full’ registration type. If these are not available, select the most recently updated option.
3. Once you have clicked on the correct chemical option, scroll down to the Key Datasets.
   1. If the Key dataset “REACH registered substance factsheets” is greyed out, you will record No Results in the Status column of tab 1 and move on to another chemical. Otherwise proceed to Step 4.
4. Click the REACH registered substance factsheets tab.
   1. Fill out the **Chemical Information** related to the search in the Excel tracking sheet tab 2 in columns A–F.
      1. This data will be repeated for each of the data rows for a chemical so you can copy/paste into each of your rows for one chemical.
5. Identify **General Information** (left navigation menu selection, shown below); you may have to further click into administrative Information, and add to Excel tracking sheet columns H–L.
   1. This data will be repeated for each of the data rows for a chemical, you can copy/paste into each of your rows for one chemical.
   2. Use the **General Information** site URL (the top search bar) in column F: URL.Searched.
   3. Use the following date format for all entries requiring it: MM-DD-YYYY.
6. Click the **Toxicological Information** (left navigation menu);. If there are references, there will be bolded options in the expanded field.
   1. If there are no bold headings, enter NONE in column M, and move to the next chemical.
   2. If there are bold headings, expand each bold arrow. Each bullet entry will receive a row or rows of collected data in the Excel tracking file.
      1. You do not need to create a PDF or fill a row for **Endpoint Summary** bullets.
      2. In the example below, Acute Toxicity is expanded. Click on each bullet (e.g., Acute Toxicity: Oral; Acute Toxicity: inhalation) and record the data.
      3. If your chemical has a list of results under a bullet, fill out a row and PDF for **each** of the results.
         - In this example, there are multiple results under the **Acute Toxicity: Oral** bullet as you expand the arrows to see how many lines you will need to enter for each type—for this example there are four rows for Acute Toxicity: oral. Add the “001 Weight of evidence| Experimental result” to column N: *Endpoint Category* (i.e., Acute Toxicity: oral|001 Weight of evidence| Experimental result), then continue with a new row for Acute Toxicity: oral | 002 Weight of evidence | Experimental result, and so on.
      4. Occasionally you might see a dropdown choice of “S-01 Summary” and you will add a row for this as well *unless* it does not have any administrative data or reference information, in which case there’s nothing to record and it does not need a pdf.
      5. Occasionally you might see a dropdown choice of “004 Disregard | Experimental result” and you will add a row for this as well.
      6. Occasionally you might see a dropdown choice of “005 Key|Read Across…” and information such as the "reliability" and "study period" are not posted in the top administrative section, but below the "Cross-reference" section. Complete as many of the columns as you can using the information in the “Cross-reference” section.
      7. Move to the next bold heading, in this case, Irritation/corrosion and repeat the process.
      8. For each entry, fill out **Administrative Dat**a (columns M–W), and **Data Source** (columns X–AC).
   3. If you have data waiving information vs. type, adequacy of study, etc., you just need to fill out Data Waiving and Justification for Data Waiving, not Justification for Type of Information.
   4. If your chemical does not have data for any of the columns, leave the cell blank.
   5. If you identify typos such as a year that is “1087” or a misspelled word, you can enter the correct date/spelling in these columns.
   6. Make sure you scroll down to the bottom of the page for each entry; in one instance there was a reference that was off kilter but needed to be included as well (see example below)
7. Create a PDF of each entry. Click the printer icon (upper right) and save as a PDF.
   1. PDF naming convention: ECHA_CASRN_#1. Each row is a new result. Increase the final # (i.e., #2, #3, etc.).
   2. When a record has more than one **data source,** create a row for each Data source (to accommodate different reference information). Only one PDF is required (the PDF process expands and includes all reference data, even if you have only expanded one).
8. When all data has been added to the tracking file for a chemical, indicate **Complete** in Column E: Status on the Chemical & Assignments tab in the tracking file.

Later steps, after all data has been collected:

1. Filtering (after all data has been collected and final # of results is known)
2. Create HERO ID Records

## DeDuper

The Deduper methods below are reproduced from our earlier PFAS SEMs[^1-3^](#_ENREF_1) and the Expanded PFAS SEM[^4^](#_ENREF_4) because this project is a continuation of that work.

DeDuper is a tool developed by ICF, freely available to clients by request, which incorporates machine learning to identify and reconcile duplicate references in multiple database search results. The DeDuper tool uses a two-phase approach to identify duplicates: 1) locates duplicates using automated logic, and 2) employs machine learning to predict likely duplicates which are then verified manually.

ICF automated the deduplication process by applying a novel pipeline of deduplication algorithms. Deduplication is performed in two phases and can be evaluated for precision (proportion of true positives within all records predicted to be duplicates) and recall (i.e., sensitivity, the proportion of true positives within all records that were actual duplicates). Phase 1 prioritizes precision, by defining a set of exact match rules that operate on a limited set of fields in standard bibliographic citation files (lower-case title, publication year, first author, and starting page). The second phase of the pipeline uses machine learning to flag predicted matches as duplicates for removal and entity resolution on research information systems (RIS) files; this phase is designed to maximize recall by applying algorithms to more comprehensively detect the remaining duplicates. The algorithms (i) intelligently limit the number of record comparisons to ensure speedy performances, and (ii) apply machine learning and fuzzy comparisons to learn and apply user tolerance for differences in select fields that are not exact matches (e.g., title, author names, and publication year).

This tool utilizes the Python Dedupe package to compare references field by field to identify pairs that have the highest likelihood of being a duplicate record. During training, the program:

- Groups records based on editing distance across specific fields (i.e., affine gap distance).
- Proposes grouped records as duplicates to the user for verification and changes the relative weights of various features of each entry based on the user’s response.
- Identifies the probability of duplication based on the distance between each record cluster after sufficient testing and allows for a more conservative or a more aggressive deduplication process based on threshold probabilities.

For rapid processing, ICF created a large training dataset from deduplication efforts on previous projects that save the user the need for training the model for each new run.

The algorithms underlying DeDuper—and its application in a case study involving a literature search related to diisononyl phthalate (DINP)—were presented at the 2018 Society of Toxicology (SOT) meeting.[^11^](#_ENREF_11) In this case study, ICF applied DeDuper to a set of 30,000 references in which duplicates had been previously identified manually. Phase 1 achieved a precision of 100%, although recall was limited at 76%. Phase 2 achieved a recall of 99% with a 48% precision. After accounting for manual review to remove false positives from the machine-identified duplicates pile, the combined pipeline realized an 82% efficiency gain. ICF normalized these results based on maximum possible efficiency gains (which depends on the proportion of duplicate groups in the original dataset) to estimate a specificity of 85%.

## Distiller Literature Inventory SOP for PFAS Universe Evidence Map (Abbreviated)

**Step 1: View list of studies.** To view the list of studies, go to Review → Level 3 → Health Literature Inventory Extraction form. Alternatively, you can access the list from the assessment home page. Under Level 3, click on “Unreviewed” to access the list of studies that have not been extracted yet.


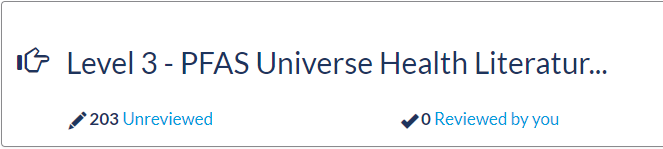


**Step 2: Select a study for extraction.** Select a study by clicking on it, and a new tab will open.


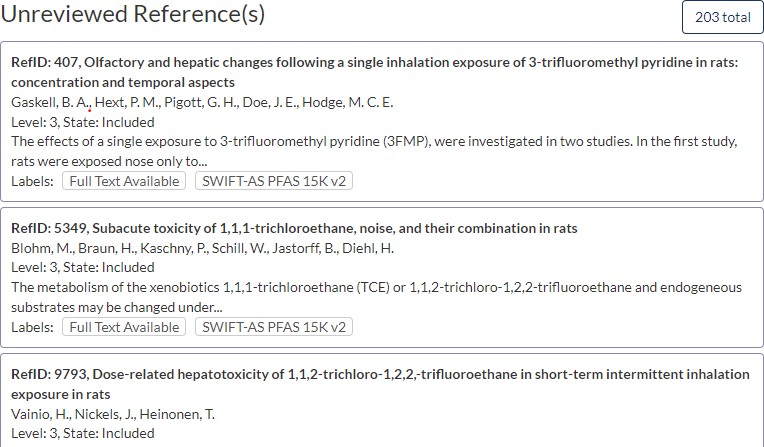


Once the new tab opens, click on the “HERO link” to download the PDF. Or, you can search the RefID at [https://heronet.epa.gov/heronet/index.cfm/search.](https://heronet.epa.gov/heronet/index.cfm/search)


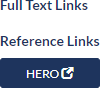


**Step 3: Data extraction – Part 1**

**Step 3a: Enter Author information.** Use the format specified in the Distiller form.


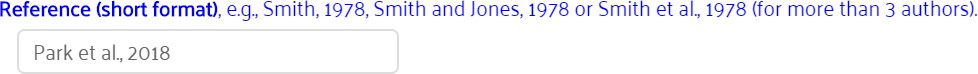


**Step 3b: Select the evidence stream(s) that apply** – Epi (human study); Tox (animal study).

**Step 3c: Select all supplemental tags that apply** – See **Table 2** for the major categories of potentially relevant supplemental material.

**Step 3d: Select other PFAS chemicals that were evaluated but not included in our screening.**

**Step 3e: Select “No” for the QC question if you are doing the primary extraction.**

Select “No” to indicate that you are doing the initial data extraction. *If you find during your review that the study does not meet PECO criteria, it should not be extracted. Select “study is not PECO relevant: update full-text screening tags” to indicate that the study needs to be retagged at the full-text review level. Refer to* **Table 1** *for PECO criteria.*

**Step 4: Data extraction – Part 2**

**Step 4a: Add a subform.**

To begin extracting data, click on “Add” and a subform will appear. You are now ready to enter information into the form.


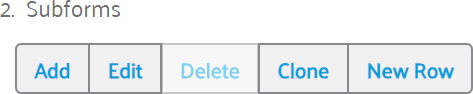


**Step 4b: Enter evidence type.** This is a dropdown menu – human or animal.


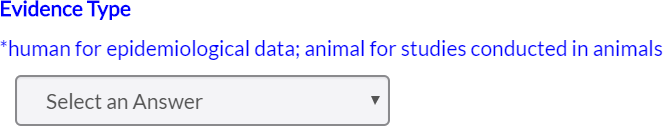


Note: For this project, select *“human (abbreviated extraction)”* for human studies. Ignore

“Human (full extraction).”

**Step 4c: Specify the relevant PFAS Universe chemical. Create a new subform for each relevant chemical.**

For each relevant chemical:

1. Copy and paste the full chemical name used in the paper.

- Do not include the abbreviation unless that is all the paper uses.

1. Copy and paste the “preferred name” from the PFAS Universe Master List.
2. Note the chemical abbreviation used in the paper, if available (leave blank if no abbreviation is used in the paper).
3. Note the CASRN provided in the paper, if available (leave blank if not provided).

**The next questions about study design will differ depending on whether the study is in humans or animals.**

**Human studies (abbreviated form):**

**Step 4d (Human): Enter sex, population, and study design.** Select sex (ignore “female dam”), population, and study design from dropdown menus.


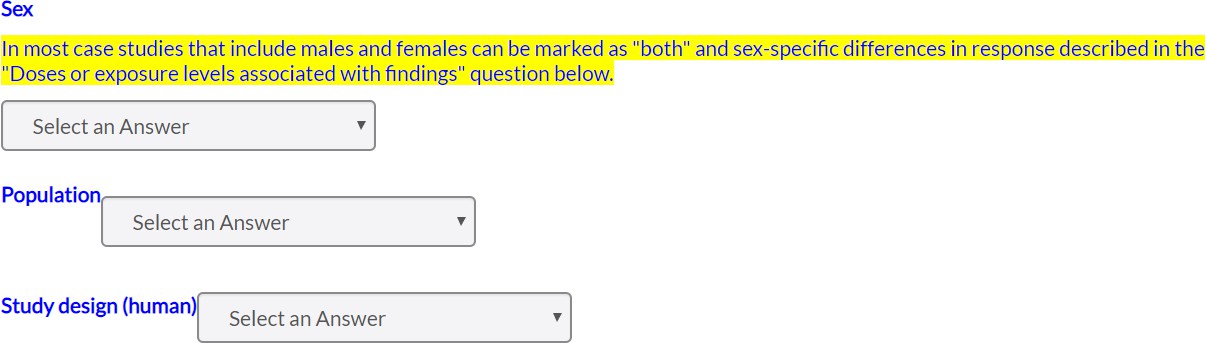


**NOTE: Please refer to Table S3 for guidance in selecting the study design.**

**Step 4e (Human): Exposure measurement.** Enter information on the exposure measurement as prompted in the Distiller form. If biomonitoring, select the biomonitoring matrix.


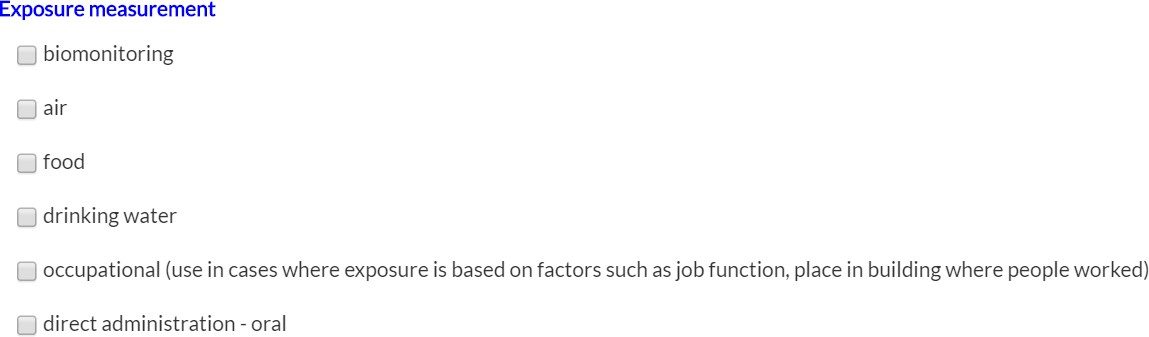


**Step 4f (Human): Select health outcome.** See **Table S2** for what kind of endpoints are grouped under which health outcomes. Please create a separate form for each outcome. See Step 6 on how to clone a form.


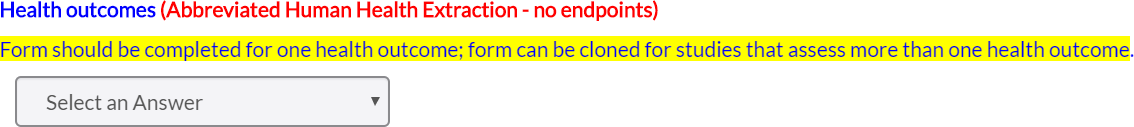


**Animal studies (abbreviated):**

**Step 4d (Animal): Enter study design.** This is a dropdown menu.

Note: If a study presents multiple experiments with different exposure durations, e.g., subchronic and chronic, these experiments need to be entered in separate forms. Please indicate which experiment you are referring to in the comment box. Also, see instructions below to clone the form.


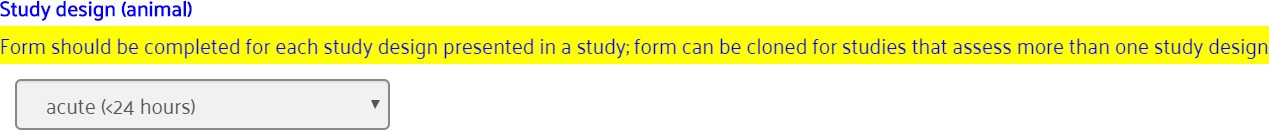


“Developmental” includes F0 and F1 data from one generation repro/developmental study designs.

“Multigenerational” includes repro/developmental studies that span two generations and beyond.

Both developmental and multigenerational study designs will have a free text box in which you will indicate the generation (F0, F1, etc).

**Step 4e: Enter the route, species, sex, and health outcomes.**


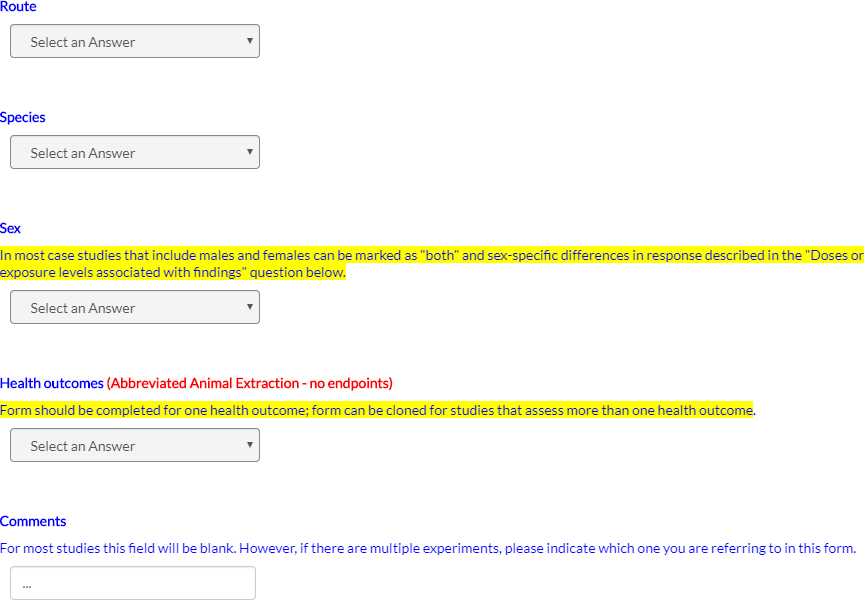


Note: See **Table S2** for what kind of endpoints are grouped under which health outcomes.

**Step 5: Click “Submit”** when you have completed the subform (which will save it).


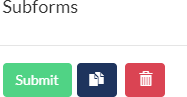


Note: If you are done with all your subforms, you must also click “Submit” at the top of the page (see Step 8).

**Step 6: Cloning the form.** If you have more than one chemical, study design, or health outcome, you can “clone” the form to avoid having to reenter some information. To do this, select the form you want to clone and click “Clone” near the top of the page. Alternatively, you can start a new blank form by clicking “Add.”


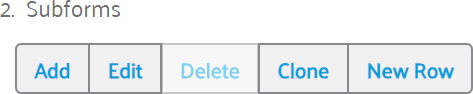


**Step 7: Editing the form:** If you need to change information or edit a form you have completed, you can click on the arrow within a form to edit.


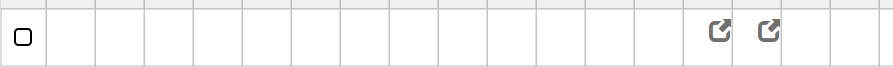


**Step 8: When everything is complete, click “Submit” at the top of the page.** If you click “Save All,” the study will be saved/unsubmitted in your “Unreviewed” pile. You can complete it another time, but you must submit it when you are done.


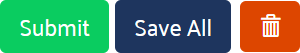


# Supplemental Tables

Table S1. PFAS under assessment by U.S. EPA that are not included in this systematic evidence map (reprinted from Carlson et al. and Shirke et al.)[^1^](#_ENREF_1)^;^ [^4^](#_ENREF_4)

| **PFAS name** | **CASRN** | **DTXSID** | **U.S. EPA assessment activity** |
| --- | --- | --- | --- |
| Perfluorobutanoic acid (PFBA) | 375-22-4 | DTXSID4059916 | IRIS Assessment*^a^*  U.S. EPA (2022)[^12^](#_ENREF_12) |
| Ammonium  perfluorobutanoate | 10495-86-0 | DTXSID10893420 |  |
| Perfluorohexanoic acid (PFHxA) | 307-24-4 | DTXSID3031862 | IRIS Assessment*^a^*  U.S. EPA (2022)[^13^](#_ENREF_13) |
| Ammonium perfluorohexanoate | 21615-47-4 | DTXSID90880232 |  |
| Sodium perfluorohexanoate | 2923-26-4 | DTXSID3052856 |  |
| Perfluorooctanoic acid (PFOA) | 335-67-1 | DTXSID8031865 | SDWA National Primary Drinking Water Regulation  U.S. EPA (2024)[^14^](#_ENREF_14) |
| Ammonium perfluorooctanoate | 3825-26-1 | DTXSID8037708 |  |
| Perfluorononanoic acid (PFNA) | 375-95-1 | DTXSID8031863 | IRIS Assessment*^a^*  U.S. EPA (2024)[^15^](#_ENREF_15) |
| Ammonium perfluorononanoate | 4149-60-4 | DTXSID20880205 |  |
| Sodium  heptadecafluorononanoate | 21049-39-8 | DTXSID50896632 |  |
| Perfluorodecanoic acid (PFDA) | 335-76-2 | DTXSID3031860 | IRIS Assessment*^a^*  U.S. EPA (2024)[^16^](#_ENREF_16) |
| Ammonium perfluorodecanoate | 3108-42-7 | DTXSID60880027 |  |
| Sodium perfluorodecanoate | 3830-45-3 | DTXSID20880028 |  |
| Perfluorobutanesulfonic acid (PFBS) | 375-73-5 | DTXSID5030030 | ORD Assessment  U.S. EPA (2021)[^17^](#_ENREF_17) |
| Perfluorobutanesulfonate | 45187-15-3 | DTXSID60873015 |  |
| Ammonium  Perfluorobutanesulfonate | 68259-10-9 | DTXSID3071355 |  |
| Potassium  Perfluorobutanesulfonate | 29420-49-3 | DTXSID3037707 |  |
| Perfluorohexanesulfonic acid (PFHxS) | 355-46-4 | DTXSID7040150 | IRIS Assessment*^a^*  U.S. EPA (2025)[^18^](#_ENREF_18) |
| Potassium  Perfluorohexanesulfonate | 3871-99-6 | DTXSID3037709 |  |
| Perfluorooctanesulfonic acid (PFOS) | 1763-23-1 | DTXSID3031864 | SDWA National Primary Drinking Water Regulation  U.S. EPA (2024)[^19^](#_ENREF_19) |
| Perfluorooctanesulfonate | 45298-90-6 | DTXSID80108992 |  |
| Ammonium  Perfluorooctanesulfonate | 29081-56-9 | DTXSID9067435 |  |
| Lithium  Perfluorooctanesulfonate | 29457-72-5 | DTXSID2032421 |  |
| Potassium  Perfluorooctanesulfonate | 2795-39-3 | DTXSID8037706 |  |
| Sodium  Perfluorooctanesulfonate | 4021-47-0 | DTXSID50635462 |  |
| Perfluoro-2-methyl-3-oxahexanoic acid (“GenX Chemicals”) | 13252-13-6 | DTXSID70880215 | OW Assessment  U.S. EPA (2021)[^20^](#_ENREF_20) |
| Ammonium perfluoro-2-methyl-3-oxahexanoate | 62037-80-3 | DTXSID40108559 |  |
| Lithium bis [(trifluoromethyl)sulfonyl] azanide (HQ-115) | 90076-65-6 | DTXSID8044468 | ORD Assessment  U.S. EPA (2023)[^21^](#_ENREF_21) |
| 1,1,1-Trifluoro-N-[(trifluoromethyl)sulfonyl] methanesulfonamide (TFSI) | 82113-65-3 | DTXSID2045026 |  |

Note: These PFAS were deprioritized from the evidence map due to scoping considerations as described in the Methods section. These PFAS chemicals are undergoing more in-depth analyses as part of specific U.S.EPA chemical assessments. Interested readers can access assessments or draft materials at the identified citations. IRIS, Integrated Risk Information System; SDWA, Safe Drinking Water Act.

*^a^The URLs provided in the citation records provide links to the chemical assessment page for PFAS under assessment by the U.S. EPA Office of Research and Development’s (ORD) Integrated Risk Information System (IRIS) Program. Information on timelines for development of the assessments can be found in the IRIS Program Outlook,*[^22^](#_ENREF_22) *which is updated three times a year.*

Table S2. Categorization for grouping endpoints under health outcomes

| **Health effect category** | **Examples of relevant endpoints** | **Notes** |
| --- | --- | --- |
| **Systemic Effects** | - Body weight (not reproductive/developmental) - Mortality, survival, or LD50s - Growth curve - Clinical observations (e.g., lethargy; oral discharge) | - Clinical chemistry endpoints are under Hepatic or Hematologic - Maternal body weights are under Reproductive - Pup body weight endpoints are under Developmental - Pathology (including gross lesions) is organ-specific |
| **Carcinogenicity** | - Tumors - Precancerous lesions (e.g., dysplasia) | – |
| **Cardiovascular Effects** | - Heart weight - Heart, artery, and vein histopathology - Blood pressure - Serum cholesterols and lipids | - Other blood measures are under Hepatic, Immune, or Hematologic |
| **Dermal Effects** | - Skin sensitivity - Skin histopathology | – |
| **Developmental Effects** | - Pup viability/survival, or other birth parameters - Pup weight or growth (into adulthood if developmental exposure) - Developmental landmarks (e.g., eye opening) not including markers for other organ/system-specific toxicities - Skeletal, visceral, or gross abnormalities in fetuses/pups | - Histopathology and markers of development specific to other systems are organ/system-specific (e.g., vaginal opening is under Female Reproductive; tests of sensory maturation are under Nervous System) |
| **Endocrine Effects** | - Thyroid/adrenal weight - Thyroid/adrenal histopathology - Hormonal measures in any tissue or blood (nonreproductive) - Stress-related factors in blood (e.g., glucocorticoids or other adrenal markers) | - Reproductive hormones are under Reproductive |
| **Gastrointestinal Effects** | - Stomach and intestine weight - Stomach and intestine histopathology | – |
| **Hematologic Effects** | - Corpuscular volume - Red blood cells - Serum hematocrit or hemoglobin - Serum platelets or reticulocytes - Serum biochemical measures (sodium, calcium, phosphorus) - Blood coagulation markers | - White blood cell count and globulin are under Immune - Serum lipids are under Cardiovascular - serum liver markers are under Hepatic |
| **Hepatic Effects** | - Liver weight - Liver histopathology - Liver tissue enzyme activity (e.g., catalase) or protein/dna content - Liver enzymes (ALT; AST) - Liver biochemical markers (albumin; glycogen) - Liver tissue lipids (triglycerides, cholesterol) | - Serum lipids are under Cardiovascular - Biochemical markers such as albumin or glucose are under Hematological - Liver tissue cytokines are under immune - Serum glucose is under Metabolic |
| **Immune Effects** | - Host resistance - Allergic, autoimmune, or infectious disease - Hypersensitivity - Lymphocyte phenotyping or proliferation - Lymphoid tissue weight, histopathology, cell counts - Immune functional assays (e.g., antibody production, natural killer cell function, delayed-type hypersensitivity [DTH], mixed leukocyte reaction [MLR], cytotoxic T lymphocyte [CTL], phagocytosis or bacterial killing by monocytes) - Immune responses in the respiratory system (includes asthma) - White blood cell counts - Serum immunological factors or cytokines - Immune cell counts or immune-specific cytokines in non-lymphoid tissues - Bone marrow histopathology and cell counts | - Red blood cells are under Hematological - Non-immune measures of pulmonary function are under Respiratory |
| **Metabolic Effects** | - Pancreatic effects relevant to diabetes - Induced-obesity or body mass index - Free fatty acids - Serum glucose or insulin, or other measures related to diabetes | – |
| **Musculoskeletal/Connective Tissue** | - Bone weight and histopathology - Muscular histopathology | – |
| **Neurological Effects** | - Brain weight - Brain histopathology - Nervous system histopathology - Behavioral measures (including functional observation battery and cage-side observations) | – |
| **Ocular Effects** | - Eye histopathology - Vison changes - Eye irritation | – |
| **Renal Effects** | - Kidney weight - Kidney histopathology - Urinary tract histopathology - Bladder weight and histopathology - Urinary measures (e.g., protein; volume; ph; specific gravity) | – |
| **Reproductive Effects** | - Dam health, body weight, food consumption - Reproductive organ weight - Reproductive organ histopathology - Markers of sexual differentiation or maturation (e.g., preputial separation in males; vaginal opening or estrous cycling in females) - Mating parameters (e.g., success; mount latency) - Sperm parameters (e.g., counts; motility) - Reproductive hormones | - Birth parameters (e.g., litter size; resorptions; implantations; viability) are under Developmental - [NOTE: if data indicate altered birth parameters are likely attributable to female fertility, these data may be discussed under Female Reproductive] |
| **Respiratory Effects** | - Lung weight and histopathology - Nasal cavity histopathology | – |

Table S3. Types of study designs (human)

| **Study design** | **Description** |
| --- | --- |
| Cross-sectional | Exposure and outcome are examined at the same point in time in a defined study population. Cannot determine if exposure came before or after outcome. |
| Cohort | A group of people is examined over time to observe a health outcome. Everyone belongs to the same population (e.g., general U.S. population; an occupational group; cancer survivors). All cohort studies (prospective or retrospective) consider exposure data from before the occurrence of the health outcome. |
| Case-control | Cases (people with the health outcome) and controls (people without the health outcome) are selected at the start of a study. Exposure is determined and compared between the two groups. A case-control study can be nested within a cohort. |
| Ecological | The unit of observation is at the group level (e.g., zip code; census tract), rather than the individual level. Ecological studies are often used to measure prevalence and incidence of disease. Cannot make inferences about an individual’s risk based on an ecological study. |
| Controlled Trial | Exposure is assigned to subject and then outcome is measured. |

Table S4. Inventory of HAWC visuals by chemical in this SEM

| **PFAS**  **DTXSID** | **(Exposure route)**  **health category** | **HAWC URL** |
| --- | --- | --- |
| **(1E)-1-Chloro-3,3,3-trifluoro-1-propene**  DTXSID20888807 | (Inhalation)  Developmental, Reproductive, Whole Body | <https://hawc.epa.gov/summary/data-pivot/assessment/100500347/Figure-01a-1E-1-Chloro-333-trifluoro-1-propene/> |
|  | (Inhalation)  Cardiovascular, Dermal, Endocrine, Gastrointestinal, Hematologic, Hepatic, Immune, Metabolic, Musculoskeletal, Nervous, Ocular, Reproductive, Respiratory, Urinary, Whole Body | <https://hawc.epa.gov/summary/data-pivot/assessment/100500347/01b-Figure-01b-1E-1-Chloro-333-trifluoro-1-pr-6107/> |
|  | N/A | <https://hawc.epa.gov/summary/visual/assessment/100500347/1E-1-Chloro-333-trifluoro-1-propene-DTXSID20888807/> |
| **(1Z)-1-Chloro-2,3,3,3-tetrafluoroprop-1-ene**  DTXSID901032227 | (Inhalation)  Developmental, Whole Body | <https://hawc.epa.gov/summary/data-pivot/assessment/100500347/Figure-02-1Z-1-Chloro-2333-tetrafluoroprop-1--510c/> |
|  | N/A | <https://hawc.epa.gov/summary/visual/assessment/100500347/1Z-1-Chloro-2333-tetrafluoroprop-1-ene-DTXSID-ebe7/> |
| **(Z)-1,1,1,4,4,4-Hexafluoro-2-butene**  DTXSID00883476 | (Inhalation)  Developmental, Reproductive, Whole Body, Cardiovascular, Dermal, Hematologic, Hepatic, Metabolic, Musculoskeletal, Nervous, Ocular, Reproductive, Urinary | <https://hawc.epa.gov/summary/data-pivot/assessment/100500347/03-Figure-03-Z-111444-Hexafluoro-2-butene_Inh-ba31/> |
|  | N/A | <https://hawc.epa.gov/summary/visual/assessment/100500347/Z-111444-Hexafluoro-2-butene-DTXSID00883476/> |
| **1,1,1,2,3,3-Hexafluoropropane**  DTXSID00861931 | (Inhalation)  Developmental, Reproductive, Whole Body, Cardiovascular, Dermal, Endocrine, Gastrointestinal, Hematologic, Hepatic, Immune, Metabolic, Musculoskeletal, Nervous, Ocular, Respiratory, Special Senses, Urinary | <https://hawc.epa.gov/summary/data-pivot/assessment/100500347/04-Figure-04-111233-Hexafluoropropane_Inhalation/> |
|  | N/A | <https://hawc.epa.gov/summary/visual/assessment/100500347/111233-Hexafluoropropane-DTXSID00861931/> |
| **1,1,1,2-Tetrafluoroethane**  DTXSID1021324 | (Inhalation)  Developmental, Reproductive, Whole Body | <https://hawc.epa.gov/summary/data-pivot/assessment/100500347/05a-Figure-05a-1112-Tetrafluoroethane_Inhalation/> |
|  | (Inhalation)  Cardiovascular, Endocrine, Hematologic, Hepatic, Immune, Metabolic, Nervous, Ocular, Reproductive, Respiratory, Urinary, Whole Body, Dermal, Gastrointestinal, Musculoskeletal | <https://hawc.epa.gov/summary/data-pivot/assessment/100500347/05b-Figure-05b-1112-Tetrafluoroethane_Inhal/> |
|  | (Inhalation)  Hematologic, Hepatic, Metabolic, Multi-System, Ocular, Reproductive, Respiratory, Urinary, Whole Body, Cardiovascular, Endocrine, Gastrointestinal, Immune, Musculoskeletal, Nervous, Ocular | <https://hawc.epa.gov/summary/data-pivot/assessment/100500347/05c-Figure-05c-1112-Tetrafluoroethane_Inhalati/> |
|  | (Oral)  Dermal, Endocrine, Hepatic, Immune, Nervous, Reproductive, Respiratory, Urinary, Whole Body | <https://hawc.epa.gov/summary/data-pivot/assessment/100500347/50-Figure-50-1112-Tetrafluoroethane_Oral/> |
|  | N/A | https://hawc.epa.gov/summary/visual/assessment/100500347/1112-Tetrafluoroethane-DTXSID1021324/ |
| **1,1,1,3,3,3-Hexafluoropropane**  DTXSID8052435 | (Inhalation)  Developmental, Reproductive, Whole Body, Cardiovascular, Dermal, Endocrine, Gastrointestinal, Hematologic, Hepatic, Immune, Metabolic, Musculoskeletal, Nervous, Ocular, Respiratory, Special Senses, Urinary | https://hawc.epa.gov/summary/data-pivot/assessment/100500347/06-Figure-06-111333-Hexafluoropropane_Inhalation/ |
|  | N/A | https://hawc.epa.gov/summary/visual/assessment/100500347/111333-Hexafluoropropane-DTXSID8052435/ |
| **1,1,1,3,3-Pentafluoropropane**  DTXSID6052110 | (Inhalation)  Cardiovascular, Developmental, Endocrine, Hepatic, Immune, Metabolic, Nervous, Reproductive, Respiratory, Urinary, Whole Body | https://hawc.epa.gov/summary/data-pivot/assessment/100500347/07a-Figure-07a-11133-Pentafluoropropane_Inhal-77a1/ |
|  | (Inhalation)  Cardiovascular, Dermal, Developmental, Endocrine, Gastrointestinal, Hematologic, Hepatic, Immune, Metabolic, Musculoskeletal, Nervous, Ocular, Reproductive, Respiratory, Urinary, Whole Body | https://hawc.epa.gov/summary/data-pivot/assessment/100500347/07b-Figure-07b-11133-Pentafluoropropane_Inhal-829c/ |
|  | N/A | https://hawc.epa.gov/summary/visual/assessment/100500347/11133-Pentafluoropropane-DTXSID6052110/ |
| **1,1,1-Trifluoroethane**  DTXSID9042047 | (Inhalation)  Developmental, Reproductive, Whole Body, Cardiovascular, Dermal, Endocrine, Gastrointestinal, Hematologic, Hepatic, Immune, Metabolic, Musculoskeletal, Nervous, Ocular, Respiratory, Urinary | https://hawc.epa.gov/summary/data-pivot/assessment/100500347/08-Figure-08-111-Trifluoroethane_Inhalation/ |
|  | (Oral)  Dermal, Endocrine, Hepatic, Immune, Nervous, Reproductive, Respiratory, Urinary, Whole Body | https://hawc.epa.gov/summary/data-pivot/assessment/100500347/51-Figure-51-111-Trifluoroethane_Oral/ |
|  | N/A | https://hawc.epa.gov/summary/visual/assessment/100500347/111-Trifluoroethane-DTXSID9042047/ |
| **1,1,2,2-Tetrafluoroethane**  DTXSID60883371 | (Inhalation)  Cardiovascular, Endocrine, Hematologic, Hepatic, Immune, Metabolic, Nervous, Reproductive, Respiratory, Urinary, Whole Body | https://hawc.epa.gov/summary/data-pivot/assessment/100500347/09-Figure-09-1122-Tetrafluoroethane_Inhalation/ |
|  | N/A | https://hawc.epa.gov/summary/visual/assessment/100500347/1122-Tetrafluoroethane-DTXSID60883371/ |
| **1,1,2-Trichloro-1,2,2-trifluoroethane**  DTXSID6021377 | (Inhalation)  Cardiovascular, Dermal, Hematologic, Hepatic, Metabolic, Multi-System, Nervous, Ocular, Respiratory, Urinary, Whole Body, | https://hawc.epa.gov/summary/data-pivot/assessment/100500347/10a-Figure-10a-112-Trichloro-122-trifluoroeth-9deb/ |
|  | (Inhalation)  Cardiovascular, Endocrine, Hematologic, Hepatic, Immune, Nervous, Respiratory, Urinary, Whole Body, Cardiovascular, Dermal, Gastrointestinal, Metabolic, Multi-System, Musculoskeletal, Ocular, Reproductive | https://hawc.epa.gov/summary/data-pivot/assessment/100500347/10b-Figure-10b-112-Trichloro-122-_Inhalat-90b6/ |
|  | N/A | https://hawc.epa.gov/summary/visual/assessment/100500347/112-Trichloro-122-trifluoroethane-DTXSID6021377/ |
| **1,1-Bis(trifluoromethyl)ethene**  DTXSID9052056 | (Inhalation)  Cardiovascular, Hepatic, Immune, Metabolic, Nervous, Reproductive, Respiratory, Urinary, Whole Body | https://hawc.epa.gov/summary/data-pivot/assessment/100500347/11-Figure-11-11-Bistrifluoromethylethene/ |
|  | N/A | https://hawc.epa.gov/summary/visual/assessment/100500347/11-Bistrifluoromethylethene-DTXSID9052056/ |
| **1,1-Difluoroethane**  DTXSID0024050 | (Inhalation)  Developmental, Hepatic, Multi-System, reproductive, Urinary, Whole Body | https://hawc.epa.gov/summary/data-pivot/assessment/100500347/12-Figure-12-11-Difluoroethane_Inhalation/ |
|  | N/A | https://hawc.epa.gov/summary/visual/assessment/100500347/11-Difluoroethane-DTXSID0024050/ |
| **1,2-Dichloro-1,1,2,2-tetrafluoroethane**  DTXSID8026434 | (Inhalation)  Cardiovascular, Dermal, Hematologic, Hepatic, Metabolic, Multi-System, Nervous, Ocular, Respiratory, Urinary, Whole Body, | https://hawc.epa.gov/summary/data-pivot/assessment/100500347/13-Figure-13-12-Dichloro-1122-tetrafluoroetha-afe6/ |
|  | N/A | https://hawc.epa.gov/summary/visual/assessment/100500347/12-Dichloro-1122-tetrafluoroethane-DTXSID8026434/ |
| **1,3,3,3-Tetrafluoroprop-1-ene**  DTXSID60936952 | (Inhalation)  Developmental, Reproductive, Whole Body | https://hawc.epa.gov/summary/data-pivot/assessment/100500347/14-Figure-14-1333-Tetrafluoroprop-1-ene_Inhalation/ |
|  | (Inhalation)  Cardiovascular, Dermal, Endocrine, Hematologic, Hepatic, Immune, Metabolic, Reproductive, Respiratory, Urinary, Whole Body, Gastrointestinal, Musculoskeletal, Ocular, | https://hawc.epa.gov/summary/data-pivot/assessment/100500347/14b-Figure-14b-1333-Tetrafluoroprop-1-ene_Inh-7509/ |
|  | N/A | https://hawc.epa.gov/summary/visual/assessment/100500347/1333-Tetrafluoroprop-1-ene-DTXSID60936952/ |
| **1,3-Dichloro-1,1,2,2,3-pentafluoropropane**  DTXSID6042028 | (Inhalation)  Cardiovascular, Dermal, Endocrine, Gastrointestinal, Hepatic, Immune, Metabolic, Musculoskeletal, Nervous, Ocular, Reproductive, Respiratory, Urinary, Whole Body, Auditory | https://hawc.epa.gov/summary/data-pivot/assessment/100500347/15-Figure-15-13-Dichloro-11223-pentafluoropro-bbff/ |
|  | N/A | https://hawc.epa.gov/summary/visual/assessment/100500347/13-Dichloro-11223-pentafluoropropane-DTXSID6042028/ |
| **1-Chloro-1,1-difluoroethane**  DTXSID9023960 | (Inhalation)  Cardiovascular, Dermal, Endocrine, Hematologic, Hepatic, Immune, Metabolic, Multi-System, Nervous, Ocular, Reproductive, Respiratory, Urinary, Whole Body | https://hawc.epa.gov/summary/data-pivot/assessment/100500347/16-Figure-16-22-Dichloro-111-trifluoroethane_-af26/ |
|  | N/A | https://hawc.epa.gov/summary/visual/assessment/100500347/1-Chloro-11-difluoroethane-DTXSID9023960/ |
| **2,2-Dichloro-1,1,1-trifluoroethane**  DTXSID7020712 | (Inhalation)  Developmental, Hepatic, Metabolic, Whole Body, Reproductive, Endocrine | https://hawc.epa.gov/summary/data-pivot/assessment/100500347/17a-Figure-17a-22-Dichloro-111-trifluoroethan-813f/ |
|  | (Inhalation)  Cardiovascular, Dermal, Endocrine, Gastrointestinal, Hepatic, Immune, Metabolic, Musculoskeletal, Nervous, Ocular, Reproductive, Respiratory, Urinary, Whole Body | https://hawc.epa.gov/summary/data-pivot/assessment/100500347/17b-Figure-17b-22-Dichloro-111-trifluoroethan-2b63/ |
|  | (Inhalation)  Cardiovascular, Dermal, Endocrine, Gastrointestinal, Hematologic, Hepatic, Immune, Metabolic, Musculoskeletal, Nervous, Ocular, Reproductive, Respiratory, Urinary, Whole Body | https://hawc.epa.gov/summary/data-pivot/assessment/100500347/17c-Figure-17c-22-Dichloro-111-trifluoroethan-fe2d/ |
|  | N/A | https://hawc.epa.gov/summary/visual/assessment/100500347/22-Dichloro-111-trifluoroethane-DTXSID7020712/ |
| **2,3,3,3-Tetrafluoropropene**  DTXSID4074728 | (Inhalation)  Developmental, Multi-System, Reproductive, Whole Body | https://hawc.epa.gov/summary/data-pivot/assessment/100500347/18a-Figure-18-2333-Tetrafluoropropene_Inhalation/ |
|  | (Inhalation)  Cardiovascular, Endocrine, Hematologic, Hepatic, Immune, Metabolic, Reproductive, Respiratory, Urinary, Whole Body, Cardiovascular, Dermal, Endocrine, Gastrointestinal, Musculoskeletal, Nervous, Ocular | https://hawc.epa.gov/summary/data-pivot/assessment/100500347/18b-Figure-18b-2333-Tetrafluoropropene_Inhala-826c/ |
|  | N/A | https://hawc.epa.gov/summary/visual/assessment/100500347/2333-Tetrafluoropropene-DTXSID4074728/ |
| **2-{[(Bicyclo[2.2.1]hept-5-en-2-yl)oxy]methyl}-1,1,1,3,3,3-hexafluoropropan-2-ol**  DTXSID50609108 | (Inhalation)  Hematologic, Respiratory, Whole Body | https://hawc.epa.gov/summary/data-pivot/assessment/100500347/19-Figure-19-2-Bicyclo221hept-5-en-2-yloxymet-fa8b/ |
|  | N/A | https://hawc.epa.gov/summary/visual/assessment/100500347/2-Bicyclo221hept-5-en-2-yloxymethyl-111333-he-03ec/ |
| **3,3-Dichloro-1,1,1,2,2-pentafluoropropane**  DTXSID1042027 | (Inhalation)  Cardiovascular, Dermal, Endocrine, Gastrointestinal, Hepatic, Immune, Metabolic, Musculoskeletal, Nervous, Ocular, Reproductive, Respiratory, Urinary, Whole Body, Auditory, Endocrine | https://hawc.epa.gov/summary/data-pivot/assessment/100500347/20-Figure-20-33-Dichloro-11122-pentafluoropro-4cbb/ |
|  | N/A | https://hawc.epa.gov/summary/visual/assessment/100500347/33-Dichloro-11122-pentafluoropropane-DTXSID1042027/ |
| **3-Trifluoromethylpyridine**  DTXSID10191397 | (Inhalation)  Cardiovascular, Dermal, Endocrine, Gastrointestinal, Hematologic, Hepatic, Immune, Musculoskeletal, Nervous, Ocular, Reproductive, Respiratory, Urinary, Whole Body | https://hawc.epa.gov/summary/data-pivot/assessment/100500347/21-Figure-21-3-Trifluoromethylpyridine_Inhalation/ |
|  | N/A | https://hawc.epa.gov/summary/visual/assessment/100500347/3-Trifluoromethylpyridine-DTXSID10191397/ |
| **Chlorodifluoromethane**  DTXSID6020301 | (Inhalation)  Cardiovascular, Dermal, Hematologic, Hepatic, Metabolic, Multi-System, Nervous, Ocular, Respiratory, Whole Body, Urinary, Hepatic, Endocrine, Immune, Musculoskeletal, Reproductive, Urinary, Whole Body | https://hawc.epa.gov/summary/data-pivot/assessment/100500347/22a-Figure-22a-Chlorodifluoromethane_Inhalation/ |
|  | (Inhalation)  Endocrine, Hepatic, Reproductive, Whole Body, Respiratory | https://hawc.epa.gov/summary/data-pivot/assessment/100500347/22b-Figure-22b-Chlorodifluoromethane_Inhalati-2466/ |
|  | N/A | https://hawc.epa.gov/summary/visual/assessment/100500347/Chlorodifluoromethane-DTXSID6020301/ |
| **Chloropentafluorobenzene**  DTXSID4059837 | (Inhalation)  Cardiovascular, Endocrine, Hepatic, Immune, Nervous, Reproductive, Respiratory, Urinary, Whole Body, Hematologic, Metabolic, | https://hawc.epa.gov/summary/data-pivot/assessment/100500347/23-Figure-23-Chloropentafluorobenzene-Short-t-ebf5/ |
|  | (Inhalation)  Cardiovascular, Endocrine, Hematologic, Hepatic, Immune, Metabolic, Nervous, Reproductive, Urinary, Whole Body | https://hawc.epa.gov/summary/data-pivot/assessment/100500347/23b-Figure-23b-Chloropentafluorobenzene-Subchronic/ |
|  | N/A | https://hawc.epa.gov/summary/visual/assessment/100500347/Chloropentafluorobenzene-DTXSID4059837/ |
| **Chlorotrifluoroethylene**  DTXSID3026485 | (Inhalation)  Developmental, Hematologic, Hepatic, Immune, Reproductive, Respiratory, Urinary, Whole Body | https://hawc.epa.gov/summary/data-pivot/assessment/100500347/24a-Figure-24a-Chloropentafluorobenzene-ReproDev/ |
|  | (Inhalation)  Cardiovascular, Hematologic, Hepatic, Immune, Metabolic, Nervous, Reproductive, Respiratory, Urinary, Whole Body | https://hawc.epa.gov/summary/data-pivot/assessment/100500347/24b-Figure-24b-Chloropentafluorobenzene-Subchronic/ |
|  | N/A | https://hawc.epa.gov/summary/visual/assessment/100500347/Chlorotrifluoroethylene-DTXSID3026485/ |
| **Dichlorodifluoromethane**  DTXSID6020436 | (Inhalation)  Cardiovascular, Hematologic, Haptic, Immune, Respiratory, Urinary, Whole Body, Dermal, Multi-System, Nervous, Ocular, Reproductive | https://hawc.epa.gov/summary/data-pivot/assessment/100500347/25a-Figure-25a-Dichlorodifluorometha_Inhalati-e148/ |
|  | (Inhalation)  Endocrine, Hepatic, Reproductive, Whole Body, Respiratory | https://hawc.epa.gov/summary/data-pivot/assessment/100500347/25b-Figure-25b-Dichlorodifluoromethane_Inhala-8eaa/ |
|  | N/A | https://hawc.epa.gov/summary/visual/assessment/100500347/Dichlorodifluoromethane-DTXSID6020436/ |
| **Difluoromethane**  DTXSID6029597 | (Inhalation)  Developmental, Reproductive, Whole Body | https://hawc.epa.gov/summary/data-pivot/assessment/100500347/26a-Figure-26a-Difluoromethane-ReproDev/ |
|  | (Inhalation)  Cardiovascular, Dermal, Endocrine, Gastrointestinal, Hematologic, Hepatic, Immune, Metabolic, Musculoskeletal, Nervous, Ocular, Reproductive, Respiratory, Urinary, Whole Body | https://hawc.epa.gov/summary/data-pivot/assessment/100500347/26b-Figure-26b-Difluoromethane-Subchronic/ |
|  | N/A | https://hawc.epa.gov/summary/visual/assessment/100500347/Difluoromethane-DTXSID6029597/ |
| **Hexafluoroacetone**  DTXSID9043778 | (Inhalation)  Dermal, Reproductive, Respiratory, Whole Body | https://hawc.epa.gov/summary/data-pivot/assessment/100500347/27-Figure-27-Hexafluoroacetone_Inhalation/ |
|  | N/A | https://hawc.epa.gov/summary/visual/assessment/100500347/Hexafluoroacetone-DTXSID9043778/ |
| **Methane, bromochlorodifluoro**  DTXSID0027147 | (Inhalation)  Developmental, Reproductive, Whole Body | https://hawc.epa.gov/summary/data-pivot/assessment/100500347/28-Figure-28-Methane-bromochlorodifluoro-Inha-7702/ |
|  | N/A | https://hawc.epa.gov/summary/visual/assessment/100500347/Methane-bromochlorodifluoro-DTXSID0027147/ |
| **Pentafluoroethane**  DTXSID1024251 | (Inhalation)  Developmental, Whole Body | https://hawc.epa.gov/summary/data-pivot/assessment/100500347/29a-Figure-29a-Pentafluoroethane-ReproDev/ |
|  | (Inhalation)  Cardiovascular, Dermal, Endocrine, Gastrointestinal, Hematologic, Hepatic, Immune, Metabolic, Musculoskeletal, Nervous, Ocular, Reproductive, Respiratory, Urinary, Whole Body, Endocrine, | https://hawc.epa.gov/summary/data-pivot/assessment/100500347/29b-Figure-29b-Pentafluoroethane-Short-termSu-0ab4/ |
|  | N/A | https://hawc.epa.gov/summary/visual/assessment/100500347/Pentafluoroethane-DTXSID1024251/ |
| **Perfluoroethane**  DTXSID2041915 | (Inhalation)  Developmental, Hematologic, Metabolic, Nervous, Reproductive, Urinary, Whole Body | https://hawc.epa.gov/summary/data-pivot/assessment/100500347/30a-Figure-30a-Pentafluoroethane-ReproDev/ |
|  | (Inhalation)  Cardiovascular, Endocrine, Hematologic, Metabolic, Multi-System, Nervous, Ocular, Reproductive, Respiratory, Urinary, Whole Body | https://hawc.epa.gov/summary/data-pivot/assessment/100500347/30a-Figure-30b-Perfluoroethane-Short-term/ |
|  | N/A | https://hawc.epa.gov/summary/visual/assessment/100500347/Perfluoroethane-DTXSID2041915/ |
| **Perfluoroethyl vinyl ether**  DTXSID1075305 | (Inhalation)  Cardiovascular, Dermal, Endocrine, Gastrointestinal, Hematologic, Hepatic, Immune, Metabolic, Musculoskeletal, Nervous, Ocular, Reproductive, Respiratory, Urinary, Whole Body | https://hawc.epa.gov/summary/data-pivot/assessment/100500347/31-Figure-31-Perfluoroethylvinylether/ |
|  | N/A | https://hawc.epa.gov/summary/visual/assessment/100500347/Perfluoroethyl-vinyl-ether-DTXSID1075305/ |
| **Tetrafluoroethylene**  DTXSID6021325 | (Inhalation)  Cardiovascular, Dermal, Developmental, Endocrine, Gastrointestinal, Hematologic, Hepatic, Immune, Musculoskeletal, Nervous, Ocular, Reproductive, Respiratory, Urinary, Whole Body | https://hawc.epa.gov/summary/data-pivot/assessment/100500347/32a-Figure-32a-Dichlorodifluoromethane_Inhala-484b/ |
|  | (Inhalation)  Cardiovascular, Dermal, Endocrine, Gatornationals, Hepatic, Immune, Multi-System, Musculoskeletal, Nervous, Reproductive, Respiratory, Urinary, Whole Body , Metabolic, Ocular | https://hawc.epa.gov/summary/data-pivot/assessment/100500347/32b-Figure-32b-Tetrafluoroethylene_Inhalation-2065/ |
|  | N/A | https://hawc.epa.gov/summary/visual/assessment/100500347/Tetrafluoroethylene-DTXSID6021325/ |
| **Trifluoro(trifluoromethyl)oxirane**  DTXSID6029177 | (Inhalation)  Developmental, Hematologic, Immune, Metabolic, Multi-System, Musculoskeletal, Nervous, Reproductive, Respiratory, Whole Body | https://hawc.epa.gov/summary/data-pivot/assessment/100500347/33a-Figure-33a-Trifluorotrifluoromethyloxiran-f17f/ |
|  | (Inhalation)  Reproductive, Respiratory, Urinary Hepatic, Whole Body | https://hawc.epa.gov/summary/data-pivot/assessment/100500347/33b-Figure-33b-Trifluorotrifluoromethyloxiran-920a/ |
|  | N/A | https://hawc.epa.gov/summary/visual/assessment/100500347/Trifluorotrifluoromethyloxirane-DTXSID6029177/ |
| **Trifluoroacetyl Chloride**  DTXSID1029176 | (Inhalation)  Cardiovascular, Endocrine, Hematologic, Hepatic, Immune, Metabolic, Musculoskeletal, Nervous, Ocular, Reproductive, Respiratory, Whole Body | https://hawc.epa.gov/summary/data-pivot/assessment/100500347/34-Figure-34-Trifluoroacetyl-chloride/ |
|  | N/A | https://hawc.epa.gov/summary/visual/assessment/100500347/Trifluoroacetyl-chloride-DTXSID1029176/ |
| **Trifluoroiodomethane**  DTXSID2062325 | (Inhalation)  Cardiovascular, Developmental, Endocrine, Hematologic, Hepatic, Immune, Metabolic, Musculoskeletal, Nervous, Reproductive, Respiratory, Urinary, Whole Body | https://hawc.epa.gov/summary/data-pivot/assessment/100500347/35a-Figure-35a-Trifluoroiodomethane_Inhalatio-c8ce/ |
|  | (Inhalation)  Endocrine, Hematologic, Hepatic, Whole Body, Cardiovascular, Endocrine, Gastrointestinal, Immune, Metabolic, Musculoskeletal, Nervous, Ocular, Reproductive, Respiratory, Urinary | https://hawc.epa.gov/summary/data-pivot/assessment/100500347/35b-Figure-35b-Trifluoroiodomethane_Inhalatio-51ed/ |
|  | N/A | https://hawc.epa.gov/summary/visual/assessment/100500347/Trifluoroiodomethane-DTXSID2062325/ |
| **Trifluoromethane**  DTXSID0026410 | (Inhalation)  Cardiovascular, Dermal, Hematologic, Hepatic, Metabolic, Multi-System, Nervous, Ocular, Respiratory, Urinary, Whole Body, Reproductive | https://hawc.epa.gov/summary/data-pivot/assessment/100500347/36-Figure-36-Trifluoromethane_Inhalation/ |
|  | N/A | https://hawc.epa.gov/summary/visual/assessment/100500347/Trifluoromethane-DTXSID0026410/ |
| **Vinylidene Fluoride**  DTXSID3021439 | (Inhalation)  Developmental, Reproductive, Whole Body | https://hawc.epa.gov/summary/data-pivot/assessment/100500347/37a-Figure-37a-Vinylidene-Fluoride_Inhalation-2219/ |
|  | (Inhalation)  Hematologic, Hepatic, Urinary, Whole Body | https://hawc.epa.gov/summary/data-pivot/assessment/100500347/37b-Figure-37b-Vinylidene-Fluoride_Inhalation-97db/ |
|  | (Inhalation)  Cardiovascular, Dermal, Endocrine, Gastrointestinal, Hematological, Hepatic, Immune, Musculoskeletal, Nervous, Ocular, Reproductive, Respiratory, Urinary, Whole Body | https://hawc.epa.gov/summary/data-pivot/assessment/100500347/37c-Figure-37c-Tetrafluoroethylene_Inhalation-dbaa/ |
|  | (Oral) Dermal, Endocrine, Gastrointestinal, Hepatic, Immune, Multi-System, Nervous, Reproductive, Respiratory, Urinary | https://hawc.epa.gov/summary/data-pivot/assessment/100500347/77-Figure-77-Vinylidene-fluoride_Oral/ |
|  | N/A | https://hawc.epa.gov/summary/visual/assessment/100500347/Vinylidene-fluoride-DTXSID3021439/ |
| **1-Propene, 1,1,2-trichloro-3,3,3-trifluoro**  DTXSID8073184 | (Oral)  Hepatic, Metabolic, Urinary, Whole Body | https://hawc.epa.gov/summary/data-pivot/assessment/100500347/52-Figure-52-1-Propene-112-trichloro-333-trif-a2f9/ |
|  | N/A | https://hawc.epa.gov/summary/visual/assessment/100500347/1-Propene-112-trichloro-333-trifluoro-DTXSID8-dab1/ |
| **2-(N-Methylperfluorobutylsulfonamido)ethyl acrylate**  DTXSID7070507 | (Oral)  Cardiovascular, Dermal, Developmental, Endocrine, Gastrointestinal, Hematologic, Hepatic, Immune, Metabolic, Musculoskeletal, Nervous, Ocular, Reproductive, Respiratory, Urinary, Whole Body | https://hawc.epa.gov/summary/data-pivot/assessment/100500347/53-Figure-53-2-N-Methylperfluorobutylsulfonam-25c6/ |
|  | N/A | https://hawc.epa.gov/summary/visual/assessment/100500347/2-N-Methylperfluorobutylsulfonamidoethyl-acry-13dc/ |
| **2-(N-Methylperfluorobutylsulfonamido)ethyl acrylate**  DTXSID7070507  **2,4,6-Trimethyl-2,4,6-tris(3,3,3-trifluoropropyl)cyclotrisiloxane**  DTXSID7051892 | (Oral)  Developmental, reproductive, Whole Body | https://hawc.epa.gov/summary/data-pivot/assessment/100500347/53b-Figure-53b-2-N-Methylperfluorobutylsulfon-beff/ |
| **2-(N-Methylperfluorobutylsulfonamido)ethyl acrylate**  DTXSID7070507  **2,4,6-Trimethyl-2,4,6-tris(3,3,3-trifluoropropyl)cyclotrisiloxane**  DTXSID7051892  **2-Chloro-1,1,1-trifluoroethane**  DTXSID5020289 | (Oral)  Cardiovascular, Hematologic, Hepatic, Metabolic, Musculoskeletal, Nervous, Ocular, Reproductive, Whole Body | https://hawc.epa.gov/summary/data-pivot/assessment/100500347/54-Figure-54-246-Trimethyl-246-tris333-triflu-ae50/ |
|  | (Oral)  Dermal, Endocrine, Hepatic, Immune, Nervous, Reproductive, Respiratory, Urinary, Whole Body | https://hawc.epa.gov/summary/data-pivot/assessment/100500347/55-Figure-55-2-Chloro-111-trifluoroethaneOral/ |
| **3,4,5-Trifluorophenol**  DTXSID00380718 | (Oral)  Cardiovascular, Dermal, Endocrine, Gastrointestinal, Hematologic, Hepatic, Immune, Metabolic, Musculoskeletal, Nervous, Ocular, Reproductive, Respiratory, Urinary, Whole Body | https://hawc.epa.gov/summary/data-pivot/assessment/100500347/56-Figure-56-345-Trifluorophenol_Oral/ |
|  | N/A | https://hawc.epa.gov/summary/visual/assessment/100500347/345-Trifluorophenol-DTXSID00380718/ |
| **4-(Perfluoro(2,2-bis(1-methylethyl)(1-methyl)ethen-1-yloxy))benzenesulfonic acid sodium salt**  DTXSID601020833 | (Oral)  Developmental, Endocrine, Metabolic, Reproductive, Gastrointestinal, Metabolic | https://hawc.epa.gov/summary/data-pivot/assessment/100500347/57-Figure-57-4-Perfluoro22-bis1-methylethyl1--8bb1/ |
|  | N/A | https://hawc.epa.gov/summary/visual/assessment/100500347/4-Perfluoro22-bis1-methylethyl1-methylethen-1-dffa/ |
| **4-(Perfluoro(2,2-bis(1-methylethyl)(1-methyl)ethen-1-yloxy))benzenesulfonic acid sodium salt**  DTXSID601020833  **5-(1,2,2,2-Tetrafluoro)ethoxy-perfluoro-3-oxa-4-methylpentanesulfonic acid**  DTXSID10892352 | (Oral)  Gastrointestinal, Hepatic, Metabolic, Reproductive, Whole Body | https://hawc.epa.gov/summary/data-pivot/assessment/100500347/57b-Figure-57b-4-Perfluoro22-bis1-methylethyl-def5/ |
| **4-(Perfluoro(2,2-bis(1-methylethyl)(1-methyl)ethen-1-yloxy))benzenesulfonic acid sodium salt**  DTXSID601020833  **5-(1,2,2,2-Tetrafluoro)ethoxy-perfluoro-3-oxa-4-methylpentanesulfonic acid**  DTXSID10892352  **5-(1,2,2,2-Tetrafluoro)ethoxy-perfluoro-3-oxa-4-methylpentanesulfonic acid**  DTXSID10892352  **Bisphenol AF**  DTXSID7037717 | (Oral)  Cardiovascular, Developmental, Endocrine, Hepatic, Immune, Metabolic, Musculoskeletal, Reproductive, Urinary, Whole Body | https://hawc.epa.gov/summary/data-pivot/assessment/100500347/58a-Figure-58a-5-1222-Tetrafluoroethoxy-perfluo/ |
|  | (Oral)  Hepatic, Metabolic, Whole Body | https://hawc.epa.gov/summary/data-pivot/assessment/100500347/58b-Figure-58b-5-1222-Tetrafluoroethoxy-perfl-a2f1/ |
| **5-(1,2,2,2-Tetrafluoro)ethoxy-perfluoro-3-oxa-4-methylpentanesulfonic acid**  DTXSID10892352  **Bisphenol AF**  DTXSID7037717  **Bisphenol AF**  DTXSID7037717  **Chlorodifluoromethane**  DTXSID6020301 | (Oral)  Developmental, Reproductive, Whole Body, Cardiovascular, Dermal, Developmental, Endocrine, Gastrointestinal, Hematologic, Hepatic, Immune, Metabolic, Musculoskeletal, Nervous, Ocular, Reproductive, Respiratory, Urinary, Whole Body | https://hawc.epa.gov/summary/data-pivot/assessment/100500347/59a-Figure-59a-Bisphenol-AF_Oral-NTP-2022-11152721/ |
|  | (Oral)  Cardiovascular, Endocrine, Hematologic, Hepatic, Immune, Metabolic, Nervous, Reproductive, Respiratory, Urinary, Whole Body, Developmental | https://hawc.epa.gov/summary/data-pivot/assessment/100500347/59b-Figure-59b-Bisphenol-AF_Oral-ECHA-2023-11-98fd/ |
| **Bisphenol AF**  DTXSID7037717  **Chlorodifluoromethane**  DTXSID6020301  **Chlorotrifluoroethylene**  DTXSID3026485 | (Oral)  Developmental, Reproductive, Whole Body, Endocrine, | https://hawc.epa.gov/summary/data-pivot/assessment/100500347/59c-1-Figure-59c-Bisphenol-AF_Oral-Other-Dev-Ti-/ |
|  | (Oral)  Developmental, Reproductive, Whole Body, Endocrine, Hepatic, Immune, Metabolic, Nervous | https://hawc.epa.gov/summary/data-pivot/assessment/100500347/59c-2-Figure-59c-Bisphenol-AF_Oral-Other-Dev/ |
|  | (Oral)  Endocrine, Reproductive, Metabolic, Whole Body, Nervous, Cardiovascular, Gastrointestinal, Hematologic, Hepatic, Immune, Ocular, Urinary | https://hawc.epa.gov/summary/data-pivot/assessment/100500347/59d-Figure-59d-Bisphenol-AF_Oral-SubchronicShort/ |
|  | (Oral)  Endocrine, Metabolic, Reproductive | https://hawc.epa.gov/summary/data-pivot/assessment/100500347/59e-1-Figure-59e-Bisphenol-AF_Oral-Endocrine-Me/ |
|  | (Oral)  Endocrine, Metabolic, Reproductive | https://hawc.epa.gov/summary/data-pivot/assessment/100500347/59e2-Figure-59e2-Bisphenol-AF_RAT_Oral-Endocr-2234/ |
|  | (Oral)  Dermal, Endocrine, Hepatic, Immune, Nervous, Reproductive, Respiratory, Urinary, Whole Body | https://hawc.epa.gov/summary/data-pivot/assessment/100500347/60-Figure-60-Chlorodifluoromethane_Oral/ |
|  | (Oral)  Cardiovascular, Endocrine, Gastrointestinal, Hematologic, Hepatic, Immune, Metabolic, Multi-System, Musculoskeletal, Nervous, Reproductive, Urinary, Whole Body | https://hawc.epa.gov/summary/data-pivot/assessment/100500347/61-Figure-61-Chlorotrifluoroethylene_Oral/ |
| **Flubendiamide**  DTXSID4047672 | (Oral)  Endocrine, Reproductive, Whole Body | https://hawc.epa.gov/summary/data-pivot/assessment/100500347/60-Figure-61-Flubendiamide_Oral/ |
|  | N/A | https://hawc.epa.gov/summary/visual/assessment/100500347/Flubendiamide-DTXSID4047672/ |
|  |  |  |
|  |  |  |
| **N-(4-(1,1,1,3,3,3-Hexafluoro-2-hydroxy-propan-2-yl)phenyl)-N-(2,2,2-trifluoroethyl)benzenesulfonamide**  DTXSID6040618 | (Oral)  Hepatic, Metabolic, Whole Body, Immune, Respiratory, Nervous, Whole Body | https://hawc.epa.gov/summary/data-pivot/assessment/100500347/64-Figure-64-N-4-111333-Hexafluoro-2-hydroxy--e3a1/ |
|  | N/A | https://hawc.epa.gov/summary/visual/assessment/100500347/N-4-111333-Hexafluoro-2-hydroxy-propan-2-ylph-9bba/ |
| **N-[2,5-dichloro-4-(1,1,2,3,3,3-hexafluoropropoxy)-phenyl-aminocarbonyl]-2,6-difluorobenzamide**  DTXSID5034357 | (Oral)  Developmental, Hepatic, Reproductive, Urinary, Whole Body | https://hawc.epa.gov/summary/data-pivot/assessment/100500347/65-Figure-65-N-4-111333-Hexafluoro-2-hydroxy--1dc6/ |
|  | N/A | https://hawc.epa.gov/summary/visual/assessment/100500347/N-25-dichloro-4-112333-hexafluoropropoxy-phen-5874/ |
| **N-[2,5-dichloro-4-(1,1,2,3,3,3-hexafluoropropoxy)-phenyl-aminocarbonyl]-2,6-difluorobenzamide**  DTXSID5034357  **N-Nitroso(2,2,2-trifluoroethyl) ethylamine; N-Nitroso-bis-(4,4,4-trifluoro-N-butyl)amine**  DTXSID6020987 | (Oral)  Hepatic, Urinary, Hematologic, Metabolic | https://hawc.epa.gov/summary/data-pivot/assessment/100500347/65b-Figure-65b-N-25-dichloro-4-112333-hexaflu-ccec/ |
| **N-[2,5-dichloro-4-(1,1,2,3,3,3-hexafluoropropoxy)-phenyl-aminocarbonyl]-2,6-difluorobenzamide**  DTXSID5034357  **N-Nitroso(2,2,2-trifluoroethyl) ethylamine; N-Nitroso-bis-(4,4,4-trifluoro-N-butyl)amine**  DTXSID6020987  **Perfluoro-2-methoxyacetic acid**  DTXSID00408562 | (Oral)  Endocrine, Reproductive, Whole Body | https://hawc.epa.gov/summary/data-pivot/assessment/100500347/65b2-Figure-65b-N-25-dichloro-4-112333-hexafl-18c5/ |
|  | (Oral)  Hepatic, Respiratory, Whole Body, Gastrointestinal | https://hawc.epa.gov/summary/data-pivot/assessment/100500347/66-Figure-66-N-Nitroso222-trifluoroethyl-ethy-8503/ |
|  | (Oral)  Cardiovascular, Hepatic, Immune, Nervous, Urinary, Whole Body | https://hawc.epa.gov/summary/data-pivot/assessment/100500347/67-Figure-67-Perfluoro-2-methoxyacetic-acid_Oral/ |
| **Perfluoro-3,5,7,9,11-pentaoxadodecanoic acid**  DTXSID50723994 | (Oral)  Hepatic, Immune, Metabolic, Reproductive, urinary, Whole Body | https://hawc.epa.gov/summary/data-pivot/assessment/100500347/68-Figure-68-Perfluoro-357911-pentaoxadodecan-8c8b/ |
|  | N/A | https://hawc.epa.gov/summary/visual/assessment/100500347/Perfluoro-357911-pentaoxadodecanoic-acid-DTXS-e22e/ |
| **Perfluoro-3,5,7,9-butaoxadecanoic acid**  DTXSID90723993 | (Oral)  Hepatic, Metabolic, Nervous, Whole Body | https://hawc.epa.gov/summary/data-pivot/assessment/100500347/69a-Figure-69a-Perfluoro-3579-butaoxadecanoic-a/ |
|  | N/A | https://hawc.epa.gov/summary/visual/assessment/100500347/Perfluoro-3579-butaoxadecanoic-acid-DTXSID90723993/ |
| **Perfluoro-3,5,7,9-butaoxadecanoic acid**  DTXSID90723993  **Perfluoro-3,5,7-trioxaoctanoic acid**  DTXSID20892348 | (Oral)  Hepatic, Immune, Metabolic, Reproductive, Urinary, Whole Body | https://hawc.epa.gov/summary/data-pivot/assessment/100500347/69b-Figure-69b-Perfluoro-3579-butaoxadecanoic-f9bd/ |
| **Perfluoro-3,5,7,9-butaoxadecanoic acid**  DTXSID90723993  **Perfluoro-3,5,7-trioxaoctanoic acid**  DTXSID20892348  **Perfluoro-3,5-dioxahexanoic acid**  DTXSID50892351 | (Oral)  Hepatic, Metabolic, Whole Body | https://hawc.epa.gov/summary/data-pivot/assessment/100500347/70-Figure-70-Perfluoro-357-trioxaoctanoic-aci-0346/ |
|  | (Oral)  Hepatic, Metabolic, Whole Body | https://hawc.epa.gov/summary/data-pivot/assessment/100500347/71-Figure-71-Perfluoro-35-dioxahexanoic-acid_Oral/ |
| **Perfluorooctadecanoic** **acid**  DTXSID1066071 | (Oral)  Cardiovascular, Developmental, Endocrine, Gastrointestinal, Hematologic, Hepatic, Immune, Metabolic, Musculoskeletal, Nervous, Ocular, Reproductive, Respiratory, Urinary, Whole Body | https://hawc.epa.gov/summary/data-pivot/assessment/100500347/72-Figure-72-Perfluorooctadecanoic-acid_Oral/ |
|  | N/A | https://hawc.epa.gov/summary/visual/assessment/100500347/Perfluorooctadecanoic-acid-DTXSID1066071/ |
| **Potassium 9-chlorohexadecafluoro-3-oxanonane-1-sulfonate**  DTXSID60881236 | (Oral)  Cardiovascular, Endocrine, Hematologic, Hepatic, Immune, Metabolic, Nervous, Reproductive, Urinary, Whole Body | https://hawc.epa.gov/summary/data-pivot/assessment/100500347/73a-Figure-73a-Potassium-9-chlorohexadecafluoro/ |
|  | N/A | https://hawc.epa.gov/summary/visual/assessment/100500347/Potassium-9-chlorohexadecafluoro-3-oxanonane--5c32/ |
| **Potassium 9-chlorohexadecafluoro-3-oxanonane-1-sulfonate**  DTXSID60881236  **Pyrifluquinazon**  DTXSID6058057 | (Oral)  Gastrointestinal, Immune | https://hawc.epa.gov/summary/data-pivot/assessment/100500347/73b-Figure-73b-Potassium-9-chlorohexadecafluo-2acc/ |
| **Potassium 9-chlorohexadecafluoro-3-oxanonane-1-sulfonate**  DTXSID60881236  **Pyrifluquinazon**  DTXSID6058057  **Sodium trifluoroacetate**  DTXSID0062715 | (Oral)  Developmental, Reproductive, Whole Body | https://hawc.epa.gov/summary/data-pivot/assessment/100500347/74-Figure-74-Pyrifluquinazon_Oral/ |
|  | (Oral)  Metabolic, Whole Body | https://hawc.epa.gov/summary/data-pivot/assessment/100500347/75-Figure-75-Sodium-trifluoroacetate_Oral/ |
| **Tetrafluoro-m-phenylenediamine**  DTXSID1043752 | (Oral)  Cardiovascular, Endocrine, Gastrointestinal, Hepatic, Immune, Reproductive, Respiratory, Urinary, Whole Body | https://hawc.epa.gov/summary/data-pivot/assessment/100500347/76-Figure-76-Tetrafluoro-m-phenylenediamine_Oral/ |
|  | N/A | https://hawc.epa.gov/summary/visual/assessment/100500347/Tetrafluoro-m-phenylenediamine-DTXSID1043752/ |
| **Study Evaluation Summary (nonECHA)** | N/A | https://hawc.epa.gov/summary/visual/assessment/100500347/nonECHA_SQE/ |
| **Study Evaluation Summary (ECHA)** | N/A | https://hawc.epa.gov/summary/visual/assessment/100500347/ECHA_SQE/ |
| **(+)-(5Z,7E)-26,26,26,27,27,27-Hexafluoro-9,10-secocholesta-5,7,10(19)-triene-1alpha,3beta,25-triol**  DTXSID601027560 | N/A | https://hawc.epa.gov/summary/visual/assessment/100500347/5Z7E-262626272727-Hexafluoro-910-secocholesta-c45b/ |
| **(1RS,2RS)-2-(4-Hydroxyphenyl)-2-methyl-1-(9-(4,4,5,5,5-pentafluoropentyl)sulfinylnonyl)-1,2,3,4-tetrahydronaphth-6-ol**  DTXSID5044071 | N/A | https://hawc.epa.gov/summary/visual/assessment/100500347/1RS2RS-2-4-Hydroxyphenyl-2-methyl-1-9-44555-p-5842/ |
| **(2,2,2-Trifluoroethoxy)ethene**  DTXSID5059957 | N/A | https://hawc.epa.gov/summary/visual/assessment/100500347/222-Trifluoroethoxyethene-DTXSID5059957/ |
| **(E)-1,3,3,3-Tetrafluoro-1-propene**  DTXSID10885446 | N/A | https://hawc.epa.gov/summary/visual/assessment/100500347/E-1333-Tetrafluoro-1-propene-DTXSID10885446/ |
| **(Perfluorohexyl)ethyl acrylate**  DTXSID9038840 | N/A | https://hawc.epa.gov/summary/visual/assessment/100500347/Perfluorohexylethyl-acrylate-DTXSID9038840/ |
| **(Perfluorohexyl)ethylene**  DTXSID8067076 | N/A | https://hawc.epa.gov/summary/visual/assessment/100500347/Perfluorohexylethylene-DTXSID8067076/ |
|  |  |  |
| **1,1,1,3,3,3-Hexafluoro-2-chloropropane**  DTXSID4075033 | N/A | https://hawc.epa.gov/summary/visual/assessment/100500347/111333-Hexafluoro-2-chloropropane-DTXSID4075033/ |
| **1,1,1,4,4,4-Hexafluorobut-2-ene**  DTXSID90985276 | N/A | https://hawc.epa.gov/summary/visual/assessment/100500347/111444-Hexafluorobut-2-ene-DTXSID90985276/ |
| **1,1,1-Trichloro-2,2,2-trifluoroethane**  DTXSID5027148 | N/A | https://hawc.epa.gov/summary/visual/assessment/100500347/111-Trichloro-222-trifluoroethane-DTXSID5027148/ |
| **1,1,1-Trifluoro-N-[(trifluoromethyl)sulfonyl]methanesulfonamide**  DTXSID2045026 | N/A | https://hawc.epa.gov/summary/visual/assessment/100500347/111-Trifluoro-N-trifluoromethylsulfonylmethan-498a/ |
| **1,1,2-Trifluoroethane**  DTXSID8073182 | N/A | https://hawc.epa.gov/summary/visual/assessment/100500347/112-Trifluoroethane-DTXSID8073182/ |
| **1,1-Dichlorotetrafluoroethane**  DTXSID9027150 | N/A | https://hawc.epa.gov/summary/visual/assessment/100500347/11-Dichlorotetrafluoroethane-DTXSID9027150/ |
| **1,2-Dibromotetrafluoroethane**  DTXSID0041226 | N/A | https://hawc.epa.gov/summary/visual/assessment/100500347/12-Dibromotetrafluoroethane-DTXSID0041226/ |
| **1,2-Dichloro-1,1-difluoroethane**  DTXSID5031393 | N/A | https://hawc.epa.gov/summary/visual/assessment/100500347/12-Dichloro-11-difluoroethane-DTXSID5031393/ |
| **1,2-Difluoroethane**  DTXSID3073214 | N/A | https://hawc.epa.gov/summary/visual/assessment/100500347/12-Difluoroethane-DTXSID3073214/ |
| **1-bromo-1,2,2-trifluorocyclobutane**  DTXSID10959232 | N/A | https://hawc.epa.gov/summary/visual/assessment/100500347/1-bromo-122-trifluorocyclobutane-DTXSID10959232/ |
| **1-Bromo-3,4,5-trifluorobenzene**  DTXSID70346346 | N/A | https://hawc.epa.gov/summary/visual/assessment/100500347/1-Bromo-345-trifluorobenzene-DTXSID70346346/ |
| **1-Bromoheptadecafluorooctane**  DTXSID5046560 | N/A | https://hawc.epa.gov/summary/visual/assessment/100500347/1-Bromoheptadecafluorooctane-DTXSID5046560/ |
| **1-Chloro-1,2-difluoroethane**  DTXSID70861882 | N/A | https://hawc.epa.gov/summary/visual/assessment/100500347/1-Chloro-12-difluoroethane-DTXSID70861882/ |
| **1-Propene, 2-bromo-3,3,3-trifluoro-**  DTXSID80883575 | N/A | https://hawc.epa.gov/summary/visual/assessment/100500347/1-Propene-2-bromo-333-trifluoro-DTXSID80883575/ |
| **2,2,2-Trifluoroethanol**  DTXSID0021751 | N/A | https://hawc.epa.gov/summary/visual/assessment/100500347/222-Trifluoroethanol-DTXSID0021751/ |
| **2,2,2-Trifluoroethyl allyl ether**  DTXSID70165014 | N/A | https://hawc.epa.gov/summary/visual/assessment/100500347/222-Trifluoroethyl-allyl-ether-DTXSID70165014/ |
| **2,2,2-Trifluoroethyl ethyl ether**  DTXSID00196715 | N/A | https://hawc.epa.gov/summary/visual/assessment/100500347/222-Trifluoroethyl-ethyl-ether-DTXSID00196715/ |
| **2,2-Bis(trifluoromethyl)-1,3-dioxolane**  DTXSID2073275 | N/A | https://hawc.epa.gov/summary/visual/assessment/100500347/22-Bistrifluoromethyl-13-dioxolane-DTXSID2073275/ |
| **2,3,3,3-Tetrafluoro-2-(trifluoromethyl)propanenitrile**  DTXSID90505110 | N/A | https://hawc.epa.gov/summary/visual/assessment/100500347/2333-Tetrafluoro-2-trifluoromethylpropanenitr-61b5/ |
| **2,3,5,6-Tetrafluorobenzyl alcohol**  DTXSID20370090 | N/A | https://hawc.epa.gov/summary/visual/assessment/100500347/2356-Tetrafluorobenzyl-alcohol-DTXSID20370090/ |
| **2,4,6-Trimethyl-2,4,6-tris(3,3,3-trifluoropropyl)cyclotrisiloxane**  DTXSID7051892 | N/A | https://hawc.epa.gov/summary/visual/assessment/100500347/246-Trimethyl-246-tris333-trifluoropropylcycl-3ded/ |
| **2-(6-Fluoro-1H-indol-3-yl)-N-[[3-(2,2,3,3-tetrafluoropropoxy)phenyl]methyl]ethanamine**  DTXSID201026015 | N/A | https://hawc.epa.gov/summary/visual/assessment/100500347/2-6-Fluoro-1H-indol-3-yl-N-3-2233-tetrafluoro-4032/ |
| **2-(N-(Perfluorobutylsulfonyl)-N-methylamino)ethanol**  **DTXSID0067848** | N/A | https://hawc.epa.gov/summary/visual/assessment/100500347/2-N-Perfluorobutylsulfonyl-N-methylaminoethan-3dff/ |
| **2-(N-Methylperfluorobutanesulfonamido)ethyl methacrylate**  DTXSID6070510 | N/A | https://hawc.epa.gov/summary/visual/assessment/100500347/2-N-Methylperfluorobutanesulfonamidoethyl-met-6292/ |
| **2-(Perfluorodecyl)ethanol**  DTXSID2029905 | N/A | https://hawc.epa.gov/summary/visual/assessment/100500347/2-Perfluorodecylethanol-DTXSID2029905/ |
| **2-Bromo-1,1,1,2-tetrafluoroethane**  DTXSID10861765 | N/A | https://hawc.epa.gov/summary/visual/assessment/100500347/2-Bromo-1112-tetrafluoroethane-DTXSID10861765/ |
| **2-Bromo-1,1,1-trifluoroethane**  DTXSID2074324 | N/A | https://hawc.epa.gov/summary/visual/assessment/100500347/2-Bromo-111-trifluoroethane-DTXSID2074324/ |
| **2-Chloro-1,1,1-trifluoroethane**  DTXSID5020289 | N/A | https://hawc.epa.gov/summary/visual/assessment/100500347/2-Chloro-111-trifluoroethane-DTXSID5020289/ |
| **2-chloro-2,2-difluoroethanol**  DTXSID30397556 | N/A | https://hawc.epa.gov/summary/visual/assessment/100500347/2-chloro-22-difluoroethanol-DTXSID30397556/ |
| **2-Propenoic acid, 2-methyl-, 2,2,2-trifluoro-1-(trifluoromethyl)ethyl ester**  DTXSID50883729 | N/A | https://hawc.epa.gov/summary/visual/assessment/100500347/2-Propenoic-acid-2-methyl-222-trifluoro-1-tri-2ad7/ |
| **2-Propyl-4-pentafluoroethyl-1-((2'-(1H-tetrazol-5-yl)biphenyl-4-yl)methyl)imidazole-5-carboxylic acid**  DTXSID00154476 | N/A | https://hawc.epa.gov/summary/visual/assessment/100500347/2-Propyl-4-pentafluoroethyl-1-2-1H-tetrazol-5-6697/ |
| **2H-Perfluoropropane**  DTXSID4042048 | N/A | https://hawc.epa.gov/summary/visual/assessment/100500347/2H-Perfluoropropane-DTXSID4042048/ |
| **3,3,3-Trifluoro-1-propene**  DTXSID9041287 | N/A | https://hawc.epa.gov/summary/visual/assessment/100500347/333-Trifluoro-1-propene-DTXSID9041287/ |
| **3,3,3-Trifluoropropionic acid**  DTXSID70380740 | N/A | https://hawc.epa.gov/summary/visual/assessment/100500347/333-Trifluoropropionic-acid-DTXSID70380740/ |
| **3,3,4,4,4-Pentafluorobut-1-ene**  **DTXSID80190837** | N/A | https://hawc.epa.gov/summary/visual/assessment/100500347/33444-Pentafluorobut-1-ene-DTXSID80190837/ |
| **3,3,4,4,5,5,6,6,7,7,8,8,8-Tridecafluorooctane-1-sulfonate**  DTXSID40873417 | N/A | https://hawc.epa.gov/summary/visual/assessment/100500347/3344556677888-Tridecafluorooctane-1-sulfonate-fbe1/ |
| **3,3,4,4,5,5,6,6,7,7,8,8,9,9,10,10,10-Heptadecafluorodecane-1-sulfonate**  DTXSID80873416 | N/A | https://hawc.epa.gov/summary/visual/assessment/100500347/33445566778899101010-Heptadecafluorodecane-1--800a/ |
| **4,5-Difluoro-2,2-bis(trifluoromethyl)-1,3-dioxole**  DTXSID6073493 | N/A | https://hawc.epa.gov/summary/visual/assessment/100500347/45-Difluoro-22-bistrifluoromethyl-13-dioxole--b400/ |
| **4:2 Fluorotelomer sulfonate**  DTXSID90891565 | N/A | https://hawc.epa.gov/summary/visual/assessment/100500347/42-Fluorotelomer-sulfonate-DTXSID90891565/ |
| **5-(1,2,2,2-Tetrafluoro)ethoxy-perfluoro-3-oxa-4-methylpentanesulfonic acid**  DTXSID10892352 | N/A | https://hawc.epa.gov/summary/visual/assessment/100500347/5-1222-Tetrafluoroethoxy-perfluoro-3-oxa-4-me-b1c5/ |
| **6:2 Fluorotelomer sulfonamide betaine**  DTXSID4041284 | N/A | https://hawc.epa.gov/summary/visual/assessment/100500347/62-Fluorotelomer-sulfonamide-betaine-DTXSID4041284/ |
| **6:2 Fluorotelomer sulfonate sodium salt**  DTXSID50896637 | N/A | https://hawc.epa.gov/summary/visual/assessment/100500347/62-Fluorotelomer-sulfonate-sodium-salt-DTXSID-12ba/ |
| **6:2 Fluorotelomer thiohydroxy ammonium chloride**  DTXSID50892533 | N/A | https://hawc.epa.gov/summary/visual/assessment/100500347/62-Fluorotelomer-thiohydroxy-ammonium-chlorid-d3fe/ |
| **Ammonium 2-(2-(2-(aminosulphonyl)-1,1,2,2-tetrafluoroethoxy)-1,1,2,3,3,3-hexafluoropropoxy)-2,3,3,3-tetrafluoropropionate**  DTXSID00880203 | N/A | https://hawc.epa.gov/summary/visual/assessment/100500347/Ammonium-2-2-2-aminosulphonyl-1122-tetrafluor-d6f4/ |
| **Ammonium perfluoro[(5-methoxy-1,3-dioxolan-4-yl)oxy]acetate**  DTXSID00882626 | N/A | https://hawc.epa.gov/summary/visual/assessment/100500347/Ammonium-perfluoro5-methoxy-13-dioxolan-4-ylo-253d/ |
|  |  |  |
|  |  |  |
| **Bisphenol AF**  DTXSID7037717 | N/A | https://hawc.epa.gov/summary/visual/assessment/100500347/Bisphenol-AF-DTXSID7037717/ |
| **Broflanilide**  DTXSID50894815 | N/A | https://hawc.epa.gov/summary/visual/assessment/100500347/Broflanilide-DTXSID50894815/ |
| **Bromodifluoromethane**  DTXSID5061740 | N/A | https://hawc.epa.gov/summary/visual/assessment/100500347/Bromodifluoromethane-DTXSID5061740/ |
| **Bromotrifluoroethene**  DTXSID0060513 | N/A | https://hawc.epa.gov/summary/visual/assessment/100500347/Bromotrifluoroethene-DTXSID0060513/ |
| **Bromotrifluoromethane**  DTXSID5026415 | N/A | https://hawc.epa.gov/summary/visual/assessment/100500347/Bromotrifluoromethane-DTXSID5026415/ |
| **Carbon tetrafluoride**  DTXSID2041757 | N/A | https://hawc.epa.gov/summary/visual/assessment/100500347/Carbon-tetrafluoride-DTXSID2041757/ |
| **Carbonic difluoride**  DTXSID7059858 | N/A | https://hawc.epa.gov/summary/visual/assessment/100500347/Carbonic-difluoride-DTXSID7059858/ |
| **Chlorodifluoromethane and Chloropentafluoroethane**  DTXSID50192601 | N/A | https://hawc.epa.gov/summary/visual/assessment/100500347/Chlorodifluoromethane-and-Chloropentafluoroet-6a4b/ |
| **Chloropentafluoroethane**  DTXSID3026435 | N/A | https://hawc.epa.gov/summary/visual/assessment/100500347/Chloropentafluoroethane-DTXSID3026435/ |
| **Chlorotrifluoromethane**  DTXSID4052500 | N/A | https://hawc.epa.gov/summary/visual/assessment/100500347/Chlorotrifluoromethane-DTXSID4052500/ |
| **cis-Perfluorodecahydronaphthalene**  DTXSID801021518 | N/  A | https://hawc.epa.gov/summary/visual/assessment/100500347/cis-Perfluorodecahydronaphthalene-DTXSID801021518/ |
| **Cyanuric fluoride**  DTXSID6060975 | N/A | https://hawc.epa.gov/summary/visual/assessment/100500347/Cyanuric-fluoride-DTXSID6060975/ |
| **Dibromodifluoromethane**  DTXSID9058789 | N/A | https://hawc.epa.gov/summary/visual/assessment/100500347/Dibromodifluoromethane-DTXSID9058789/ |
| **Difluoroacetate**  DTXSID00394097 | N/A | https://hawc.epa.gov/summary/visual/assessment/100500347/difluoroacetate-DTXSID00394097/ |
| **Difluoroacetic acid**  DTXSID2059932 | N/A | https://hawc.epa.gov/summary/visual/assessment/100500347/Difluoroacetic-acid-DTXSID2059932/ |
| **Ethene, 1,1-dichloro-2,2-difluoro-**  DTXSID6073150 | N/A | https://hawc.epa.gov/summary/visual/assessment/100500347/Ethene-11-dichloro-22-difluoro-DTXSID6073150/ |
| **Ethene, trifluoro-**  DTXSID4059887 | N/A | https://hawc.epa.gov/summary/visual/assessment/100500347/Ethene-trifluoro-DTXSID4059887/ |
| **Ethyl trifluoroacetate**  DTXSID8041959 | N/A | https://hawc.epa.gov/summary/visual/assessment/100500347/Ethyl-trifluoroacetate-DTXSID8041959/ |
| **Ethylamine, 2,2-difluoro-**  **DTXSID40195621** | N/A | https://hawc.epa.gov/summary/visual/assessment/100500347/Ethylamine-22-difluoro-DTXSID40195621/ |
| **Fulvestrant**  DTXSID4022369 | N/A | https://hawc.epa.gov/summary/visual/assessment/100500347/Fulvestrant-DTXSID4022369/ |
| **Hexafluoroacetone sesquihydrate**  DTXSID7025392 | N/A | https://hawc.epa.gov/summary/visual/assessment/100500347/Hexafluoroacetone-sesquihydrate-DTXSID7025392/ |
| **Hexafluorobenzene**  DTXSID5043924 | N/A | https://hawc.epa.gov/summary/visual/assessment/100500347/Hexafluorobenzene-DTXSID5043924/ |
| **Hexafluorocyclobutene**  DTXSID2073229 | N/A | https://hawc.epa.gov/summary/visual/assessment/100500347/Hexafluorocyclobutene-DTXSID2073229/ |
| **Hexafluoropropene**  DTXSID2026949 | N/A | https://hawc.epa.gov/summary/visual/assessment/100500347/Hexafluoropropene-DTXSID2026949/ |
| **Isopropyl trifluoroacetate**  DTXSID80193060 | N/A | https://hawc.epa.gov/summary/visual/assessment/100500347/Isopropyl-trifluoroacetate-DTXSID80193060/ |
| **Methyl 2,2,3-trifluoro-3-oxopropanoate**  DTXSID8052437 | N/A | https://hawc.epa.gov/summary/visual/assessment/100500347/Methyl-223-trifluoro-3-oxopropanoate-DTXSID8052437/ |
| **Methyl trifluoroacetate**  DTXSID2059988 | N/A | https://hawc.epa.gov/summary/visual/assessment/100500347/Methyl-trifluoroacetate-DTXSID2059988/ |
| **Methyl trifluoromethanesulfonate**  DTXSID6049272 | N/A | https://hawc.epa.gov/summary/visual/assessment/100500347/Methyl-trifluoromethanesulfonate-DTXSID6049272/ |
| **Midaflur**  DTXSID50178425 | N/A | https://hawc.epa.gov/summary/visual/assessment/100500347/Midaflur-DTXSID50178425/ |
| **N,N-Bis(2-hydroxyethyl)perfluorobutanesulfonamide**  DTXSID50188029 | N/A | https://hawc.epa.gov/summary/visual/assessment/100500347/NN-Bis2-hydroxyethylperfluorobutanesulfonamid-ed9f/ |
| **N-((7S)-6,7-Dihydro-6-oxo-5H-dibenz(b,d)azepin-7-yl)-2,2-dimethyl-N'-(2,2,3,3,3-pentafluoropropyl)-propanediamide**  DTXSID2023383 | N/A | https://hawc.epa.gov/summary/visual/assessment/100500347/N-7S-67-Dihydro-6-oxo-5H-dibenzbdazepin-7-yl--1511/ |
| **N-Nitroso(2,2,2-trifluoroethyl) ethylamine**  DTXSID3021011 | N/A | https://hawc.epa.gov/summary/visual/assessment/100500347/N-Nitroso222-trifluoroethyl-ethylamine-DTXSID-e1d7/ |
| **N-Nitroso-bis-(4,4,4-trifluoro-N-butyl)amine**  **DTXSID6020987** | N/A | https://hawc.epa.gov/summary/visual/assessment/100500347/N-Nitroso-bis-444-trifluoro-N-butylamine-DTXS-d108/ |
| **N-Trifluoroacetyl-2-aminoethanol**  DTXSID10220035 | N/A | https://hawc.epa.gov/summary/visual/assessment/100500347/N-Trifluoroacetyl-2-aminoethanol-DTXSID10220035/ |
| **Not Specified in Title/Abstract** | N/A | https://hawc.epa.gov/summary/visual/assessment/100500347/Not-Specified-in-TitleAbstract/ |
| **Octafluorocyclobutane**  DTXSID9041811 | N/A | https://hawc.epa.gov/summary/visual/assessment/100500347/Octafluorocyclobutane-DTXSID9041811/ |
| **Pentafluoroiodoethane**  DTXSID8040149 | N/A | https://hawc.epa.gov/summary/visual/assessment/100500347/Pentafluoroiodoethane-DTXSID8040149/ |
| **Perflenapent**  DTXSID3046613 | N/A | https://hawc.epa.gov/summary/visual/assessment/100500347/Perflenapent-DTXSID3046613/ |
| **Perflunafene**  DTXSID0046511 | N/A | https://hawc.epa.gov/summary/visual/assessment/100500347/Perflunafene-DTXSID0046511/ |
| **Perfluoro tert-butylcyclohexane**  DTXSID70233868 | N/A | https://hawc.epa.gov/summary/visual/assessment/100500347/Perfluoro-tert-butylcyclohexane-DTXSID70233868/ |
| **Perfluoro(2-propoxypropyl vinyl ether)**  DTXSID30880150 | N/A | https://hawc.epa.gov/summary/visual/assessment/100500347/Perfluoro2-propoxypropyl-vinyl-ether-DTXSID30-50a7/ |
| **Perfluoro-2-methoxyaceticacid**  DTXSID00408562 | N/A | https://hawc.epa.gov/summary/visual/assessment/100500347/Perfluoro-2-methoxyaceticacid-DTXSID00408562/ |
| **Perfluoro-3,5,7-trioxaoctanoic acid**  DTXSID20892348 | N/A | https://hawc.epa.gov/summary/visual/assessment/100500347/Perfluoro-357-trioxaoctanoic-acid-DTXSID20892348/ |
| **Perfluoro-3,5-dioxahexanoic acid**  DTXSID50892351 | N/A | https://hawc.epa.gov/summary/visual/assessment/100500347/Perfluoro-35-dioxahexanoic-acid-DTXSID50892351/ |
|  |  |  |
| **Perfluorobiphenyl**  DTXSID7075341 | N/A | https://hawc.epa.gov/summary/visual/assessment/100500347/Perfluorobiphenyl-DTXSID7075341/ |
|  |  |  |
| **Perfluorododecanoate**  DTXSID00892482 | N/A | https://hawc.epa.gov/summary/visual/assessment/100500347/Perfluorododecanoate-DTXSID00892482/ |
| **Perfluoroheptanoate**  DTXSID60892483 | N/A | https://hawc.epa.gov/summary/visual/assessment/100500347/Perfluoroheptanoate-DTXSID60892483/ |
| **Perfluorohexadecanoic acid**  DTXSID1070800 | N/A | https://hawc.epa.gov/summary/visual/assessment/100500347/Perfluorohexadecanoic-acid-DTXSID1070800/ |
| **Perfluorohexadecyl iodide**  DTXSID5059878 | N/A | https://hawc.epa.gov/summary/visual/assessment/100500347/Perfluorohexadecyl-iodide-DTXSID5059878/ |
| **Perfluorohexane**  DTXSID7046548 | N/A | https://hawc.epa.gov/summary/visual/assessment/100500347/Perfluorohexane-DTXSID7046548/ |
| **Perfluorohexyl hydrogen sulfate**  DTXSID901034835 | N/A | https://hawc.epa.gov/summary/visual/assessment/100500347/Perfluorohexyl-hydrogen-sulfate-DTXSID901034835/ |
| **Perfluorohexyl phosphonic acid**  DTXSID90880439 | N/A | https://hawc.epa.gov/summary/visual/assessment/100500347/Perfluorohexyl-phosphonic-acid-DTXSID90880439/ |
| **Perfluoroisobutene**  DTXSID4073176 | N/A | https://hawc.epa.gov/summary/visual/assessment/100500347/Perfluoroisobutene-DTXSID4073176/ |
|  |  |  |
| **Perfluorooctyl iodide**  DTXSID0060147 | N/A | https://hawc.epa.gov/summary/visual/assessment/100500347/Perfluorooctyl-iodide-DTXSID0060147/ |
|  |  |  |
| **Perfluoropentanoate**  DTXSID00892487 | N/A | https://hawc.epa.gov/summary/visual/assessment/100500347/Perfluoropentanoate-DTXSID00892487/ |
| **Perfluoropropanesulfonic acid**  DTXSID30870531 | N/A | https://hawc.epa.gov/summary/visual/assessment/100500347/Perfluoropropanesulfonic-acid-DTXSID30870531/ |
| **Perfluorotetradecahydrophenanthrene**  DTXSID1047029 | N/A | https://hawc.epa.gov/summary/visual/assessment/100500347/Perfluorotetradecahydrophenanthrene-DTXSID1047029/ |
| **Perfluorotetradecanoate**  DTXSID60892488 | N/A | https://hawc.epa.gov/summary/visual/assessment/100500347/Perfluorotetradecanoate-DTXSID60892488/ |
| **Perfluorotributylamine**  DTXSID0027141 | N/A | https://hawc.epa.gov/summary/visual/assessment/100500347/Perfluorotributylamine-DTXSID0027141/ |
| **Perfluorotridecanoate**  DTXSID20892489 | N/A | https://hawc.epa.gov/summary/visual/assessment/100500347/Perfluorotridecanoate-DTXSID20892489/ |
| **Perfluoroundecanoate**  DTXSID30892475 | N/A | https://hawc.epa.gov/summary/visual/assessment/100500347/Perfluoroundecanoate-DTXSID30892475/ |
| **Perflutren**  **DTXSID9052503** | N/A | https://hawc.epa.gov/summary/visual/assessment/100500347/Perflutren-DTXSID9052503/ |
| **Perftoran**  **DTXSID50244230** | N/A | https://hawc.epa.gov/summary/visual/assessment/100500347/Perftoran-DTXSID50244230/ |
| **PFAS Universe 2024** | N/A | https://hawc.epa.gov/summary/visual/assessment/100500347/pfas-universe-2024/ |
| **PFAS Universe Tableau Dashboard** | N/A | https://hawc.epa.gov/summary/visual/assessment/100500347/PFAS-Universe-Tableau-Dashboard/ |
| **Potassium 11-chloroeicosafluoro-3-oxaundecane-1-sulfonate**  DTXSID00892447 | N/A | https://hawc.epa.gov/summary/visual/assessment/100500347/Potassium-11-chloroeicosafluoro-3-oxaundecane-5714/ |
| **Potassium N-ethyl-N-((heptadecafluorooctyl)sulphonyl)glycinate**  DTXSID3042009 | N/A | https://hawc.epa.gov/summary/visual/assessment/100500347/Potassium-N-ethyl-N-heptadecafluorooctylsulph-f00f/ |
| **Potassium trifluoroacetate**  DTXSID00951841 | N/A | https://hawc.epa.gov/summary/visual/assessment/100500347/Potassium-trifluoroacetate-DTXSID00951841/ |
| **Pyrifluquinazon**  DTXSID6058057 | N/A | https://hawc.epa.gov/summary/visual/assessment/100500347/Pyrifluquinazon-DTXSID6058057/ |
| **S-(1,1,2,2-Tetrafluoroethyl)cysteine**  DTXSID20915283 | N/A | https://hawc.epa.gov/summary/visual/assessment/100500347/S-1122-Tetrafluoroethylcysteine-DTXSID20915283/ |
| **Sodium perfluoroheptane sulfonate**  DTXSID401032634 | N/A | https://hawc.epa.gov/summary/visual/assessment/100500347/Sodium-perfluoroheptane-sulfonate-DTXSID401032634/ |
| **Sodium trifluoroacetate**  DTXSID0062715 | N/A | https://hawc.epa.gov/summary/visual/assessment/100500347/Sodium-trifluoroacetate-DTXSID0062715/ |
| **Tecarfarin**  DTXSID90235788 | N/A | https://hawc.epa.gov/summary/visual/assessment/100500347/Tecarfarin-DTXSID90235788/ |
| **Tetraconazole**  DTXSID8034956 | N/A | https://hawc.epa.gov/summary/visual/assessment/100500347/Tetraconazole-DTXSID8034956/ |
| **Tiflamizole**  DTXSID90212059 | N/A | https://hawc.epa.gov/summary/visual/assessment/100500347/Tiflamizole-DTXSID90212059/ |
| **trans-Perfluorodecalin**  DTXSID201015480 | N/A | https://hawc.epa.gov/summary/visual/assessment/100500347/trans-Perfluorodecalin-DTXSID201015480/ |
| **Trifluoro(trifluoromethoxy)ethylene**  DTXSID3051599 | N/A | https://hawc.epa.gov/summary/visual/assessment/100500347/Trifluorotrifluoromethoxyethylene-DTXSID3051599/ |
| **Trifluoromethanesulfonic anhydride**  DTXSID501014629 | N/A | https://hawc.epa.gov/summary/visual/assessment/100500347/Trifluoromethanesulfonic-anhydride-DTXSID501014629/ |

# References

1. Carlson LA, Angrish M, Shirke AV, Radke EG, Schulz B, Kraft A, Judson R, Patlewicz G, Blain R, Lin C, et al. Systematic evidence map for over one hundred and fifty per- and polyfluoroalkyl substances (PFAS). Environ Health Perspect. 2022; 130(5):56001. <https://doi.org/10.1289/ehp10343>

2. Carlson LA, Angrish M, Shirke AV, Radke EG, Schulz B, Kraft A, Judson R, Patlewicz G, Blain R, Lin C, et al. Erratum: Systematic evidence map for over one hundred and fifty per- and polyfluoroalkyl substances (PFAS). 2024; 132(1:019001). <https://doi.org/10.1289/ehp14191>

3. Radke E, Wright MJ, Christensen K, Lin CJ, Goldstone AE, Glenn B, Thayer K. Epidemiology evidence for health effects of 150 per- and polyfluoroalkyl substances: A systematic evidence map. Environ Health Perspect. 2022; 130(9):96003. <https://doi.org/10.1289/ehp11185>

4. Shirke AV, Radke EG, Lin C, Blain R, Vetter N, Lemeris C, Hartman P, Hubbard H, Angrish M, Arzuaga X, et al. Expanded systematic evidence map for hundreds of per- and polyfluoroalkyl substances (PFAS) and comprehensive PFAS human health dashboard. Environ Health Perspect. 2024; 132(2):026001. <https://doi.org/10.1289/ehp13423>

5. U.S. Environmental Protection Agency (U.S. EPA). Health Assessment Workspace Collaborative (HAWC): ORD SEM PFAS Universe. 2024. <https://hawc.epa.gov/assessment/100500347/>

6. U.S. Environmental Protection Agency (U.S. EPA). Health Assessment Workspace Collaborative (HAWC). Washington, DC. 2021. <https://hawc.epa.gov/portal/>

7. U.S. Environmental Protection Agency (U.S. EPA). ORD staff handbook for developing IRIS assessments. Washington, DC: U.S. Environmental Protection Agency, Office of Research and Development, Center for Public Health and Environmental Assessment; 2022. EPA 600/R-22/268. <https://cfpub.epa.gov/ncea/iris_drafts/recordisplay.cfm?deid=356370>

8. Thayer KA, Angrish M, Arzuaga X, Carlson LM, Davis A, Dishaw L, Druwe I, Gibbons C, Glenn B, Jones R, et al. Systematic Evidence Map (SEM) template: Report format and methods used for the US EPA Integrated Risk Information System (IRIS) program, Provisional Peer-Reviewed Toxicity Value (PPRTV) program, and other "fit for purpose" literature-based human health analyses. Environ Int. 2022; 169:107468. <https://doi.org/10.1016/j.envint.2022.107468>

9. Keshava C, Davis JA, Stanek J, Thayer KA, Galizia A, Keshava N, Gift J, Vulimiri SV, Woodall G, Gigot C, et al. Application of systematic evidence mapping to assess the impact of new research when updating health reference values: A case example using acrolein. Environ Int. 2020; 143:105956. <https://doi.org/10.1016/j.envint.2020.105956>

10. U.S. Environmental Protection Agency (U.S. EPA). Systematic Review Protocol for the PFBA, PFHxA, PFHxS, PFNA, and PFDA (anionic and acid forms) IRIS Assessments: Supplemental Information―Appendix A. 2021. EPA/635/R-19/050. <https://cfpub.epa.gov/ncea/iris_drafts/recordisplay.cfm?deid=345065>

11. Magnuson K, Cawley M, Reilly D, Varghese A. Improving efficiency of systematic reviews through machine learning for automated record deduplication and text analytics for iterative keyword streamlining. San Antonio, Texas; 2018.

12. U.S. Environmental Protection Agency (U.S. EPA). IRIS toxicological review of perfluorobutanoic acid (PFBA) and related salts. 2022. EPA/635/R-22/277F. <https://cfpub.epa.gov/ncea/iris_drafts/recordisplay.cfm?deid=350051>

13. U.S. Environmental Protection Agency (U.S. EPA). Toxicological Review of Perfluorohexanoic Acid (PFHxA) and Related Salts (Final Report, 2023). 2023. EPA/635/R-23/027F. <https://cfpub.epa.gov/ncea/iris_drafts/recordisplay.cfm?deid=357314>

14. U.S. Environmental Protection Agency (U.S. EPA). Final human health toxicity assessment for perfluorooctanoic acid (PFOA) and related salts. Washington, DC: U.S. Environmental Protection Agency, Office of Water, Health and Ecological Criteria Division; 2024. <https://www.epa.gov/system/files/documents/2024-04/main_final-toxicity-assessment-for-pfoa_2024-04-09-refs-formatted.pdf>

15. U.S. Environmental Protection Agency (U.S. EPA). IRIS toxicological review of perfluorononanoic acid (PFNA) and related salts (Public comment and external review draft). Washington, DC; 2024. EPA/635/R-24/031. <https://iris.epa.gov/document/&deid=355409>

16. U.S. Environmental Protection Agency (U.S. EPA). Toxicological review of perfluorodecanoic acid (PFDA) and related salts (Final report, 2024). Washington, DC; 2024. EPA/635/R-24/172Fa. <https://iris.epa.gov/document/&deid=361797>?

17. U.S. Environmental Protection Agency (U.S. EPA). Human health toxicity values for perfluorobutane sulfonic acid (CASRN 375-73-5) and related compound potassium perfluorobutane sulfonate (CASRN 29420-49-3). Washington, DC: U.S. Environmental Protection Agency, Office of Research and Development; 2021. EPA/600/R-20/345F. <https://cfpub.epa.gov/ncea/risk/recordisplay.cfm?deid=350888>

18. U.S. Environmental Protection Agency (U.S. EPA). Toxicological review of perfluorohexanesulfonic acid (PFHxS) and related salts: Final report, 2025. 2025. EPA/635/R-25/012. <https://iris.epa.gov/document/&deid=363894>

19. U.S. Environmental Protection Agency (U.S. EPA). Final human health toxicity assessment for perfluorooctane sulfonic acid (PFOS) and related salts. Washington, DC: U.S. Environmental Protection Agency, Office of Water, Health and Ecological Criteria Division; 2024. <https://www.epa.gov/system/files/documents/2024-04/main_final-toxicity-assessment-for-pfos_2024-04-09-refs-formatted_508c.pdf>

20. U.S. Environmental Protection Agency (U.S. EPA). Human health toxicity values for hexafluoropropylene oxide (HFPO) dimer acid and its ammonium salt (CASRN 13252-13-6 and CASRN 62037-80-3). Also known as "GenX chemicals." Final report. Washington, DC: U.S. Environmental Protection Agency, Office of Water; 2021. EPA-822R-21-010. <https://www.epa.gov/system/files/documents/2021-10/genx-chemicals-toxicity-assessment_tech-edited_oct-21-508.pdf>

21. U.S. Environmental Protection Agency (U.S. EPA). ORD human health toxicity value for lithium bis [(trifluoromethyl)sulfonyl]azanide (HQ-115). 2023. EPA/600/R-22/195F. <https://cfpub.epa.gov/si/si_public_record_Report.cfm?dirEntryId=358288&Lab=CPHEA>

22. U.S. Environmental Protection Agency (U.S. EPA). IRIS Program Outlook. 2020. <https://www.epa.gov/iris/iris-program-outlook>
